# Theoretical study of the antioxidant mechanism and structure-activity relationships of 1,3,4-oxadiazol-2-ylthieno[2,3-d]pyrimidin-4-amine derivatives: a computational approach

**DOI:** 10.3389/fchem.2024.1443718

**Published:** 2024-07-30

**Authors:** Ahmed H. Bakheit, Tanveer A. Wani, Abdulrahman A. Al-Majed, Hamad M. Alkahtani, Manal M. Alanazi, Fahad Rubayyi Alqahtani, Seema Zargar

**Affiliations:** ^1^ Department of Pharmaceutical Chemistry, College of Pharmacy, King Saud University, Riyadh, Saudi Arabia; ^2^ Department of Biomedical Technology, College of Applied Medical Sciences, King Saud University, Riyadh, Saudi Arabia; ^3^ Department of Biochemistry, College of Science, King Saud University, Riyadh, Saudi Arabia

**Keywords:** antioxidants, thieno[2, 3-d]pyrimidine derivatives, density functional theory (DFT), hydrogen atom transfer (HAT), free radicals, single electron transfer-proton transfer (SET-PT), reactivity descriptors

## Abstract

A theoretical thermodynamic study was conducted to investigate the antioxidant activity and mechanism of 1,3,4-oxadiazol-2-ylthieno[2,3-d]pyrimidin-4-amine derivatives (OTP) using a Density Functional Theory (DFT) approach. The study assessed how solvent environments influence the antioxidant properties of these derivatives. With the increasing prevalence of diseases linked to oxidative stress, such as cancer and cardiovascular diseases, antioxidants are crucial in mitigating the damage caused by free radicals. Previous research has demonstrated the remarkable scavenging abilities of 1,3,4-oxadiazole derivatives, prompting this investigation into their potential using computational methods. DFT calculations were employed to analyze key parameters, including bond dissociation enthalpy (BDE), ionization potential (IP), proton dissociation enthalpy (PDE), and electron transfer enthalpy (ETE), to delineate the antioxidant mechanisms of these compounds. Our findings indicate that specific electron-donating groups such as amine on the phenyl rings significantly enhance the antioxidant activities of these derivatives. The study also integrates global and local reactivity descriptors, such as Fukui functions and HOMO-LUMO energies, to predict the stability and reactivity of these molecules, providing insights into their potential as effective synthetic antioxidants in pharmaceutical applications.

## 1 Introduction

Free radicals, highly reactive molecules with unpaired electrons, are constantly generated in living organisms through normal metabolic processes and exposure to environmental factors like pollution and radiation (Niki, 2016). Excessive free radical production can lead to oxidative stress, damaging cellular components such as DNA, proteins, and lipids, and contributing to various chronic diseases like cancer, cardiovascular diseases, and neurodegenerative disorders. Antioxidants play a crucial role in mitigating oxidative stress by scavenging free radicals and preventing their damaging effects.

In this study the antioxidant properties of OTP derivatives were explored using computational methods. *In vitro* assays of OTP derivatives have established the free radicals neutralizing capacity of OTP using DPPH, hydrogen peroxide, and nitric oxide radical scavenging methods. Further, the presence of electron-donating groups like difluoro, fluoro, and chloro-fluoro on the phenyl rings, enhanced their effectiveness when compared to ascorbic acid (Kotaiah et al., 2012).

Natural and synthetic antioxidants have garnered significant attention for their potential to prevent or treat oxidative stress-related diseases. Among synthetic antioxidants, thieno[2,3-d]pyrimidine derivatives have emerged as promising candidates due to their diverse biological activities, including anti-inflammatory (Rizk et al., 2012), kinase inhibition (Bugge et al., 2016), anticancer (Bozorov et al., 2015; Mavrova et al., 2016), antiviral (Bassetto et al., 2016), antituberculosis (Li et al., 2015) and antimicrobial properties (El-Sayed et al., 2012; Kotaiah et al., 2012). The presence of nitrogen and sulfur atoms in the thieno[2,3-d]pyrimidine core structure suggests potential antioxidant activity through mechanisms like hydrogen atom transfer (HAT), single electron transfer-proton transfer (SETPT), and sequential proton loss electron transfer (SPLET).

Computational methods like density functional theory (DFT) offer a powerful tool to investigate the antioxidant potential of molecules. DFT allows for the calculation of thermochemical parameters such as bond dissociation enthalpy (BDE), ionization potential (IP), proton dissociation enthalpy (PDE), and electron transfer enthalpy (ETE), which are key indicators of antioxidant activity through different mechanisms. Additionally, DFT can provide insights into molecular properties like HOMO-LUMO energy gap, Fukui functions, and molecular electrostatic potential (MEP), further characterizing the reactivity and stability of potential antioxidants.

Inspired by the promising biological activities of thieno[2,3-d]pyrimidine derivatives (Kotaiah et al., 2012) and the need for effective antioxidants, this study aims to further investigate the antioxidant potential of these compounds using DFT calculations. By analyzing various thermochemical parameters and molecular properties, we aim to identify the most promising antioxidant candidates within this class of compounds and elucidate the mechanisms underlying their activity. This research could pave the way for the development of novel and effective antioxidants for the prevention and treatment of oxidative stress-related diseases (Lobo et al., 2010; Leopoldini et al., 2011).

Our investigation into the antioxidant properties of these compounds employs a comprehensive computational strategy. The initial step involves calculating key thermochemical parameters, including bond dissociation enthalpy (BDE), proton affinity (PA), electron transfer enthalpy (ETE), and ionization energy (IE), using density functional theory (DFT). These calculations are performed at the (RO) B3LYP/6-311G(d,p) level of theory, considering both gas phase and solvent environments (methanol and water, with a dielectric constant (ε) of 78.36 for water).

Subsequently, we explored global descriptive parameters such as chemical potential (μ), chemical hardness (η), and global electrophilicity (ω) for the neutral compounds. These parameters provide valuable insights into the reactivity and stability of the molecules, further characterizing their antioxidant potential.

To gain a deeper understanding of the free radical scavenging mechanisms, we constructed potential energy surfaces (PES) for all possible addition and hydrogen abstraction reactions between the most promising antioxidant candidates and the hydroperoxyl radicals HOO^•^ and HO^•^ radicals. This analysis allows us to identify the most favorable reaction pathways and elucidate the energetic aspects of the scavenging process.

Concurrently, we perform a detailed examination of the singly occupied molecular orbital (SOMO), and atomic spin density (ASD) analyses of the optimized molecules. These analyses provide a clear picture of the electronic and structural changes occurring during the reaction, offering a comprehensive explanation of the reaction mechanisms at the molecular level.

## 2 Material and methods

### 2.1 Compounds

**SCHEME 1:**
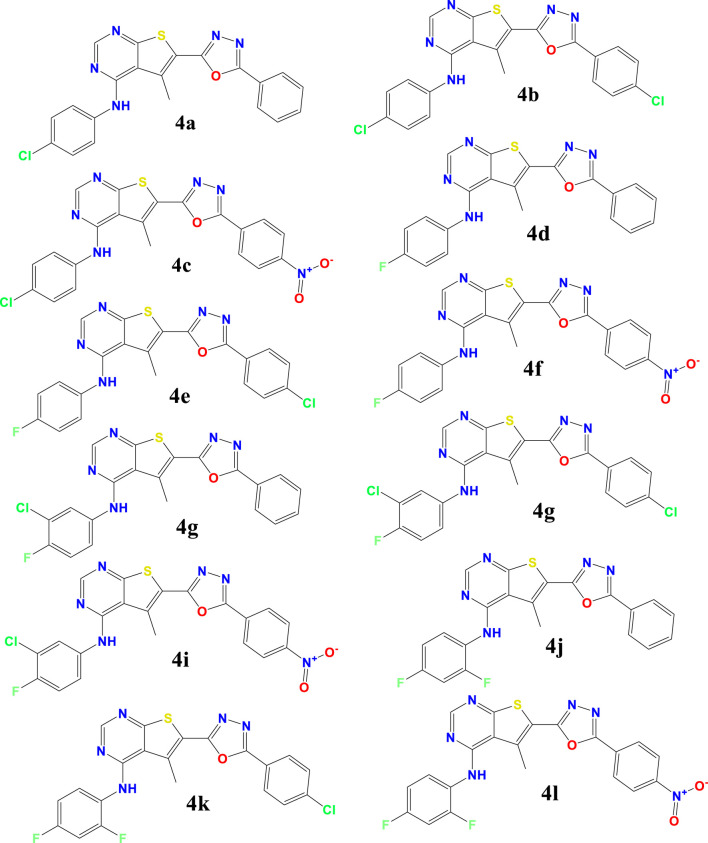
Preparation of Compounds 4a-l: 1,3,4-Oxadiazole-Substituted Thieno[2,3-d]pyrimidines.

The investigated compounds 4a-4l, were synthesized and tested the antioxidant properties of a series of thieno[2,3-d]pyrimidine derivatives by Kotaiah et al. (2012), assessing their antioxidant capabilities via standard *in vitro* assays. The structures of these compounds are detailed in Scheme 1 of their publication. Our study builds on their work by conducting Density Functional Theory (DFT) calculations to explore the electronic properties and theoretical reactivity of these compounds.

### 2.2 Theoretical background

#### 2.2.1 Computational method

All molecular structures were initially drawn in two dimensions (2D) using MarvinSketch Draw (https://chemaxon.com/marvin) and subsequently converted to three-dimensional (3D) structures using MarvinSketch. The 3D structures were saved in the mol2 file format.

**SCHEME 2:**
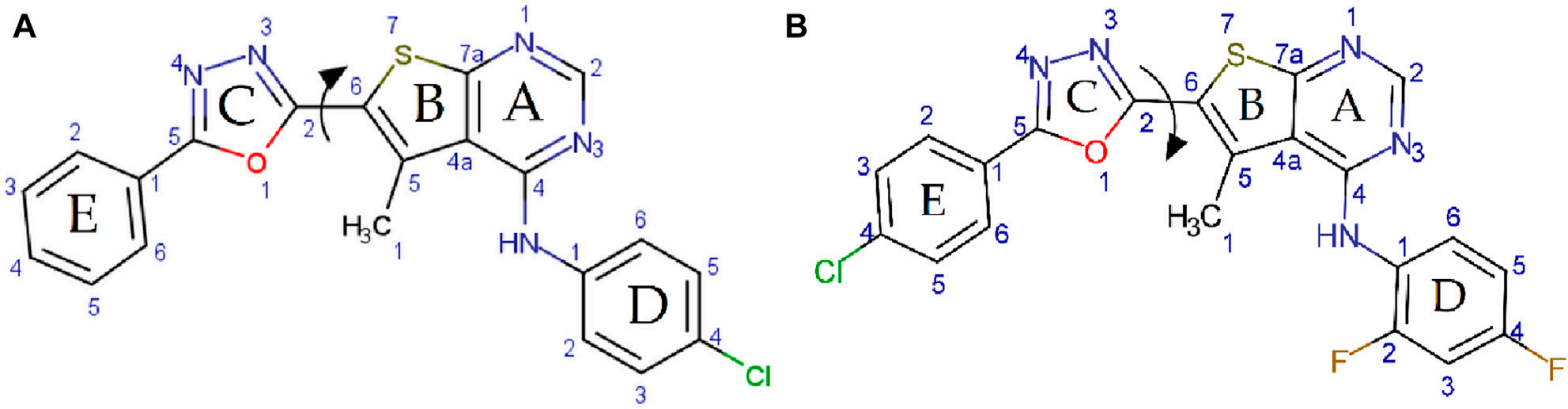
Potential energy surface scans for panel **(A)** compounds 4a and panel **(B)** 4K, exploring the O1–C2–C6–S7 dihedral angle.

Preliminary energy minimization and geometry optimization were performed using the Universal Force Field (UFF) as implemented in the Molecular Mechanics program (MM+) within Gaussian. To identify the lowest energy conformations, potential energy profiles were generated by scanning the torsion angle (τ) between rings B and C, defined by the dihedral angle of the (N3–C2–C6–S7) atoms (Scheme 2). The torsion angle (τ) was systematically varied without constraints using the UFF force field.

Following the identification of minimum energy conformations, further geometry optimization was conducted using Density Functional Theory (DFT) using the Gaussian 09 software package (Frisch et al., 2009) at the B3LYP/6-311G(d,p) level of theory. This level of theory combines Becke’s three-parameter hybrid exchange functional (B3) (Becke, 1988) with the Lee-Yang-Parr correlation functional (LYP) (Lee et al., 1988) and employs the 6-311G(d,p) basis set (Stephens et al., 1994; Devlin et al., 1995; Sezgin et al., 2022). Molecular enthalpies for each compound were computationally determined at a standard temperature of 298.15 K and 1 atmosphere of pressure.

The influence of solvation on molecular properties was investigated for 4K and ascorbic acid using the self-consistent reaction field (SCRF) method, specifically the polarizable continuum model (PCM) with the integral equation formalism variant (IEFPCM). Single-point energy calculations with the IEFPCM model (Barone and Cossi, 1998) were deemed sufficient to describe the solvation effects of benzene and water due to the conformational rigidity of the molecules (Miertuš et al., 1981; Miertus and Tomasi, 1982; Cossi et al., 1996; Barone et al., 1997).

In this study, the electronic properties of the series of compounds were analysed using Density Functional Theory (DFT) at the B3LYP/6-311++G(d,p) level of theory. The properties calculated include vertical ionization potential (IP), electron affinity (EA), electronegativity (χ), hardness (η), softness (S), and electrophilicity index (ω).

#### 2.2.2 Radical scavenging pathways

To identify the weakest bond positions within the studied compounds, the semi-empirical PM6 method was initially utilized to calculate the bond dissociation enthalpy (BDE) for all potential N–H and C–H bond cleavages. Based on these preliminary calculations, the bonds exhibiting the lowest BDEs were selected for further analysis.

Subsequently, harmonic vibrational frequency calculations were performed at the B3LYP/6-311G(d,p) level (Senthilkumar, 2021; Sezgin et al., 2022) of theory to obtain thermal enthalpies for the optimized geometries neutral (RNH), radical (RN), cationic radical (RNH^+^), and anionic (RN^–^) forms of each compound. These enthalpies were then used to evaluate three antioxidant reaction mechanisms: Hydrogen Atom Transfer (HAT), Single Electron Transfer-Proton Transfer (SET-PT), and Sequential Proton Loss Electron Transfer (SPLET). The HAT reaction enthalpies were calculated using Supplementary Equations S1, S6 based on bond dissociation enthalpy (BDEs). Supplementary Equations S2, S3, S7, S8, employing ionization potentials (IPs) and proton dissociation energies (PDEs), were used to determine the SET-PT reaction enthalpies. The SPLET reaction enthalpies were calculated using Supplementary Equations S4, S5, S9, S10 based on proton affinities (PAs) and electron transfer energies (ELETs) (Dimić et al., 2017; Bakheit et al., 2022a; Bakheit et al., 2022b; Bakheit et al., 2023).

For the radical species (RN and RNH^+^), the calculations were carried out using the unrestricted open-shell approach. The wavefunctions of these radicals were examined for spin contamination, with the expectation that the ⟨*S* ^2^⟩ values would be approximately 0.750, confirming the predominance of doublet states (Carpenter and Weinhold, 1988).

In the gas phase, the enthalpy of the hydrogen atom was determined to be −0.5 Hartree. This value was consistently obtained across various environments using the same computational approach. Enthalpy values for the electron (e^−^) and proton (H^+^) were adopted from previously published works, specifically cited in references (Parker, 1992; Klein and Lukeš, 2006; Marković et al., 2016). For the vibrational frequencies, calculations performed at the B3LYP/6-311G(d,p) levels were adjusted using scaling factors of 0.9669, (Irikura et al., 2005; Alecu et al., 2010).

#### 2.2.3 Reactivity descriptors

Conceptual Density Functional Theory (DFT) provides insights into the chemical reactivity of molecules through global and local reactivity descriptors. This document incorporates a theoretical foundation consistent with earlier studies referenced in (Sastre et al., 2017; Farrokhnia, 2020; Bakheit et al., 2022a; Mostafa et al., 2023) to ensure thorough understanding. As established in the conceptual framework of Density Functional Theory (DFT), cited in references (Abuelizz et al., 2021a; Mostafa et al., 2021; Ghabbour et al., 2022), Global hardness (η), which quantifies the resistance of a molecule to changes in its electron number, is defined as the second derivative of the energy (E) with respect to the number of electrons (N) at a constant external potential $v\left(\vec{r}\right)$ . This relationship, originally proposed by Parr and Pearson, is expressed mathematically in the Eq. 1 as:

$$\eta ={\left(\frac{\partial^{2}E}{\partial N^{2}}\right)}_{v\left(\vec{r}\right)}$$ (1)

where *μ* is the electronic chemical potential of an N-electron system in the presence of an external potential $v\left(\vec{r}\right)$ . The formula for *μ* is given by in the following Eq. 2:

$$\mu ={\left(\frac{\partial E}{\partial N}\right)}_{v\left(\vec{r}\right)}$$ (2)

Hardness serves as an important descriptor of molecular reactivity by measuring the resistance to changes in the electron distribution of the system. Molecules with higher values of η are typically less reactive. Originally, the factor 1/2 in the hardness equation was intended to provide symmetry with the chemical potential equation, but it has since been removed.

Using a finite-difference approximation, the Eqs 3, 4 for calculating μ and η, respectively, are:

$$\eta =\frac{E_{N+1}-2E_{N}+E_{N-1}}{2}=\frac{\left(IP-EA\right)}{2}$$ (3)

$$\chi =-\mu \approx \frac{E_{N-1}-E_{N+1}}{2}=\frac{\left(IP+EA\right)}{2}$$ (4)

According to “Koopmans’ theorem,” the ionization potential (IP) and electron affinity (EA) can be approximated in the Eqs 4, 5, respectively, as:

$$IP=\in_{HOMO}$$ (5)

$$EA=\in_{LUMO}$$ (6)

Thus, the Eqs 7, 8 for calculating μ and η, respectively using frontier molecular orbital energies are:

$$\mu =\frac{{-\epsilon }_{HOMO}+\epsilon_{LUMO}}{2}$$ (7)

$$\eta =\frac{\in_{HOMO}+\in_{LUMO}}{2}$$ (8)

where ∈ denotes the energy of the corresponding frontier molecular orbital. The concept of electronegativity (χ) is related to 𝜇, and global softness (S) is defined as the inverse of global hardness (η), emphasizing its role in describing the flexibility of electron distribution within the molecule.

The Fukui function, denoted as *f*(𝑟), is mathematically expressed in Eq. 9 through the derivative of the electron density *ρ*(𝑟) with respect to the electron count N, as outlined in reference (Geerlings et al., 2003):

$$f\left(r\right)={\left(\frac{\partial \rho \left(r\right)}{\partial N}\right)}_{v\left(r\right)}$$ (9)

Function *f*(𝑟) indicates a molecular site’s capacity to gain or lose electrons. Areas of a molecule showing higher *f*(𝑟) values are typically more reactive (Geerlings et al., 2003). Implementing a finite difference approximation, the following two Eqs 10, 11 derived of the Fukui function based on changes in the electronic density:

$$f^{+}\left(r\right)=\rho_{N+1}\left(r\right)-\rho_{N}\left(r\right)$$ (10)

$$f^{-}\left(r\right)=\rho_{N}\left(r\right)-\rho_{N-1}\left(r\right)$$ (11)

where 𝜌_𝑁+1_(𝑟), 𝜌_𝑁_(𝑟), and 𝜌_𝑁−1_(𝑟) are the electron densities at location 𝑟r for systems containing 𝑁+1, 𝑁, and 𝑁−1 electrons respectively. The 𝑓^+^(𝑟) function is associated with nucleophilic reactions, whereas 𝑓^−^(𝑟) pertains to electrophilic reactions (Ayers et al., 2007).

Furthermore, Morell et al. (Morell et al., 2005; Morell et al., 2006; Toro-Labbé, 2006) proposed a local reactivity descriptor known as the dual descriptor (DD), ^(2)^(𝑟) or Δ𝑓(𝑟), which is defined in the Eq. 12 as:

$$∆f\left(r\right)=\frac{\partial f\left(r\right)}{\partial N}$$ (12)

This descriptor differentiates between nucleophilic and electrophilic behavior at atomic sites. When Δ𝑓𝑘>0, it indicates that atom 𝑘k behaves as an electrophile prone to nucleophilic attack, and conversely, Δ𝑓𝑘<0 indicates that atom 𝑘 behaves as a nucleophile susceptible to electrophilic attack.

In an insightful article by Jorge Ignacio Martínez-Araya (Martínez-Araya, 2015), it was demonstrated that the dual descriptor offers a more precise measure of local reactivity compared to the Fukui function. The dual descriptor effectively identifies distinct nucleophilic and electrophilic sites and is less impacted by the omission of relaxation terms, which is a limitation when using the frontier molecular orbital approximation. Consequently, this study opts for the dual descriptor over individual Fukui functions to meet its research goals.

Domingo introduced the Parr functions *P*(𝑟) in 2013 (Chamorro et al., 2013; Domingo et al., 2013), defined based on the atomic spin density (ASD) at the atom 𝑟 in the radical cation or anion states (Eqs 13, 14):

$$P^{-}\left(r\right)=\rho_{s}^{rc}\left(r\right)\left(\text{for electrophilic attacks}\right)$$ (13)

$$P^{+}\left(r\right)=\rho_{s}^{ra}\left(r\right)\;\left(\text{for nucleophilic attacks}\right)$$ (14)

These functions provide a detailed view of the ASD across each atom in the radical cation and anion states, shedding light on the local reactivity traits of the neutral molecule (Sastre et al., 2017).

## 3 Results and discussion

### 3.1 Optimization of molecular and radical geometries

The research focused on understanding the properties of antioxidant molecules, specifically OTP derivatives. Both amino aromatic groups and aromatic rings are crucial for antioxidant activity. In this study, molecules with two NH groups attached to the aromatic ring exhibit significantly lower IC50 values and stoichiometric parameters compared to those with only one amino group. When thieno[2,3-d]pyrimidines and 1,3,4-oxadiazoles are present in these compounds, high antiradical activity is anticipated due to the ease of proton donation from these groups and the additional stabilization of the resulting radicals. Computational methods were employed for an in-depth analysis of the structural and electronic parameters influencing antioxidant activity, as detailed in the following sections (Lü et al., 2010; Kotaiah et al., 2012; Vásquez-Espinal et al., 2019).

**FIGURE 1:**
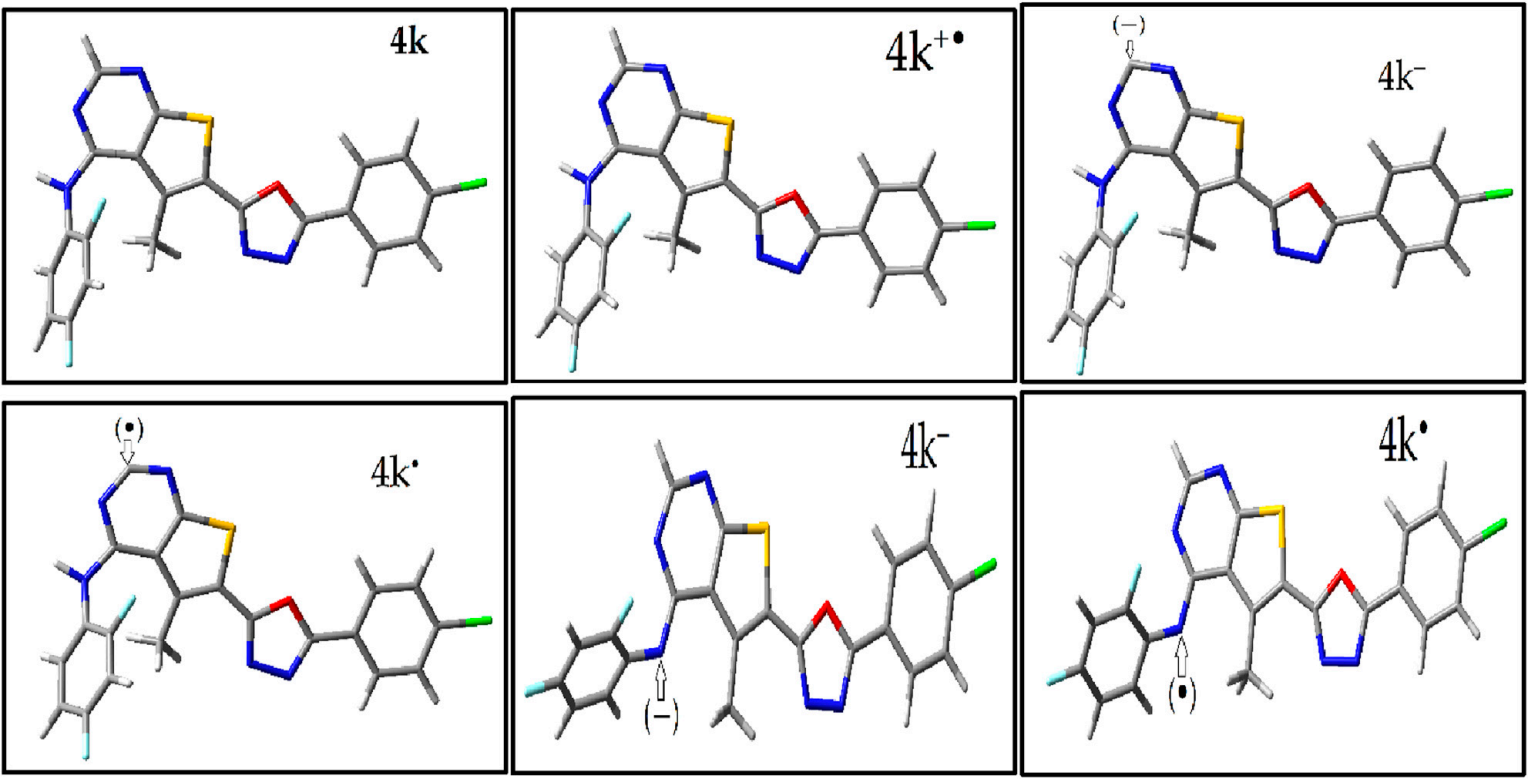
Optimized Geometries of Compound 4k’s Most Stable Structures. This figure displays the neutral, anionic, radical anion, cationic, and radical cation forms of compound 4k, optimized using B3LYP/6-311G(d,p) level of theory.

The study involved optimizing the molecular structures of these compounds and their Radicals through two main steps: initial optimization using the MM + force field within the Gaussian software, followed by further refinement with density functional theory (DFT) at the B3LYP/6-311g(d,p) level (Supplementary Figure S5) (Sadasivam and Kumaresan, 2011). The most stable structures obtained from these optimizations were then selected for additional examinations, specifically to generate geometries for radicals. The findings present the most stable optimized geometries of compound 4k—including its neutral molecule, free radical cation form, radical anion forms, and anion forms—in Figure 1. The neutral structures of the remaining compounds (4a-4l) are shown in Supplementary Figure S1 with Including Electronic Energy (EE) and Thermal Free Energy Correction (TEC).

### 3.2 Global reactivity descriptors

The calculated properties presented in Table 1 suggest that the investigated compounds exhibit a tendency to donate electrons rather than accept them. This behavior is consistent with their potential antioxidant activity, as antioxidants often function by donating electrons to neutralize free radicals. Further insights into their reactivity can be gleaned by examining the energy gap (HOMO-LUMO gap) of these compounds, a crucial parameter that reflects the energy difference between the highest occupied molecular orbital (HOMO) and the lowest unoccupied molecular orbital (LUMO). A smaller energy gap generally corresponds to greater reactivity and a higher propensity for electron donation.

**TABLE 1:** Vertical ionization energy (IE), electron affinity (EA), chemical potential (μ), Electronegativity (X)chemical hardness (η), global electrophilicity (ω), global Nucleophilicity (N), dipole moment (Debye), and Polarizability (α) (a.u.) calculated at B3LYP/6-311G(d,p).

Compounds	IE (eV)	EA (eV)	Μ (eV)	X (eV)	S (eV)	Η (eV^−1^)	ω (eV)	N (eV)	Dipole moment (D)	Polarizability (α) (a.u.)
4a	7.135	0.766	−3.951	3.951	0.157	6.369	1.225	3.247	1.456	329.99
4b	7.19	0.922	−4.056	4.056	0.16	6.267	1.312	3.167	2.026	349.79
4c	**7.404**	1.515	−4.459	**4.459**	**0.17**	**5.889**	**1.688**	2.992	**5.538**	**360.34**
4d	7.103	0.697	−3.9	3.9	0.156	6.407	1.187	3.309	0.738	314.76
4e	7.16	0.857	−4.009	4.009	0.159	6.303	1.275	3.227	2.026	334.40
4f	**7.378**	1.471	−4.425	**4.425**	**0.169**	**5.907**	**1.657**	3.051	**5.774**	**345.30**
4g	7.198	0.774	−3.986	3.986	0.156	6.423	1.237	3.2	1.934	325.50
4h	7.252	0.928	−4.09	4.09	0.158	6.324	1.323	3.116	2.96	345.15
4i	**7.479**	1.517	−4.498	**4.498**	**0.168**	**5.962**	**1.697**	2.926	**6.33**	**355.36**
4j	7.131	0.621	−3.876	3.876	0.154	6.51	1.154	3.301	0.903	312.93
4k	7.187	0.789	−3.988	3.988	0.156	6.398	1.243	3.215	2.805	332.58
4l	**7.417**	1.427	−4.422	**4.422**	**0.167**	**5.99**	**1.632**	3.021	**6.5456**	**343.83**
Ascorbic acid Blank	**8.69**	−1.452	−3.619	3.619	0.099	10.142	0.646	2.713	8.697	83.65

The bold values provided in Table 1 represent the most significant or important values among the data presented in those tables.

The frontier molecular orbital energies, HOMO-LUMO energy gaps (ΔE), and ionization potentials were calculated for all compounds using Density Functional Theory (DFT) at the B3LYP/6-311G(d,p) level of theory. The calculated HOMO-LUMO energy gaps (ΔE) for compounds 4a-4l are 3.75 eV, 3.70 eV, 3.18 eV, 3.75 eV, 3.70 eV, 3.15 eV, 3.78 eV, 3.74 eV, 3.25 eV, 3.83 eV, 3.77 eV, and 3.20 eV, respectively. These values are lower than that of ascorbic acid (1.84 eV, 1.74 eV, 0.70 eV, 1.84 eV, 1.74 eV, 0.64 eV, 1.91 eV, 1.82 eV, 0.84 eV, 2.00 eV, 1.88 eV, and 0.75 eV, respectively).

A smaller HOMO-LUMO gap generally indicates increased reactivity and a higher propensity for electron transfer. Within the series of investigated compounds, those with a nitro substituent at the para position of the phenyl ring tended to exhibit lower ΔE values compared to their chloro-substituted or unsubstituted counterparts. This suggests that the nitro-substituted compounds may exhibit greater reactivity, potentially acting as electrophiles due to their lower-lying LUMO energy levels.

The vertical ionization potentials (IP) and electron affinity (EA) were calculated and compiled in Table 1, with all values expressed in electron volts (eV). The chemical hardness (η) and softness (S) of the molecules were evaluated, revealing that molecules 4c, 4f, and 4i exhibit lower hardness (and thus higher softness), indicating reduced stability and enhanced reactivity in the gas phase. These findings are consistent with their increased propensity for charge-transfer mechanisms, as reflected in the softness hierarchy: Ascorbic acid < 4j < 4g < 4d = 4k < 4a < 4h < 4e < 4b < 4l < 4i < 4f < 4c.

The electronegativity (χ) and electronic chemical potential (μ) were also analyzed. A lower χ and μ indicate a molecule’s increased ability to donate electrons, suggesting antioxidant potential. Specifically, molecules 4j, 4g, 4d, and 4k demonstrated lower values of these descriptors, highlighting their roles as effective electron donors. This aligns with their observed behavior in electron-scavenging mechanisms, which is crucial for understanding charge-transfer reactions.

Density Functional Theory (DFT) calculations were performed to determine the dipole moments of a series of compounds, along with a reference compound, ascorbic acid. As expected, compounds featuring a nitro group at the para position of the phenyl ring (ring E Scheme 2) (4c, 4f, 4i, and 4l) exhibited the highest dipole moments, ranging from 5.538 D to 6.546 D, due to the strong electron-withdrawing nature of the nitro group. Compounds with a chloro substituent at the same position (4b, 4e, 4h, and 4k) displayed lower dipole moments, ranging from 2.026 D to 2.960 D, reflecting the weaker electron-withdrawing effect of chlorine compared to the nitro group. The lowest dipole moments, ranging from 0.738 D to 1.934 D, were observed for compounds lacking a substituent at the para position (4a, 4d, 4g, and 4j). The dipole moment of ascorbic acid was calculated to be 8.697 D. The dipole moments reported in Table 1 confirm the high polarity of these compounds, implying good solubility in polar solvents and possible sensitivity to environmental polarity in their reactivity. The electrophilicity index (ω) further classified the molecules, with values greater than 1.5 eV indicating strong electrophiles (4c, 4f, and 4i). In contrast, Ascorbic acid exhibited the lowest ω value, reinforcing its role as a primary electron donor.

Calculated average polarizabilities (in atomic units, a.u.) followed the order: 4i (360.34) > 4b (355.36) > 4f (349.79) > 4l (345.15) > 4h (345.3) > 4e (343.83) > 4k (334.4) > 4a (332.58) > 4g (329.99) > 4d (325.5) > 4j (314.76) > 4c (312.93) > ascorbic acid (83.65). The investigated compounds generally exhibited higher polarizability values compared to ascorbic acid, suggesting their increased propensity for solubility in polar solvents and their greater ability to induce polarization in neighboring molecules. However, it is crucial to recognize that polarizability is only one factor influencing solubility, and other factors such as hydrogen bonding and specific solute-solvent interactions play significant roles. The polarizability of the investigated compounds was found to be influenced by the nature of the substituent at the para position of the phenyl ring (ring E, Scheme 2). Compounds bearing a nitro group at this position exhibited the highest polarizabilities, followed by those with a chloro substituent. Compounds lacking a substituent at the para position displayed the lowest polarizabilities. This trend can be attributed to the electron-withdrawing nature of the nitro and chloro groups, with the nitro group exerting a stronger effect. The presence of electron-withdrawing groups at the para position reduces electron density in the phenyl ring, making the electron cloud more easily distorted by an external electric field and thus increasing the polarizability.

This result provides a multifaceted view of molecular reactivity, indicating potential for electron donation, acceptance, or participation in charge-transfer processes based on calculated parameters such as, IE, EA, μ, χ, and ω. These parameters suggest that molecules 4d, 4e, 4j, and 4k are promising candidates for electron donation, owing to their lower EA, μ, and χ values, which support their potential antioxidant roles. These conclusions are corroborated by experimental studies conducted by Kotaiah et al. (2012) and have also been validated through computational methods, including HOMO-LUMO analysis.

### 3.3 Local reactivity descriptors

In this section, we selected compounds 4d, 4e, 4j, and 4k for an in-depth examination of their Local Reactivity Descriptors, with detailed data presented in Supplementary Table S3. The choice of these particular compounds was driven by their distinct structural characteristics and the potent antioxidant capabilities they exhibited in previous research conducted by Kotaiah et al. (2012). This prior study highlighted their significant effectiveness, making them ideal candidates for a detailed exploration of their chemical behavior and interactions as antioxidants. This analysis aims to further elucidate the mechanisms underlying their high reactivity and potential therapeutic applications.

Compounds 4d, 4e, 4j, and 4k, showcases varied chemical reactivity across its molecular structure. We’ll analyze this based on the dual descriptor (DD), 𝑃^−^_k_ (for electrophilic attacks), and 𝑃^+^_k_ (for nucleophilic attacks) data provided. The local reactivity descriptors for compounds 4d, 4e, 4j and 4k reveal complex patterns indicating their potential chemical interactions and antioxidant activities, although the description for compound 4k is missing from the document.

Compound 4d exhibits a distinct dual reactivity. Electrophilic attack sites such as C2, C4, C18, and C20 show strong electrophilic characteristics due to high positive values of dual descriptors and for electrophilic attacks, making these regions highly reactive towards nucleophiles. Conversely, nucleophilic sites like N21, C25, and C23, with their negative dual descriptor values and high values for nucleophilic attacks, are likely to attract electrophiles. The antioxidant potential of compound 4d is underscored by its ability to donate electrons, particularly at N21, suggesting a robust capability to neutralize reactive oxygen species and stabilize free radicals, which is crucial for preventing oxidative stress (Table 2).

**TABLE 2:** Local Reactivity Descriptors (Parr Functions) for Compounds 4d, 4e, 4j, and 4k. Values are presented for electrophilic attack (P^−^), nucleophilic attack (P^+^), and dual descriptor (DD) analyses (all values expressed in eV).

4d				4e				4j				4k			
Atom	DD	P-	P+	Atom	DD	P-	P+	Atom	DD	P-	P+	Atom	DD	P-	P+
C1	0.03	−0.36	−0.14	C1	0.04	−0.4	−0.12	C1	−0.01	−0.4	−0.23	C1	0	−0.47	−0.19
C2	0.4	3.97	2.67	C2	0.41	**4.06**	**2.57**	C2	0.19	**4.21**	**3.32**	C2	0.22	4.2	3.23
C3	−0.21	2.01	3.18	C3	−0.26	**1.58**	**3.08**	C3	−0.35	**1.88**	**3.5**	C3	−0.4	1.41	3.42
C4	0.68	2.69	−0.73	C4	0.72	2.73	−0.56	C4	0.67	2.87	−0.84	C4	0.7	2.89	−0.64
N5	0.02	1.58	2.8	N5	−0.01	1.22	**2.63**	N5	−0.11	**1.45**	**3.17**	N5	−0.13	1.1	2.97
N6	−0.17	−1.12	1.35	N6	−0.1	−0.93	1.46	N6	−0.24	−1.05	1.45	N6	−0.16	−0.87	1.58
C7	0.33	2.5	1.42	C7	0.38	2.91	0.85	C7	0.28	2.47	1.65	C7	0.34	2.98	0.97
C8	0.07	0.05	0.85	C8	0.13	0.09	1.18	C8	0.07	0.11	0.93	C8	0.13	0.13	1.28
C9	0.21	2.3	0.43	C9	0.29	2.74	0.32	C9	0.2	2.39	0.49	C9	0.28	2.86	0.37
C10	0.06	−0.83	−0.27	C10	0.08	−0.89	−0.13	C10	0.05	−0.86	−0.3	C10	0.07	−0.91	−0.15
C11	0.19	2.9	2.13	C11	0.27	2.92	1.97	C11	0.16	3.06	2.36	C11	0.25	3.07	2.17
C12	0.18	−0.63	−0.56	Cl12	−0.07	0.19	0.85	C12	0.16	−0.64	−0.62	Cl12	−0.14	0.2	0.92
C13	0.12	1.63	1.04	C13	0.21	−0.59	−0.44	C13	0.09	1.74	1.14	C13	0.2	−0.59	−0.5
O14	0.53	1.31	−0.2	C14	0.2	1.77	0.69	O14	0.53	1.35	−0.23	C14	0.18	1.87	0.78
S15	−0.02	0.8	0.03	O15	0.56	1.36	−0.19	S15	−0.2	0.73	0.21	O15	0.56	1.39	−0.22
C16	−0.19	0.93	0.82	S16	−0.08	0.76	0.04	C16	−0.23	0.55	0.96	S16	−0.25	0.71	0.21
N17	−0.3	−0.13	1.56	C17	−0.17	0.72	0.89	N17	−0.31	−0.12	1.6	C17	−0.21	0.5	0.98
C18	0.53	4.09	0.65	N18	−0.34	−0.23	1.54	C18	0.4	4.06	0.94	N18	−0.35	−0.23	1.58
N19	−0.08	−1.55	0.27	C19	0.49	3.86	0.59	N19	−0.11	−1.38	0.11	C19	0.36	3.81	0.87
C20	0.62	4.22	−0.07	N20	−0.08	−1.44	0.26	C20	0.48	3.88	0.31	N20	−0.11	−1.3	0.12
N21	−1.04	0.46	4.74	C21	0.56	3.47	0.11	N21	**−0.87**	**0.4**	**3.87**	C21	0.43	3.28	0.43
C22	−0.3	−0.15	0.74	N22	**−1.01**	**0.47**	**4.61**	C22	−0.11	−0.71	0.3	**N22**	**−0.85**	**0.4**	**3.77**
C23	−0.32	0.47	0.96	C23	−0.31	−0.16	0.71	C23	−0.07	0.99	0.27	C23	−0.13	−0.47	0.23
C24	−0.17	0.31	−0.56	C24	−0.32	0.25	0.89	C24	−0.15	0.16	−0.06	C24	−0.1	0.69	0.24
C25	−0.43	0.36	2.16	C25	−0.19	0.26	−0.53	C25	−0.18	0.47	1.1	C25	−0.16	0.09	−0.09
F26	−0.37	0	0.3	C26	−0.43	0.32	**2.1**	F26	−0.19	0.01	0.16	C26	−0.21	0.42	1.1
C27	−0.26	−0.25	−0.53	F27	−0.37	0	0.29	C27	−0.09	0	−0.38	F27	−0.21	0.01	0.15
C28	−0.31	0.22	1.02	C28	−0.26	−0.18	−0.5	C28	−0.21	−0.03	0.79	C28	−0.12	−0.09	−0.36
C29	−0.05	−0.6	1.11	C29	−0.32	0.29	1.01	F29	−0.15	−0.01	0.08	C29	−0.21	0.05	0.82
				C30	−0.07	−0.15	0.88	C30	−0.09	−0.57	0.91	F30	−0.15	−0.01	0.08
												C31	−0.11	−0.25	0.73

The bold values provided in Table 2 represent the most significant or important values among the data presented in those tables.

Compound 4e also presents a sophisticated behavior in chemical reactivity. Electrophilic sites such as C2, C11, C19, and C21 are characterized by high values for electrophilic attacks and positive dual descriptors, indicating their readiness to engage with nucleophiles. N22 stands out as a primary nucleophilic site due to its high potential for electron donation, marked by the highest value for nucleophilic attacks and a strongly negative dual descriptor. Other sites like C26 and N18 also display nucleophilic tendencies but to a lesser extent. The antioxidant actions of compound 4e are likely facilitated by electron donation and radical scavenging at these nucleophilic sites, particularly N22, which might play a pivotal role in neutralizing reactive oxygen species (Table 2).

Compound 4j, as analyzed, shows that C2 and C18 are significant electrophilic sites with very high values for electrophilic attacks, suggesting their strong capability to accept electrons. N21 emerges as a primary nucleophilic site, with a high value for nucleophilic attacks and a negative dual descriptor, indicating its effectiveness in donating electrons. The antioxidant potential of compound 4j is emphasized by N21’s capacity to donate electrons, potentially contributing significantly to neutralizing reactive oxygen species, alongside other nucleophilic sites like C3 and N5 that might engage in radical scavenging (Table 2).

Compound 4k, exhibits a wide range of chemical reactivity crucial for understanding its behavior in chemical reactions and its potential as an antioxidant. The theoretical analysis of its reactivity descriptors indicated that carbon atoms C2 and C19 are highly receptive to electrophilic attacks due to their strong nucleophilic properties, and N22 is a significant site for electron donation because of its exceptional nucleophilic reactivity and a strong negative dual descriptor. These properties suggested strong antioxidant capabilities, particularly through electron donation to neutralize reactive oxygen species. Experimental studies conducted by Kotaiah et al. (2012) have validated these theoretical predictions, confirming that the reactivity observed at sites like N22, C3, and N5 enables compound 4k to act effectively as an antioxidant. The agreement between the theoretical analysis and experimental results highlights the molecule’s potential for therapeutic applications targeting oxidative stress, substantiating its suitability for further pharmacological exploration (Lončar et al., 2021).

### 3.4 Frontier molecular orbitals (FMOs)

The Frontier Molecular Orbitals (FMOs), specifically the Highest Occupied Molecular Orbital (HOMO) and the Lowest Unoccupied Molecular Orbital (LUMO), are crucial for predicting molecular reactivity and assessing the antioxidant properties of compounds. The energy associated with the HOMO (E_HOMO_) illustrates a molecule’s electron-donating capability, which is a thermodynamically favorable process. Conversely, the LUMO energy (E_LUMO_) reflects the molecule’s ability to accept electrons. A lower E_HOMO_ suggests reduced electron-donating propensity, and the localization of HOMO also determines potential sites for free radical susceptibility.

**FIGURE 2:**
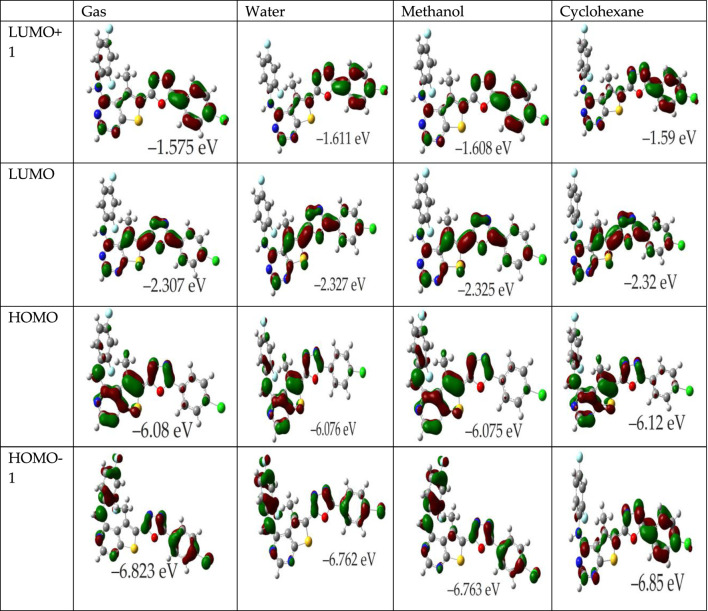
Influence of solvent environment on the HOMO and LUMO orbitals of molecule 4k. Depicted are the orbitals in gas phase, water, methanol, and cyclohexane.

For instance, in various solvents (gas, water, methanol, cyclohexane), the FMOs of compound 4k and in the gas phase for OTP derivatives labeled 4a-4j and 4l are depicted in Figure 2; Supplementary Figure S2, respectively. These illustrations indicate that the π-like FMOs, indicative of potential antioxidant properties, are distributed extensively across the molecules. For the OTP molecules (4a-4l), the HOMO is primarily localized on the thieno[2,3-d]pyrimidin-4-amine and oxadiazole rings, highlighting the NH groups as likely targets for free radical attacks, potentially leading to electron or hydrogen abstraction. The LUMO, however, displays significant contributions from the carbons in the phenyl and oxadiazole rings, with the NH groups showing no involvement. Particularly, the distribution of both HOMO and LUMO in compound 4k across all investigated media is extensive. The HOMO orbitals prominently cover the thieno[2,3-d]pyrimidin-4-amine and oxadiazole rings, which are implicated in the antioxidant activity. Furthermore, the LUMO+1 is primarily associated with the chlorophenyl and oxadiazole rings, while the LUMO is concentrated over the thieno[2,3-d]pyrimidine ring. This configuration consistency across different phases suggests a stable FMO pattern that correlates with theoretical predictions where E_HOMO_ is an indicator of a molecule’s free radical scavenging ability. The energy gap between the HOMO and LUMO is a pivotal factor in determining a compound’s chemical activity. A smaller gap indicates increased molecular softness (reactivity), which facilitates easier electron donation to acceptors, whereas a larger gap implies molecular hardness (stability). The global reactivity parameters derived from E_HOMO_ and E_LUMO_, as presented in the referenced table, are critical for understanding these interactions. Moreover, the LUMO’s distribution reveals significant contributions from the carbons of the phenyl and oxadiazole rings, suggesting regions of interest for interactions with nucleophiles. Compounds with a narrower HOMO-LUMO gap are more polarizable, facilitating a significant degree of intermolecular charge transfer between electron donors and acceptors, potentially influencing the molecule’s biological activity (El Bahnasawy et al., 1993; Kotaiah et al., 2012). According to results in Table 1, molecules 4c, 4f, and 4i exhibit a lower HOMO-LUMO gap, indicating high polarizability and potential biological activity, similar to that observed for molecules 4k, 4j, 4d, and 4e. The presence of amino aromatic groups and aromatic rings with nitro, chloro, and fluoro substitution groups provides a solid basis for reactivity. Molecules containing nitro groups generally exhibit lower energy gaps due to the strong electron-withdrawing nature of the nitro group, which stabilizes the LUMO and destabilizes the HOMO. However, the IC50 values of these molecules often indicate lesser activity. Lower IC50 values correspond to higher potential antioxidant activity. Therefore, despite favorable electronic properties suggested by low energy gaps, the practical radical scavenging activity of nitro-substituted compounds may be lower compared to other derivatives (Al-Sehemi and Irfan, 2017; Dimić et al., 2017; Lončar et al., 2021).

### 3.5 Molecular electrostatic potential: electrostatic potential isosurface

**FIGURE 3:**
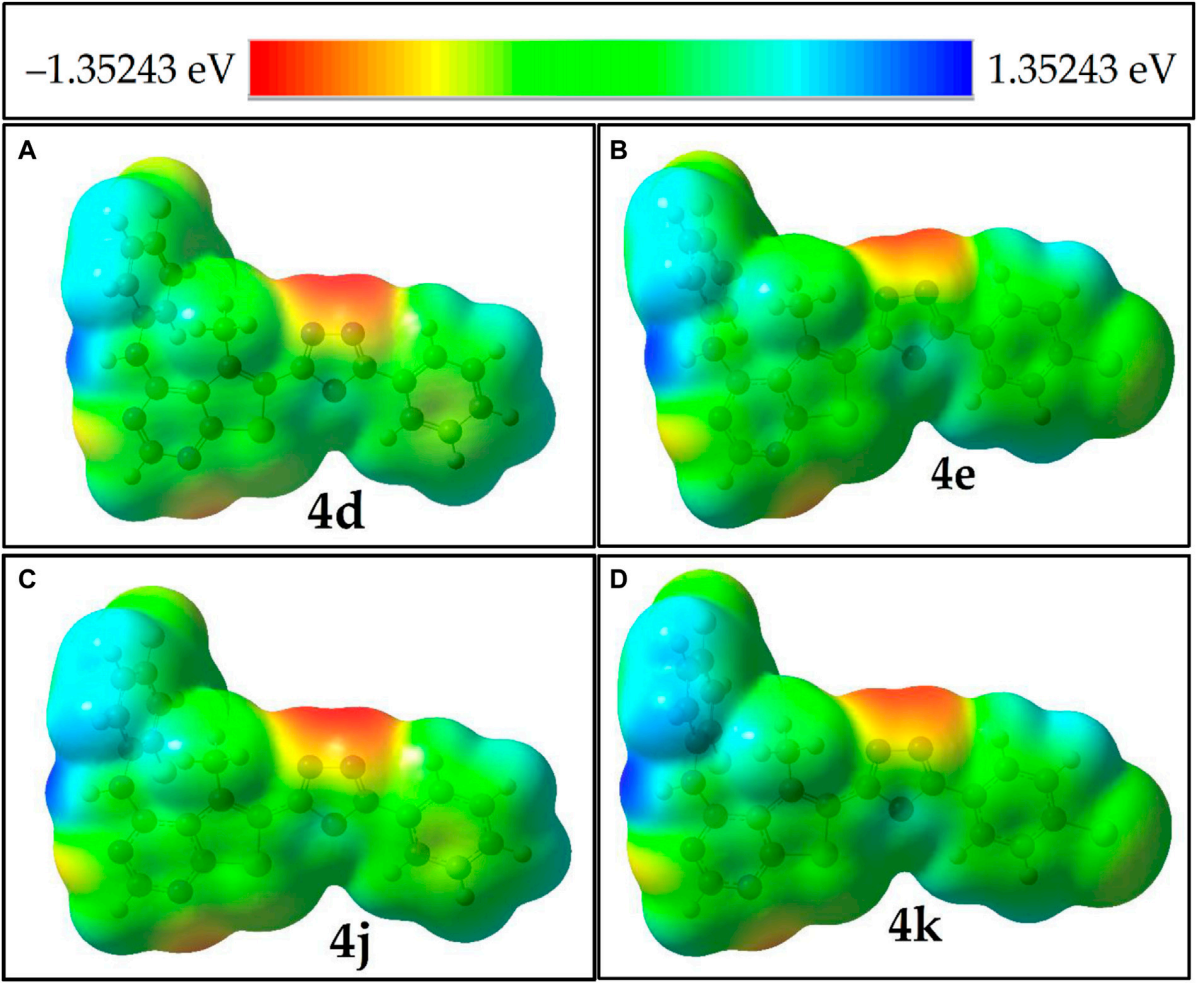
MEP isosurfaces of molecules panel **(A)** compound 4d, **(B)** compound 4e, **(C)** compound 4j and **(D)** compound 4k, that highly potent in experimental results.

The molecular electrostatic potential (MEP) serves as a crucial descriptor for assessing the reactivity of molecular systems. The three-dimensional MEP surface provides insights into the locations, shapes, and sizes of regions with positive, negative, and neutral electrostatic potentials. This information enhances our understanding of the physicochemical properties of the system and their relationship with molecular structure, influencing reactivity towards electrophilic and nucleophilic attacks. As illustrated in Figure 3; Supplementary Figure S4, regions of maximum negative electronic potential, depicted in red, are the preferred sites for electrophilic attacks. Conversely, areas of positive electrostatic potential, shown in blue, attract charged molecules or radicals. Analysis of the MEP data from Figure 3; Supplementary Figures S3, S4 reveals that high electrostatic potential regions in neutral molecules, radical species (RN and RNH+) molecules are predominantly located near the nitrogens of the oxadiazole and pyrimidine rings.

Meanwhile, in neutral molecules and radical species (RNH^+^), a relatively low electropositive potential is primarily observed on the H1 atom of the amine group. In contrast, radical RN molecules exhibit electropositive potentials at all aromatic hydrogen atoms. Comparative analysis of the blue color codes representing positive potentials (+1.092, +4.104, +0.838, and +4.05 eV) for molecule 4k (neutral molecule, radical RNH+, RN, and anionic molecule, respectively) indicates that molecule 4k, after donating a radical electron, shows increased polarizability (α) and dipole moment. This is evidenced by the MEP isosurfaces, which appear blue around the H1 atom of the amine group, indicating it as a favorable site for hydrogen radical donation.

Upon donating this hydrogen, the radical molecule (RN) adopts a more planar configuration (see Figure 1; Supplementary Figure S4). This change is accompanied by an increased dipole moment and a redistribution of electropositive potential (blue) around the aromatic hydrogens, albeit with lower values.

Additionally, a comparative analysis of the blue color codes representing positive potentials—(+1.51, +1.452, +1.421, and +1.412 eV) for molecules 4i, 4c, 4f, and 4h respectively—suggests that the H atom of the amine group in molecule 4i is the most susceptible site for nucleophilic attacks. The closely matched values for the H atoms of the amine groups in molecules 4c, 4f, and 4h indicate these sites are also favourable for nucleophilic interactions. The intense electrostatic potential near the nitrogen atoms of the amine groups further identifies these regions as effective radical traps.

### 3.6 Thermochemical parameters characterizing antioxidant capacity

Thermodynamic analysis of the antiradical mechanisms was firstly carried out in the gas phase in order to evaluate the suitability of OTP as an antiradical agent toward representative ROS and RNS. In the absence of the solvent, only formal hydrogen transfer (FHT) and radical adduct.

#### 3.6.1 Antioxidant capacity via hydrogen atom transfer–bond dissociation enthalpy

The bond dissociation enthalpy (BDE) serves as a crucial thermodynamic parameter for describing the hydrogen atom transfer (HAT) mechanism in antioxidants. This process involves the transfer of a hydrogen atom from the amine group or carbon ring of the antioxidant compound to a free radical. Typically, the N–H or C–H bond with the lowest BDE is more readily abstracted, indicating higher antiradical (antioxidant) activity. Consequently, the computed BDE values for N–H and C–H bonds in various ring structures are reported in Table 3. Notably, the N–H bonds in molecules 4d, 4g, 4h, and 4e exhibit the lowest BDE values, correlating with enhanced radical scavenging reactivity, as supported by the experimental findings of Kotaiah et al. (2012) Similarly, the C–H bonds in molecules 4d, 4j, 4e, and 4k also show low BDE values, again indicating high radical scavenging reactivity, consistent with the experimental results reported by Kotaiah et al. (2012) formation (RAF) mechanisms are thermodynamically favorable as demonstrated in previous studies (Galano et al., 2016; Wang et al., 2020; Abuelizz et al., 2021a; Abuelizz et al., 2021b).

**TABLE 3:** BDE, IP, PDE, PA, and ETE for N–H/C–H Bonds at the B3LYP/6-311g(d,p) Level of Theory in the Gas Phase (all values expressed in kcal·mol⁻^1^).

Molecules	Atoms	BDE	IP	PDE	PA	ETE	SET-PT	SPLET
4a	N21–H	78.15	167.13	228.86	321.93	73.33	395.99	395.26
	C18–H	106.37	167.13	257.08	385.74	37.73	424.21	423.48
4b	N22–H	78.22	168.42	227.65	320.26	75.08	396.07	395.33
	C19–H	106.4	168.42	255.83	384.08	39.43	424.25	423.51
4c	N25–H	78.38	173.27	222.96	316.44	79.05	396.23	395.49
	C22–H	106.46	173.27	251.04	379.69	43.88	424.31	423.57
4d	N21–H	77.67	169.05	226.46	335.2	59.58	395.51	394.78
	C18–H	106.27	169.05	255.06	387.2	36.17	424.11	423.38
4e	N22–H	77.75	170.3	225.29	322.37	72.49	395.59	394.86
	C19–H	106.31	170.3	253.85	385.49	37.93	424.15	423.42
4f	N22–H	77.9	175.21	220.53	318.34	76.67	395.74	395
	C12–H	107.93	175.21	250.57	385.29	39.75	425.77	425.04
4g	N21–H	77.55	171.15	224.24	319.54	75.11	395.39	394.65
	C18–H	106.45	171.15	253.15	385.31	38.25	424.3	423.56
4h	N22–H	77.68	172.35	223.17	317.92	76.87	395.52	394.79
	C19–H	106.5	172.35	251.99	383.63	39.98	424.34	423.61
4i	N16–H	77.91	177.44	218.32	314.2	80.82	395.75	395.02
	C26–H	106.56	177.44	246.97	379.27	44.4	424.41	423.67
4j	N21–H	79.05	169.67	227.23	322.68	73.49	396.9	396.16
	C18–H	106.3	169.67	254.47	389.29	34.12	424.14	423.41
4k	N22–H	79.14	167.82	229.16	320.94	75.31	396.98	396.25
	C19–H	106.34	167.82	256.37	387.5	35.96	424.19	423.45
4l	N16–H	79.33	176.07	221.11	317.01	79.43	397.18	396.44
	C18–H	106.42	176.07	248.19	382.56	40.97	424.26	423.53
Ascorbic acid	O1–H	61.71	196.57	182.99	320.03	58.8	379.56	378.82
	O12–H	74.27	196.57	195.55	333.99	57.39	392.11	391.38

Substituents play a critical role in determining the antioxidant efficacy of phenolic compounds. Electron-donating groups, such as–OH and–NH2, generally enhance antioxidant activity by stabilizing the arylamine free radical through resonance and inductive effects, which lower the bond dissociation enthalpy (BDE) and facilitate hydrogen atom transfer (HAT). Conversely, electron-withdrawing groups, such as–NO2 and–F, can have a dual effect: while they stabilize the LUMO and reduce the energy gap, potentially enhancing reactivity, they can also increase the BDE, making hydrogen atom donation less favourable, thus affecting the overall antioxidant capacity negatively. The position of these substituents also matters significantly; for instance, para-substituted groups tend to have a more substantial impact on the electronic distribution and radical stability. Additionally, the ability of substituents to form intramolecular hydrogen bonds can further stabilize the arylamine free radicals, enhancing antioxidant activity. These nuanced effects underscore the complex interplay between substituent type, position, and the resulting electronic and steric environment, as highlighted in the referenced studies.

In all evaluated cases, the abstraction of a hydrogen atom from an N–H bond typically results in a lower BDE compared to the abstraction from a C–H bond. This variation in BDE values can be attributed to the influence of substituents on the adjacent phenyl group, particularly at the para position. The presence of electron-donating groups, such as fluoro, on the para position of the phenyl rings significantly enhances their effectiveness, surpassing even that of ascorbic acid, which is used as a reference antioxidant. Across molecules 4d to 4i, the BDE values are relatively similar, demonstrating the significant effect of electron-donating groups like fluoro at the para position of the phenyl rings on hydrogen atom abstraction. The calculated BDEs for two hydrogens in a single molecule are notably similar, and all values closely approximate those of ascorbic acid. This similarity suggests that the investigated systems possess hydrogen-donating abilities comparable to that of ascorbic acid.

#### 3.6.2 Antioxidant mechanisms: single-electron transfer followed by proton transfer (SET-PT)

Apart from the hydrogen atom transfer (HAT) mechanism, antioxidants may also operate through the single-electron transfer followed by proton transfer (SET-PT) pathway. In this mechanism, an electron is first transferred from the antioxidant to the free radical, forming a radical cation. This radical cation subsequently deprotonates. The adiabatic ionization potential (IP) and the proton dissociation enthalpy (PDE) are critical parameters for evaluating the feasibility of this mechanism. The calculated IPs and PDEs are presented in Table 3.

The IP required for the initial electron transfer step is generally lower than that needed for the subsequent proton transfer, making the latter more likely to be the rate-limiting step, especially in polar solvents. Typically, lower IPs facilitate easier electron transfer between free radicals and antioxidants. By comparison, molecules such as 4a and 4k exhibit IPs close to each other, with the sequence being 4a ≤ 4k < 4b < 4d < 4j < 4e < 4g < 4h < 4c < 4f < 4l < 4i < ascorbic acid, all of which are higher than the BDEs obtained from their respective radical sites. This sequence suggests that molecule 4k is among the most active antioxidants, with all examined IPs being lower than that of ascorbic acid, indicating potentially higher activity in this mechanism.

In the second step of the SET-PT mechanism, PDE measures the ease of deprotonation of the formed radical cations. The lowest PDE values are observed for the OH group in ascorbic acid, followed by 4a and 4k, indicating a higher propensity for deprotonation. The SET-PT values for NH groups across all investigated molecules are relatively uniform, demonstrating the significant influence of electron-donating groups such as difluoro, fluoro, and chloro-fluoro at the para position of the phenyl rings on hydrogen atom abstraction.

However, the order of molecules based on SET-PT values for CH groups is as follows: ascorbic acid < 4d < 4j < 4e < 4k < 4a < 4b < 4l < 4g < 4c < 4h < 4i < 4f. Despite slight variations, all investigated molecules exhibit relatively similar values.

Although the trends in IP differ from those of BDE, the PDE values and the combined energies of the two steps (IP + PDE) follow a similar trend to that of BDE energies. Ascorbic acid shows lower energies compared to other molecules, confirming higher activities, particularly in comparison to 1,3,4-oxadiazole tagged thieno[2,3-d]pyrimidine derivatives 4a-4l, as supported by experimental evidence. Furthermore, the PDE values for N–H1 are consistently lower than those for C–H2 in all the studied compounds (4a–4l), indicating that the hydroxyl group loses its proton more easily from the radical cation. This finding aligns with experimental evidence reported by Kotaiah et al. (2012).

However, despite these insights, the energies required for the entire SET-PT reaction are significantly higher than those for the HAT mechanism, suggesting that SET-PT may not be the preferred pathway for these marine natural compounds in the gas phase (Raghavan and Steenken, 1980; Roder et al., 1999).

#### 3.6.3 Antioxidant capacity via sequential proton loss electron transfer (SPLET)–proton affinity (PA) and electron transfer enthalpy (ETE)

Proton affinity (PA) and electron transfer enthalpy (ETE) are two crucial physicochemical parameters employed to assess antioxidant activity through the SPLET mechanism. This mechanism generally involves two key steps: a proton loss process, indicated by the PA value, followed by an electron transfer, represented by the ETE value. Lower PA values are indicative of higher antioxidant potential. Initially, PAs for all potential bond dissociations were estimated using the semi-empirical PM6 method to identify the most favorable deprotonation sites (Supplementary Table S1). These values were then refined using the higher-level B3LYP/6-311G(d,p) computational method, and the results are presented in Table 4.

**TABLE 4:** BDE, IP, PDE, PA, and ETE Values for N–H/C–H Bonds of Compound 4k at the B3LYP/6-311g(d,p) Level of Theory in Methanol, Water, and Cyclohexane (all values expressed in kcal·mol⁻^1^).

Solvant	Atoms	BDE	IP	PDE	PA	ETE	SET-PT	SPLET
4k								
Water	N22–H	80.37	115.44	160.31	30.59	89.17	275.75	119.76
	C19–H	105.87	115.44	185.81	84.01	61.25	301.25	145.26
Methanol	N22–H	80.34	111.01	157.78	34.27	83.42	268.79	117.69
	C19–H	105.89	111.01	183.33	88.07	55.17	294.35	143.24
Cyclohexane	N22–H	79.63	151.61	192.5	119.77	91.9	344.11	211.67
	C19–H	106.19	151.61	219.06	181.28	56.95	370.67	238.23
Ascorbic acid								
Water	O1–H	72.96	127.24	133.88	277.03	84.4	261.12	361.43
	O12–H	105.87	65.74	141.1	32.18	80.17	206.84	112.35
Methanol	O1–H	73.02	123.23	130.82	277.72	78.52	254.05	356.23
	O12–H	105.89	65.59	138.25	35.95	74.42	203.84	110.37
Cyclohexane	O1–H	73.8	172.64	154.87	296.9	82.06	327.51	378.96
	O12–H	106.19	63.03	165.65	126.09	79.76	228.68	205.85

It is typically observed that heterolytic cleavage preferentially occurs at N–H and C–H positions near C=C double bonds. The molecule 4k emerged as the most potent antioxidant based on the SPLET mechanism, with PA values for N–H and C–H in the gas phase being 320.94 kcal/mol and 387.5 kcal/mol, respectively. In polar solvents, a significant reduction in PA values was noted (water: 30.59 kcal/mol for N–H, 84.01 kcal/mol for C–H; methanol: 34.27 kcal/mol for N–H, 88.07 kcal/mol for C–H), compared to those in nonpolar solvent cyclohexane (119.77 kcal/mol for N–H, 181.28 kcal/mol for C–H) (Table 3).

In the case of ascorbic acid, its PA in the gas phase is 320.03 kcal/mol, with markedly lower values in water (32.18 kcal/mol) and methanol (35.95 kcal/mol). These observations underscore the significant influence of solvation enthalpy on proton affinity, aligning well with findings from prior research (Table 3).

Concerning the electron transfer enthalpy (ETE), which quantifies the electron-donating capacity of the anion formed in the initial step of the SPLET mechanism, it was observed that ETE values in the gas phase are substantially lower than the corresponding PA values. For instance, the ETE for 4k in the gas phase is 75.31 kcal/mol for N–H and 35.96 kcal/mol for C–H, whereas the PA is considerably higher (320.94 kcal/mol for N–H and 387.5 kcal/mol for C–H). This discrepancy indicates that electron transfer from the anionic form is more energetically favorable than from the neutral form, which is consistent with previous studies (Vo et al., 2018).

Among the compounds evaluated, 4k, 4j, 4d, and 4e have demonstrated substantial efficiency as antioxidants through both HAT and SPLET mechanisms.

From the experimental results, compounds 4j and 4k demonstrated the lowest IC50 values, indicating they possess the highest antioxidant activity among the tested derivatives. These compounds feature difluoro substituents in the para and meta positions of the arylamino group, which significantly contribute to their enhanced activity. In contrast, compounds 4c, 4f, 4i, and 4l, which contain a nitro group in the para position of the phenyl group, show higher IC50 values, reflecting reduced antioxidant activity. Compounds 4d and 4e, featuring a para fluoro substituent, exhibit moderate antioxidant activity.

The theoretical calculations provide detailed insights into the reactivity and stability of these derivatives. Specifically, the BDE values for the N-H bonds show that compounds with lower BDE values generally exhibit higher antioxidant activity. For instance, compound 4k has an N-H BDE of 79.14 kcal/mol, while compound 4j has a BDE of 79.05 kcal/mol. These values are higher compared to other derivatives, suggesting that stronger N-H bonds correlate with better antioxidant activity. In contrast, compounds like 4d (77.67 kcal/mol) and 4e (77.75 kcal/mol) have lower BDE values, aligning with their moderate IC50 values.

The SET-PT and SPLET values further illustrate the antioxidant potential. Compounds 4j and 4k exhibit higher SET-PT values (396.90 kcal/mol and 396.98 kcal/mol, respectively) and SPLET values (396.16 kcal/mol and 396.25 kcal/mol, respectively), indicating a greater ability to undergo electron transfer and proton loss processes, which are essential for antioxidant activity. On the other hand, compounds 4c, 4f, 4i, and 4l with nitro substituents have relatively lower SET-PT and SPLET values, which correlates with their higher IC50 values and thus lower antioxidant activity.

Comparing these results with the reference compound, ascorbic acid, which has significantly lower BDE (61.71 kcal/mol and 74.27 kcal/mol for different O-H bonds), SET-PT (379.56 kcal/mol and 392.11 kcal/mol), and SPLET (378.82 kcal/mol and 391.38 kcal/mol) values, confirms that lower values in these parameters are indicative of stronger antioxidant properties. Ascorbic acid’s superior antioxidant activity is reflected in its lowest IC50 value.

## 4 Conclusion

In this study, we employed density functional theory (DFT) to investigate the antioxidant mechanisms of 1,3,4-oxadiazol-2-ylthieno[2,3-d]pyrimidin-4-amine derivatives (OTP). Our computational approach analyzed various thermochemical parameters, including bond dissociation enthalpy (BDE), ionization potential (IP), proton dissociation enthalpy (PDE), and electron transfer enthalpy (ETE), to elucidate the antioxidant activity of these compounds. The study was conducted in both gas phase and solvent environments (methanol and water) to assess the influence of solvent on the antioxidant properties.

In this study, structure optimization and the effects of substituent groups on the antioxidant activity of 1,3,4-oxadiazol-2-ylthieno[2,3-d]pyrimidin-4-amine derivatives were thoroughly investigated using density functional theory (DFT). The optimized structures revealed that the presence of electron-donating groups, such as amine, significantly enhances antioxidant properties by lowering bond dissociation enthalpy (BDE) and ionization potential (IP). Specifically, compounds with these substituents demonstrated easier hydrogen atom transfer (HAT) and single electron transfer (SET) mechanisms. These findings highlight the crucial role of substituent groups in modulating the reactivity and stability of these derivatives, paving the way for the design of more effective synthetic antioxidants.

The findings revealed that the presence of electron-donating groups, such as amine, on the phenyl rings significantly lowers the bond dissociation enthalpy (BDE), thereby enhancing the antioxidant activity. For instance, the BDE for the N-H bond in compound 4a was found to be 78.15 kcal/mol in the gas phase, whereas in compound 4k, it was slightly different at 79.14 kcal/mol, indicating a similar hydrogen atom transfer (HAT) process. The Ionization Potential (IP) values were determined to assess the ease of electron donation, with compound 4a calculated to have an IP of 7.25 eV. In comparison, compound 4k had an IP of 7.27 eV, suggesting that the latter has a similar propensity for single electron transfer (SET).

Additionally, the PDE values were computed to understand the proton transfer capabilities. Compound 4a exhibited a PDE of 228.86 kcal/mol, whereas for compound 4k, it was 229.16 kcal/mol, supporting the enhanced antioxidant mechanism via sequential proton loss electron transfer (SPLET). The ETE values highlighted the electron transfer abilities of the derivatives, with compound 4a showing an ETE of 73.33 kcal/mol, while compound 4k demonstrated a lower ETE of 75.31 kcal/mol, indicating more efficient electron transfer in the latter.

The global and local reactivity descriptors were also analyzed. The HOMO-LUMO energy gaps were computed to predict the stability and reactivity, with compound 4a having a HOMO-LUMO gap of 6.369 eV, while compound 4k had a gap of 6.398 eV, indicating that compound 4k has similar reactivity. Fukui functions were analyzed to identify the reactive sites on the molecules, with compound 4k showing high *P*+ values similar to or less than those at the nitrogen atoms of the aniline group, indicating these sites as more nucleophilic. Furthermore, the calculations conducted in solvent environments showed a decrease in the BDE, IP, and PDE values compared to the gas phase, demonstrating that the antioxidant activity is slightly enhanced in non-polar solvents like cyclohexane. For example, the BDE for the N-H bond in compound 4k was reduced to 79.63 kcal/mol in water.

The SAR analysis suggests that the antioxidant activity of these derivatives is influenced significantly by the nature and position of the substituent groups. Compounds 4j and 4k, with difluoro groups, show superior antioxidant activity due to their higher BDE, SET-PT, and SPLET values, indicating strong bond stability and efficient electron transfer/proton loss mechanisms. In contrast, compounds with nitro groups in the para position of the phenyl ring exhibit higher IC50 values and lower theoretical parameter values, reflecting weaker antioxidant properties. This comprehensive analysis highlights the crucial role of substituent groups in modulating the antioxidant efficacy of these derivatives, providing valuable insights for future design and optimization of synthetic antioxidants.

## Data Availability

Publicly available datasets were analyzed in this study. This data can be found here: https://doi.org/10.1016/j.ejmech.2012.10.007.

## References

[B1] Abuelizz H. A. Taie H. A. A. Bakheit A. H. Marzouk M. Abdellatif M. M. Al-Salahi R. (2021b). Biological evaluation of 4-(1H-triazol-1-yl)benzoic acid hybrids as antioxidant agents: *in vitro* screening and DFT study. Appl. Sci. 11 (24), 11642. 10.3390/app112411642

[B2] Abuelizz H. A. Taie H. A. A. Bakheit A. H. Mostafa G. A. E. Marzouk M. Rashid H. (2021a). Investigation of 4-hydrazinobenzoic acid derivatives for their antioxidant activity: *in vitro* screening and DFT study. ACS Omega 6 (47), 31993–32004. 10.1021/acsomega.1c04772 34870022 PMC8638017

[B3] Alecu I. Zheng J. Zhao Y. Truhlar D. G. (2010). Computational thermochemistry: scale factor databases and scale factors for vibrational frequencies obtained from electronic model chemistries. J. Chem. theory Comput. 6 (9), 2872–2887. 10.1021/ct100326h 26616087

[B4] Al-Sehemi A. G. Irfan A. (2017). Effect of donor and acceptor groups on radical scavenging activity of phenol by density functional theory. Arabian J. Chem. 10, S1703–S1710. 10.1016/j.arabjc.2013.06.019

[B5] Ayers P. W. Morell C. De Proft F. Geerlings P. (2007). Understanding the Woodward–Hoffmann rules by using changes in electron density. Chemistry–A Eur. J. 13 (29), 8240–8247. 10.1002/chem.200700365 17639522

[B6] Bakheit A. H. Al-Salahi R. Al-Majed A. A. (2022a). Thermodynamic and computational (DFT) study of non-covalent interaction mechanisms of charge transfer complex of linagliptin with 2,3-Dichloro-5,6-dicyano-1,4-benzoquinone (DDQ) and chloranilic acid (CHA). Molecules 27 (19), 6320. 10.3390/molecules27196320 36234857 PMC9572772

[B7] Bakheit A. H. Al-Salahi R. Ghabbour H. A. Ali E. A. AlRuqi O. S. Mostafa G. A. E. (2023). Synthesis, X-ray crystal structure, and computational characterization of tetraphenylborate, 3-(5H-Dibenzo[a,d] cyclohepten-5-ylidene)-N, N-Dimethyl-1-propanamine. Crystals 13 (7), 1088. 10.3390/cryst13071088

[B8] Bakheit A. H. Ghabbour H. A. Hussain H. Al-Salahi R. Ali E. A. Mostafa G. A. (2022b). Synthesis and computational and X-ray structure of 2, 3, 5-triphenyl tetrazolium, 5-Ethyl-5-phenylbarbituric acid salt. Crystals 12 (12), 1706. 10.3390/cryst12121706

[B9] Barone V. Cossi M. (1998). Quantum calculation of molecular energies and energy gradients in solution by a conductor solvent model. J. Phys. Chem. A 102 (11), 1995–2001. 10.1021/jp9716997

[B10] Barone V. Cossi M. Tomasi J. (1997). A new definition of cavities for the computation of solvation free energies by the polarizable continuum model. J. Chem. Phys. 107 (8), 3210–3221. 10.1063/1.474671

[B11] Bassetto M. Leyssen P. Neyts J. Yerukhimovich M. M. Frick D. N. Brancale A. (2016). Computer-aided identification, synthesis and evaluation of substituted thienopyrimidines as novel inhibitors of HCV replication. Eur. J. Med. Chem. 123, 31–47. 10.1016/j.ejmech.2016.07.035 27474921

[B12] Becke A. D. (1988). Density-functional exchange-energy approximation with correct asymptotic behavior. Phys. Rev. A 38 (6), 3098–3100. 10.1103/physreva.38.3098 9900728

[B13] Bozorov K. Zhao J.-Y. Elmuradov B. Pataer A. Aisa H. A. (2015). Recent developments regarding the use of thieno[2,3-d]pyrimidin-4-one derivatives in medicinal chemistry, with a focus on their synthesis and anticancer properties. Eur. J. Med. Chem. 102, 552–573. 10.1016/j.ejmech.2015.08.018 26312434

[B14] Bugge S. Buene A. F. Jurisch-Yaksi N. Moen I. U. Skjønsfjell E. M. Sundby E. (2016). Extended structure–activity study of thienopyrimidine-based EGFR inhibitors with evaluation of drug-like properties. Eur. J. Med. Chem. 107, 255–274. 10.1016/j.ejmech.2015.11.012 26599532

[B15] Carpenter J. Weinhold F. (1988). Torsion-vibration interactions in hydrogen peroxide. 2. Natural bond orbital analysis. J. Phys. Chem. 92 (15), 4306–4313. 10.1021/j100326a013

[B16] Chamorro E. Pérez P. Domingo L. R. (2013). On the nature of Parr functions to predict the most reactive sites along organic polar reactions. Chem. Phys. Lett. 582, 141–143. 10.1016/j.cplett.2013.07.020

[B17] Cossi M. Barone V. Cammi R. Tomasi J. (1996). *Ab initio* study of solvated molecules: a new implementation of the polarizable continuum model. Chem. Phys. Lett. 255 (4-6), 327–335. 10.1016/0009-2614(96)00349-1

[B18] Devlin F. Finley J. Stephens P. Frisch M. (1995). *Ab initio* calculation of vibrational absorption and circular dichroism spectra using density functional force fields: a comparison of local, nonlocal, and hybrid density functionals. J. Phys. Chem. 99 (46), 16883–16902. 10.1021/j100046a014

[B19] Dimić D. Milenković D. Marković J. D. Marković Z. (2017). Antiradical activity of catecholamines and metabolites of dopamine: theoretical and experimental study. Phys. Chem. Chem. Phys. 19 (20), 12970–12980. 10.1039/c7cp01716b 28480927

[B20] Domingo L. R. Pérez P. Sáez J. A. (2013). Understanding the local reactivity in polar organic reactions through electrophilic and nucleophilic Parr functions. RSC Adv. 3 (5), 1486–1494. 10.1039/c2ra22886f

[B21] El Bahnasawy R. El Shereafy E. Kashar T. (1993). Thermal and temperature dependence of electrical conductivity studies on Zn, Cd and Hg hydrazone complexes. J. Therm. analysis 39, 65–74. 10.1007/bf02235447

[B22] El-Sayed W. A. Ali O. M. Zyada R. Mohamed A. A. Abdel-Rahman A. (2012). Synthesis and antimicrobial activity of new substituted thienopyrimidines, their tetrazolyl and sugar derivatives. Acta Pol. Pharm. 69 (3), 439–447. 22594258

[B23] Farrokhnia M. (2020). Density functional theory studies on the antioxidant mechanism and electronic properties of some bioactive marine meroterpenoids: sargahydroquionic acid and sargachromanol. ACS Omega 5 (32), 20382–20390. 10.1021/acsomega.0c02354 32832791 PMC7439385

[B24] Frisch M. J. T. Schlegel H. B. Scuseria G. E. Robb M. A. Cheeseman J. R. Scalmani G. (2009). Gaussian 09, revision D.01. Wallingford, CT, USA: Gaussian Inc., 150–166.

[B25] Galano A. Mazzone G. Alvarez-Diduk R. Marino T. Alvarez-Idaboy J. R. Russo N. (2016). Food antioxidants: chemical insights at the molecular level. Annu. Rev. food Sci. Technol. 7, 335–352. 10.1146/annurev-food-041715-033206 26772412

[B26] Geerlings P. De Proft F. Langenaeker W. (2003). Conceptual density functional theory. Chem. Rev. 103 (5), 1793–1874. 10.1021/cr990029p 12744694

[B27] Ghabbour H. A. Bakheit A. H. Ezzeldin E. Mostafa G. A. E. (2022). Synthesis characterization and X-ray structure of 2-(2,6-Dichlorophenylamino)-2-imidazoline tetraphenylborate: computational study. Appl. Sci. 12 (7), 3568. 10.3390/app12073568

[B28] Irikura K. K. Johnson R. D. Kacker R. N. (2005). Uncertainties in scaling factors for *ab initio* vibrational frequencies. J. Phys. Chem. A 109 (37), 8430–8437. 10.1021/jp052793n 16834237

[B29] Klein E. Lukeš V. (2006). DFT/B3LYP study of the substituent effect on the reaction enthalpies of the individual steps of single electron Transfer−Proton transfer and sequential proton loss electron transfer mechanisms of phenols antioxidant action. J. Phys. Chem. A 110 (44), 12312–12320. 10.1021/jp063468i 17078630

[B30] Kotaiah Y. Harikrishna N. Nagaraju K. Rao C. V. (2012). Synthesis and antioxidant activity of 1, 3, 4-oxadiazole tagged thieno [2, 3-d] pyrimidine derivatives. Eur. J. Med. Chem. 58, 340–345. 10.1016/j.ejmech.2012.10.007 23149297

[B31] Lee C. Yang W. Parr R. G. (1988). Development of the Colle-Salvetti correlation-energy formula into a functional of the electron density. Phys. Rev. B 37 (2), 785–789. 10.1103/physrevb.37.785 9944570

[B32] Leopoldini M. Russo N. Toscano M. (2011). The molecular basis of working mechanism of natural polyphenolic antioxidants. Food Chem. 125 (2), 288–306. 10.1016/j.foodchem.2010.08.012

[B33] Li S.-G. Vilchèze C. Chakraborty S. Wang X. Kim H. Anisetti M. (2015). Evolution of a thienopyrimidine antitubercular relying on medicinal chemistry and metabolomics insights. Tetrahedron Lett. 56 (23), 3246–3250. 10.1016/j.tetlet.2015.02.129 26257441 PMC4527344

[B34] Lobo V. Patil A. Phatak A. Chandra N. (2010). Free radicals, antioxidants and functional foods: impact on human health. Pharmacogn. Rev. 4 (8), 118. 10.4103/0973-7847.70902 22228951 PMC3249911

[B35] Lončar A. Negrojević L. Dimitrić-Marković J. Dimić D. (2021). The reactivity of neurotransmitters and their metabolites towards various nitrogen-centered radicals: experimental, theoretical, and biotoxicity evaluation. Comput. Biol. Chem. 95, 107573. 10.1016/j.compbiolchem.2021.107573 34562727

[B36] Lü J. M. Lin P. H. Yao Q. Chen C. (2010). Chemical and molecular mechanisms of antioxidants: experimental approaches and model systems. J. Cell. Mol. Med. 14 (4), 840–860. 10.1111/j.1582-4934.2009.00897.x 19754673 PMC2927345

[B37] Marković Z. Tošović J. Milenković D. Marković S. (2016). Revisiting the solvation enthalpies and free energies of the proton and electron in various solvents. Comput. Theor. Chem. 1077, 11–17. 10.1016/j.comptc.2015.09.007

[B38] Martínez-Araya J. I. (2015). Why is the dual descriptor a more accurate local reactivity descriptor than Fukui functions? J. Math. Chem. 53 (2), 451–465. 10.1007/s10910-014-0437-7

[B39] Mavrova A. T. Dimov S. Yancheva D. Rangelov M. Wesselinova D. Tsenov J. A. (2016). Synthesis, anticancer activity and photostability of novel 3-ethyl-2-mercapto-thieno[2,3-d]pyrimidin-4(3H)-ones. Eur. J. Med. Chem. 123, 69–79. 10.1016/j.ejmech.2016.07.022 27474924

[B40] Miertuš S. Scrocco E. Tomasi J. (1981). Electrostatic interaction of a solute with a continuum. A direct utilizaion of *ab initio* molecular potentials for the prevision of solvent effects. Chem. Phys. 55 (1), 117–129. 10.1016/0301-0104(81)85090-2

[B41] Miertus S. Tomasi J. (1982). Approximate evaluations of the electrostatic free energy and internal energy changes in solution processes. Chem. Phys. 65 (2), 239–245. 10.1016/0301-0104(82)85072-6

[B42] Morell C. Grand A. Toro-Labbé A. (2005). New dual descriptor for chemical reactivity. J. Phys. Chem. A 109 (1), 205–212. 10.1021/jp046577a 16839107

[B43] Morell C. Grand A. Toro-Labbé A. (2006). Theoretical support for using the Δf (r) descriptor. Chem. Phys. Lett. 425 (4-6), 342–346. 10.1016/j.cplett.2006.05.003

[B44] Mostafa G. A. Bakheit A. AlMasoud N. AlRabiah H. (2021). Charge transfer complexes of ketotifen with 2, 3-dichloro-5, 6-dicyano-p-benzoquinone and 7, 7, 8, 8-tetracyanoquodimethane: spectroscopic characterization studies. Molecules 26 (7), 2039. 10.3390/molecules26072039 33918481 PMC8038309

[B45] Mostafa G. A. E. Bakheit A. H. Al-Agamy M. H. Al-Salahi R. Ali E. A. Alrabiah H. (2023). Synthesis of 4-amino-N-[2 (diethylamino)Ethyl]Benzamide tetraphenylborate ion-associate complex: characterization, antibacterial and computational study. Molecules 28 (5), 2256. 10.3390/molecules28052256 36903501 PMC10005259

[B46] Niki E. (2016). Antioxidant capacity of foods for scavenging reactive oxidants and inhibition of plasma lipid oxidation induced by multiple oxidants. Food and Funct. 7 (5), 2156–2168. 10.1039/c6fo00275g 27090496

[B47] Parker V. D. (1992). Homolytic bond (HA) dissociation free energies in solution. Applications of the standard potential of the (H+/H. bul.) couple. J. Am. Chem. Soc. 114 (19), 7458–7462. 10.1021/ja00045a018

[B48] Raghavan N. Steenken S. (1980). Electrophilic reaction of the hydroxyl radical with phenol. Determination of the distribution of isomeric dihydroxycyclohexadienyl radicals. J. Am. Chem. Soc. 102 (10), 3495–3499. 10.1021/ja00530a031

[B49] Rizk O. H. Shaaban O. G. El-Ashmawy I. M. (2012). Design, synthesis and biological evaluation of some novel thienopyrimidines and fused thienopyrimidines as anti-inflammatory agents. Eur. J. Med. Chem. 55, 85–93. 10.1016/j.ejmech.2012.07.007 22835720

[B50] Roder M. Wojnarovits L. Földiák G. Emmi S. Beggiato G. D’Angelantonio M. (1999). Addition and elimination kinetics in OH radical induced oxidation of phenol and cresols in acidic and alkaline solutions. Radiat. Phys. Chem. 54 (5), 475–479. 10.1016/s0969-806x(98)00294-1

[B51] Sadasivam K. Kumaresan R. (2011). Theoretical investigation on the antioxidant behavior of chrysoeriol and hispidulin flavonoid compounds–A DFT study. Comput. Theor. Chem. 963 (1), 227–235. 10.1016/j.comptc.2010.10.025

[B52] Sastre S. Frau J. Glossman-Mitnik D. (2017). Computational prediction of the protonation sites of Ac-Lys-(Ala) n-Lys-NH2 peptides through conceptual DFT descriptors. Molecules 22 (3), 458. 10.3390/molecules22030458 28335381 PMC6155279

[B53] Senthilkumar K. (2021). Theoretical insights on the antioxidant activity of dihydrocaffeic acid. Mater. Today Proc. 47, 1868–1872. 10.1016/j.matpr.2021.03.522

[B54] Sezgin B. Tilki T. Karabacak Atay Ç. Dede B. (2022). Comparative *in vitro* and DFT antioxidant studies of phenolic group substituted pyridine-based azo derivatives. J. Biomol. Struct. Dyn. 40 (11), 4921–4932. 10.1080/07391102.2020.1863264 33357036

[B55] Stephens P. J. Devlin F. J. Chabalowski C. F. Frisch M. J. (1994). *Ab initio* calculation of vibrational absorption and circular dichroism spectra using density functional force fields. J. Phys. Chem. 98 (45), 11623–11627. 10.1021/j100096a001

[B56] Toro-Labbé A. (2006). Theoretical aspects of chemical reactivity. Elsevier.

[B57] Vásquez-Espinal A. Yañez O. Osorio E. Areche C. García-Beltrán O. Ruiz L. M. (2019). Theoretical study of the antioxidant activity of quercetin oxidation products. Front. Chem. 7, 818. 10.3389/fchem.2019.00818 31828060 PMC6890856

[B58] Vo Q. V. Nam P. C. Bay M. V. Thong N. M. Cuong N. D. Mechler A. (2018). Density functional theory study of the role of benzylic hydrogen atoms in the antioxidant properties of lignans. Sci. Rep. 8 (1), 12361. 10.1038/s41598-018-30860-5 30120382 PMC6098005

[B59] Wang G. Liu Y. Zhang L. An L. Chen R. Liu Y. (2020). Computational study on the antioxidant property of coumarin-fused coumarins. Food Chem. 304, 125446. 10.1016/j.foodchem.2019.125446 31491715

