# Supplementary material for: Theoretical study of the antioxidant mechanism and structure-activity relationships of 1,3,4-oxadiazol-2-ylthieno[2,3-d]pyrimidin-4-amine derivatives: a computational approach
Source: Front Chem. 2024 Jul 30;12:1443718. doi: 10.3389/fchem.2024.1443718 (PMC11319267; doi:10.3389/fchem.2024.1443718)
Supplement: Supplementary file 1 [file DataSheet1.docx]

**Detailed Computational Methods**

**Antioxidant Reaction Mechanisms**

- **Hydrogen Atom Transfer (HAT):**

$R-H\to R^{\cdot}+H^{\cdot}\left( BDE \right)$………………………………..S1

- **Single Electron Transfer-Proton Transfer (SET-PT):**

In the “Single electron transfer followed by proton transfer” (SETPT) mechanism the first step is electron loss to form a radical cation, characterized by the ionization energy, followed by a deprotonation step that is described with the proton dissociation energy.

.$R-H\to{RH}^{+\cdot}+e^{-} (IP)$……………………………..S2

${RH}^{+\cdot}\to R^{\cdot}+H^{+} (PDE)$………………………………..S3

- **The third mechanism, “Sequential proton loss electron transfer” (SPLET), starts with the**

The third mechanism, “Sequential proton loss electron transfer” (SPLET), starts with the dissociation of the acidic moiety, which can be characterized by the proton affinity; this is followed by an electron transfer to the free radical, at a cost of the electron transfer energy

$R-H\to R^{-}+H^{+} (PA)$……………………..…………..S4

$R^{-}\to R^{\cdot}+e^{-} (ELE)$………………………….……………………S5

- **Sequential Proton Loss Electron Transfer (SPLET):**

Sequential steps of proton loss and electron transfer.

**Reaction Enthalpies Calculation**

- **Conditions:** Gas phase at 298.15 K and 1 atm.
- **Equations Used:** Formulae for BDE, IE, PDE, PA, and ETE involving enthalpies of specific molecular states. For instance:

$BDE=H\left( R^{\cdot} \right)+H\left( H^{\cdot} \right)-H(R-H)$…………………………..S6

$IP=H\left( {RH}^{\cdot+} \right)+H\left( e^{-} \right)-H(R-H)$…………………………S7

$PDE=H\left( R^{\cdot} \right)+H\left( H^{+} \right)-H({RH}^{+\cdot})$…………………….…….S8

$PA=H\left( R^{-} \right)+H\left( H^{+} \right)-H(R-H)$…………………….…..S9

$ELE=H\left( R^{\cdot} \right)+H\left( e^{-} \right)-H(R^{-})$……………………….……S10

| 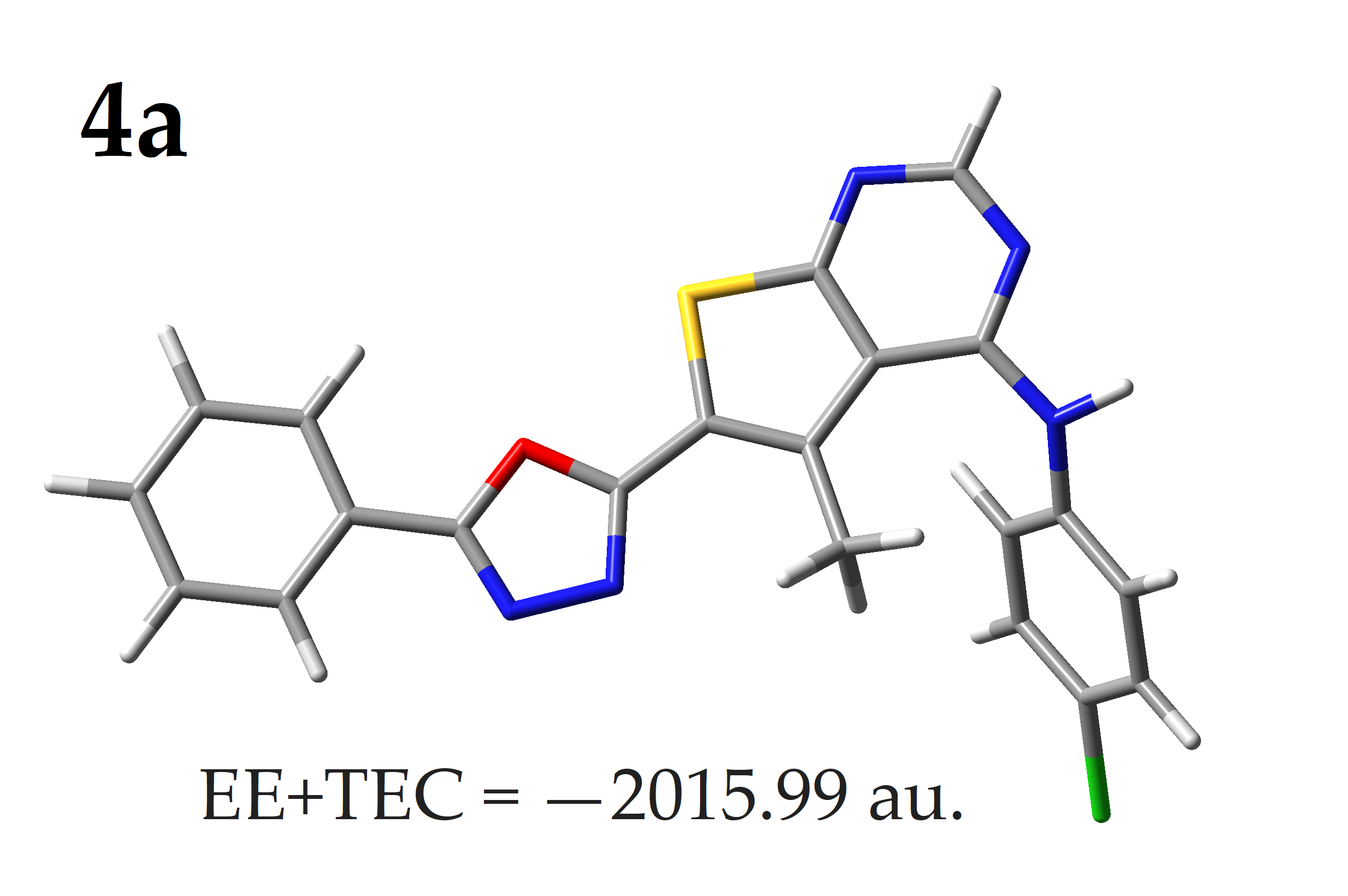 | 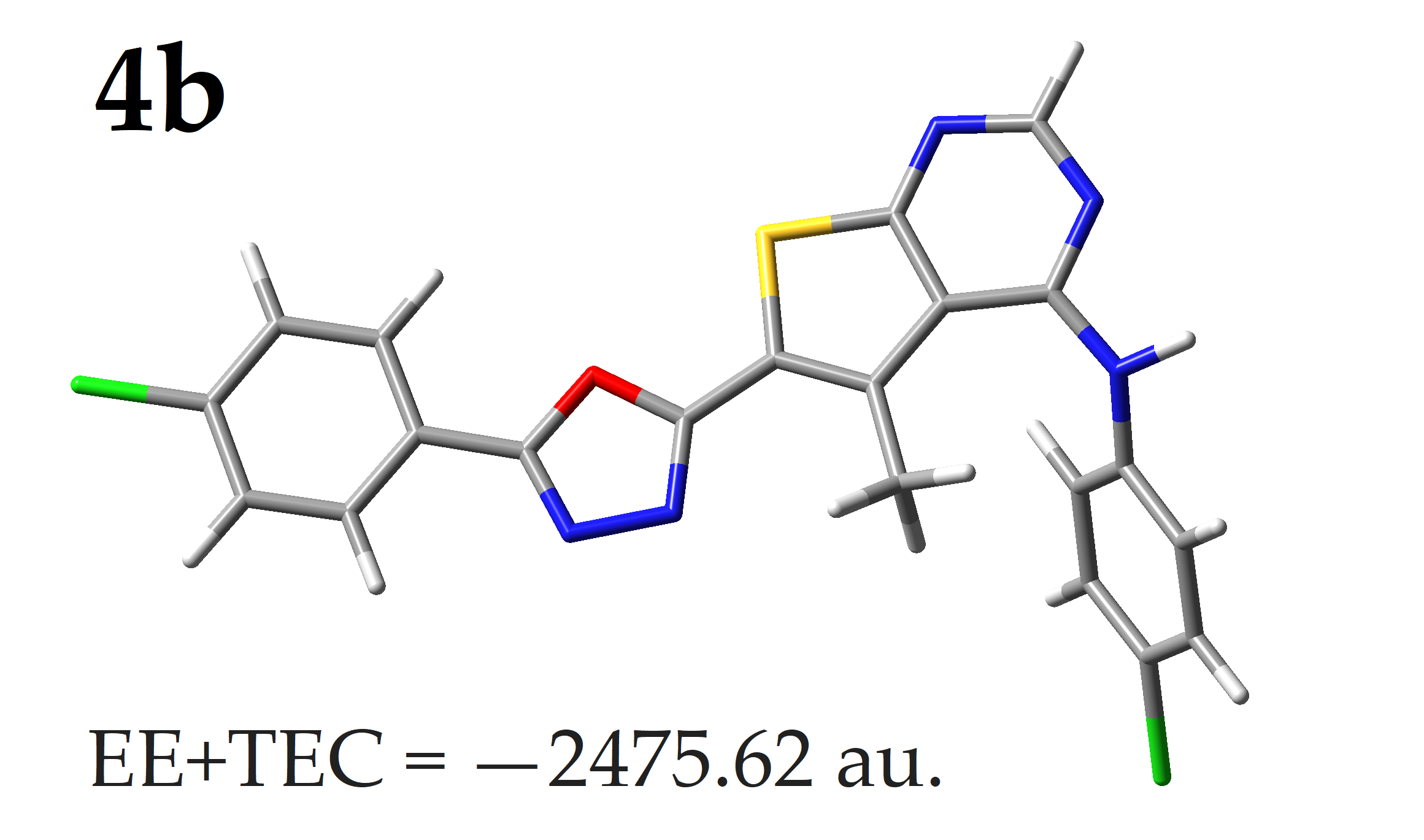 | 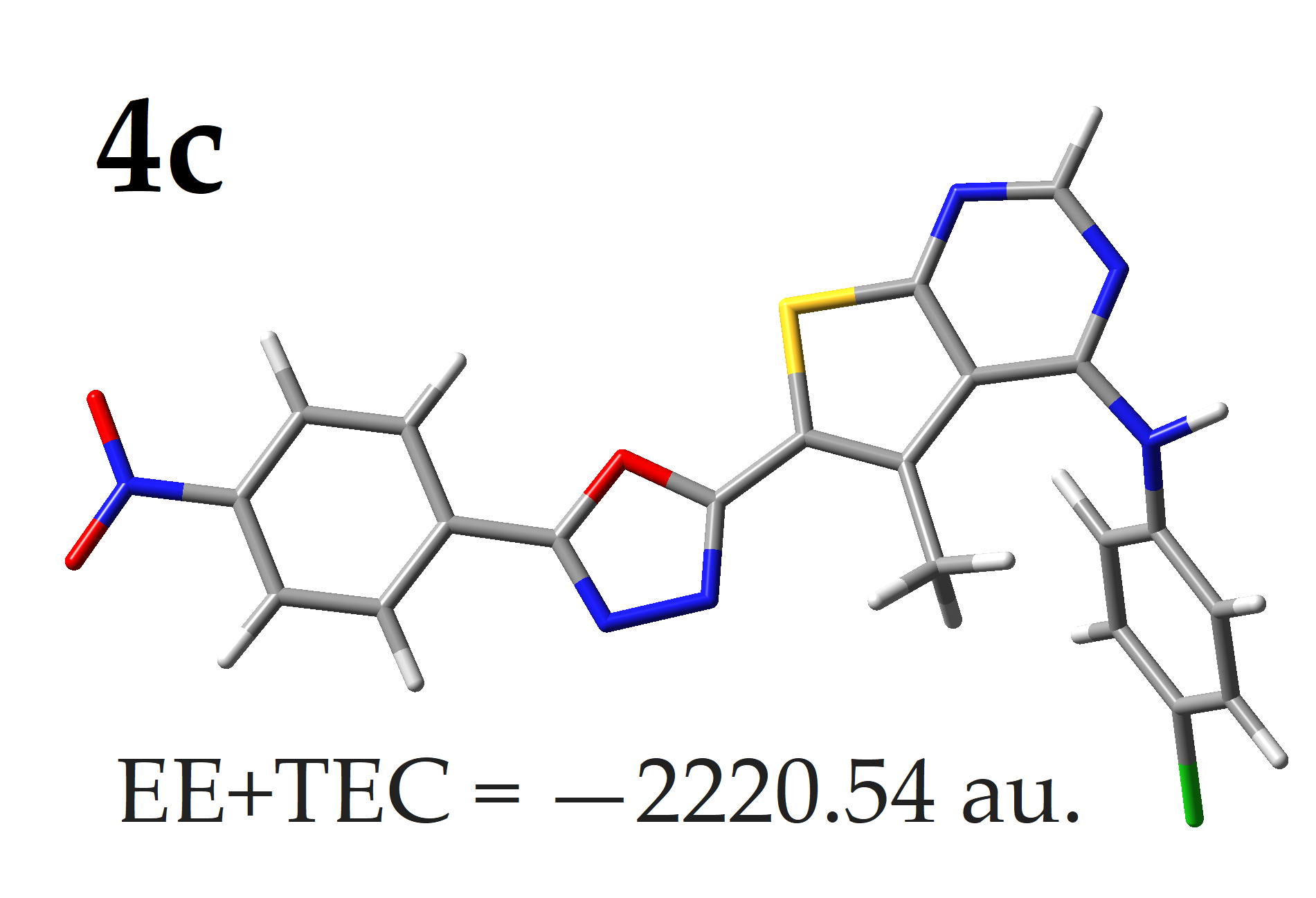 |
| --- | --- | --- |
| 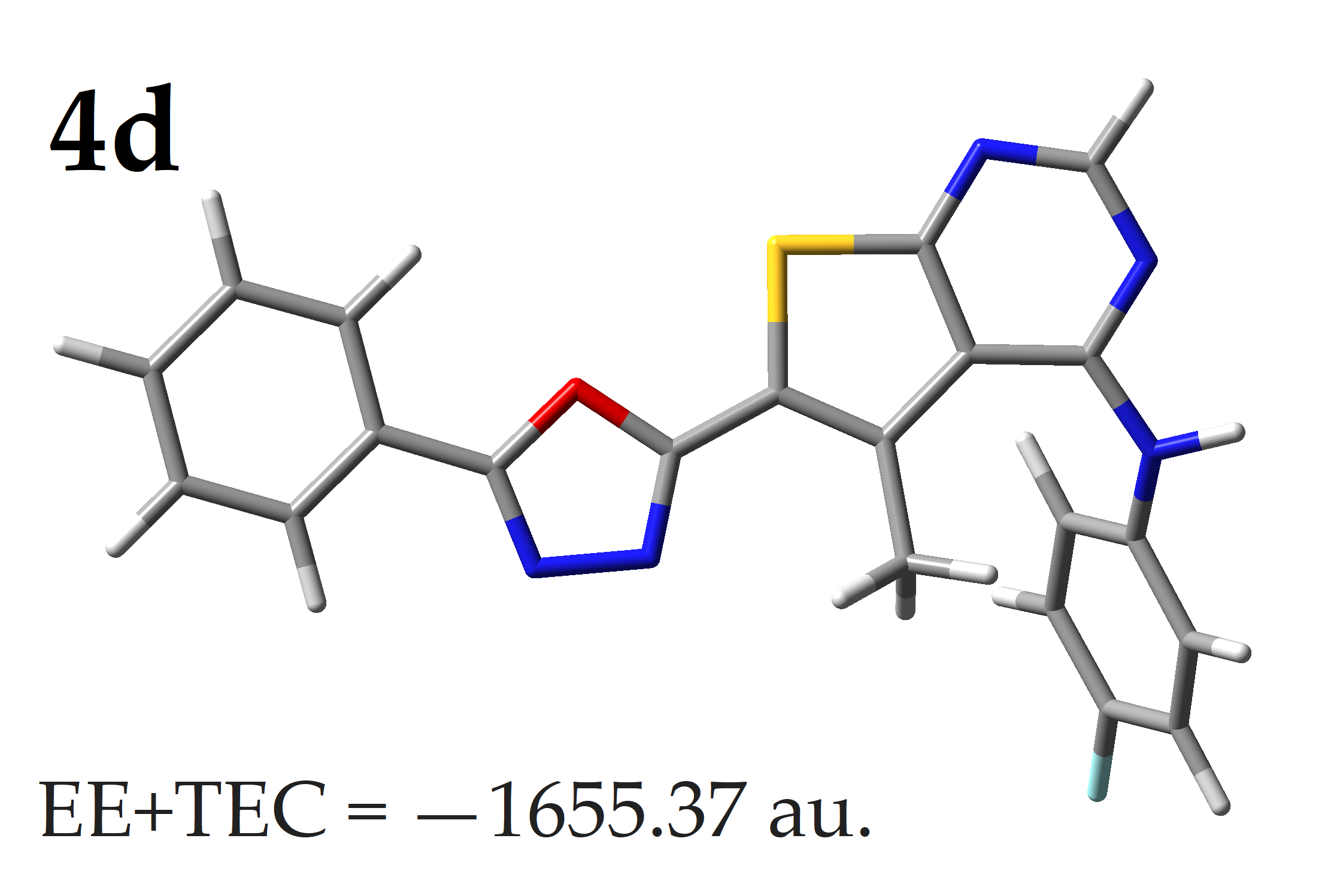 | 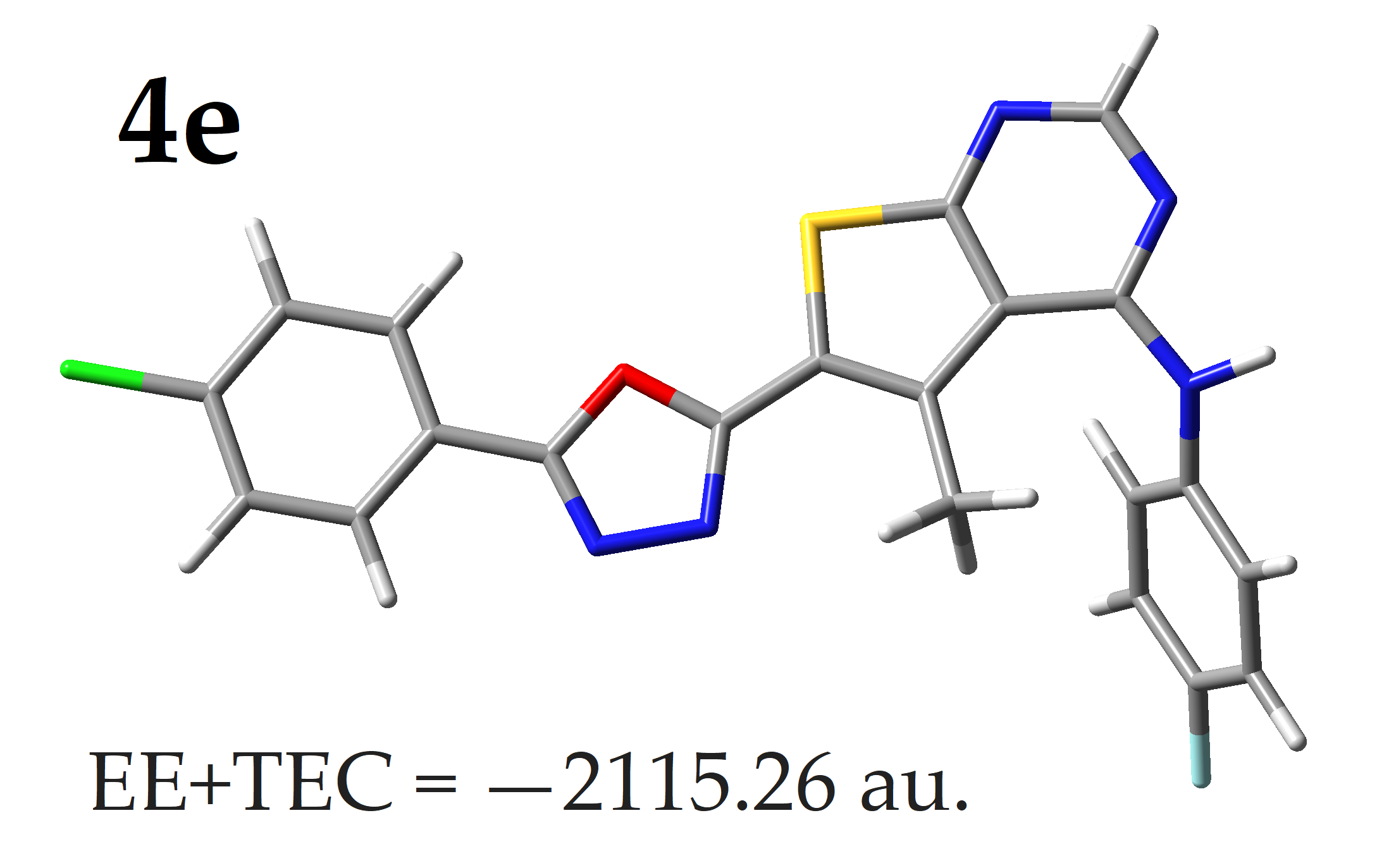 | 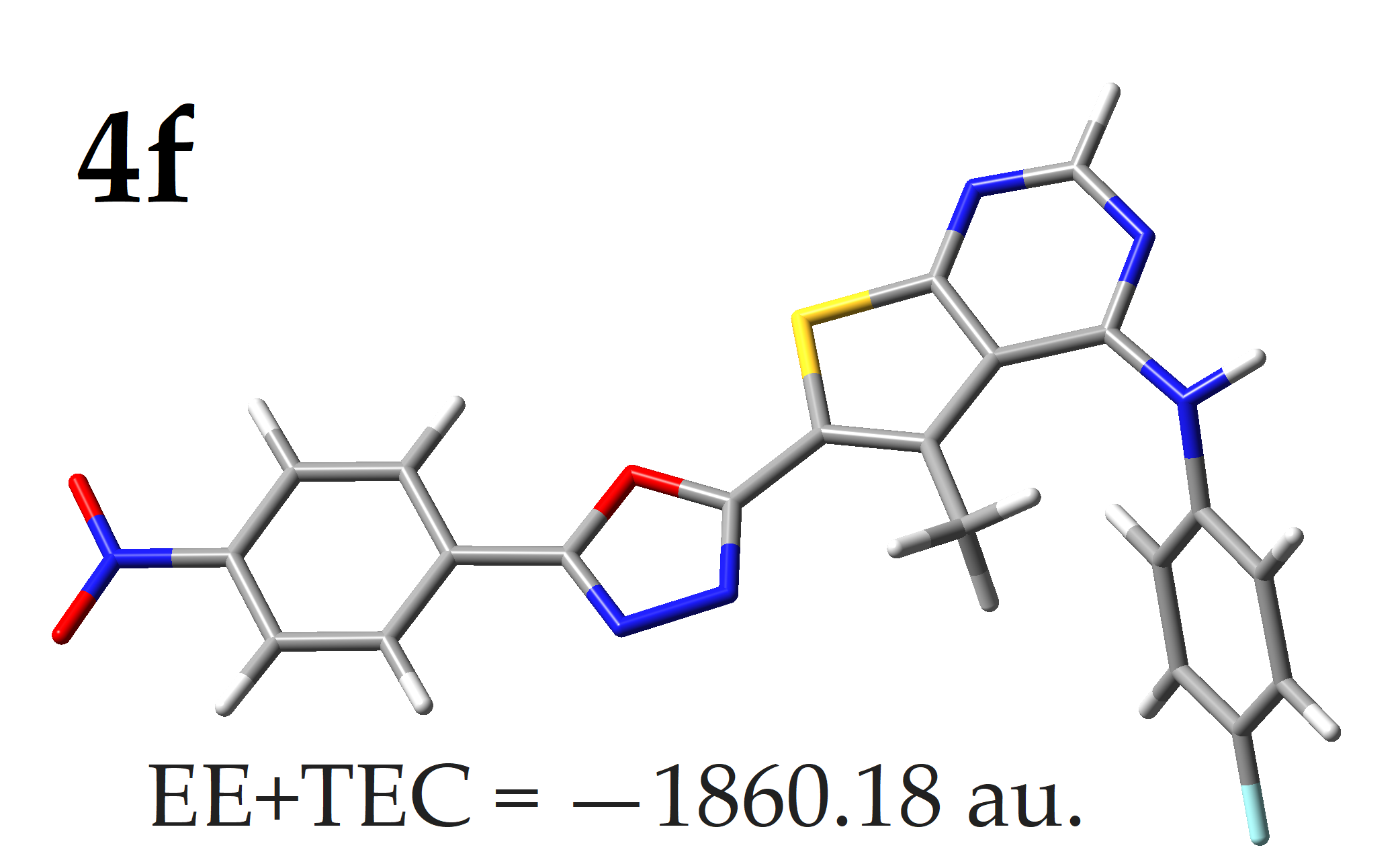 |
| 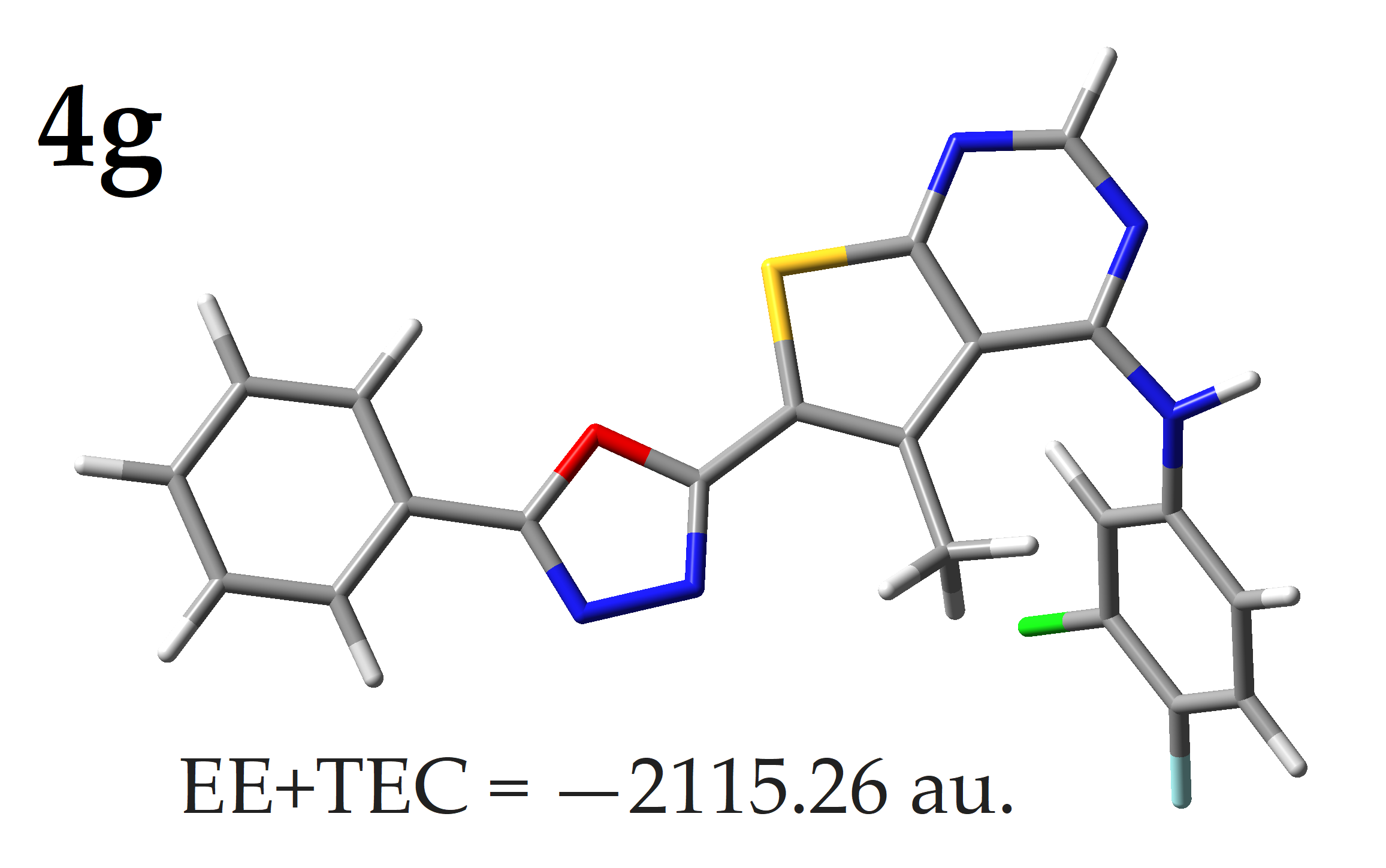 | 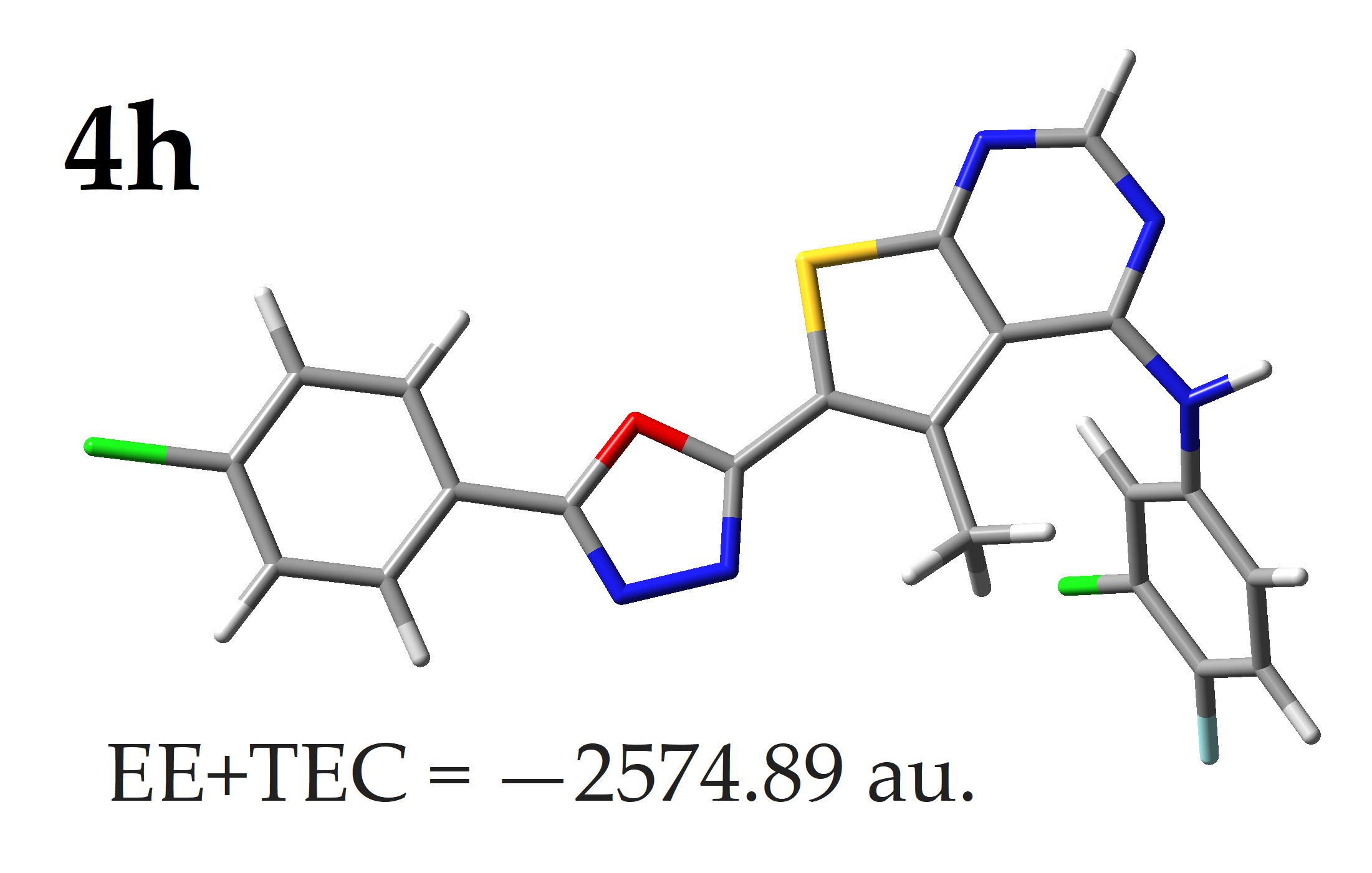 | 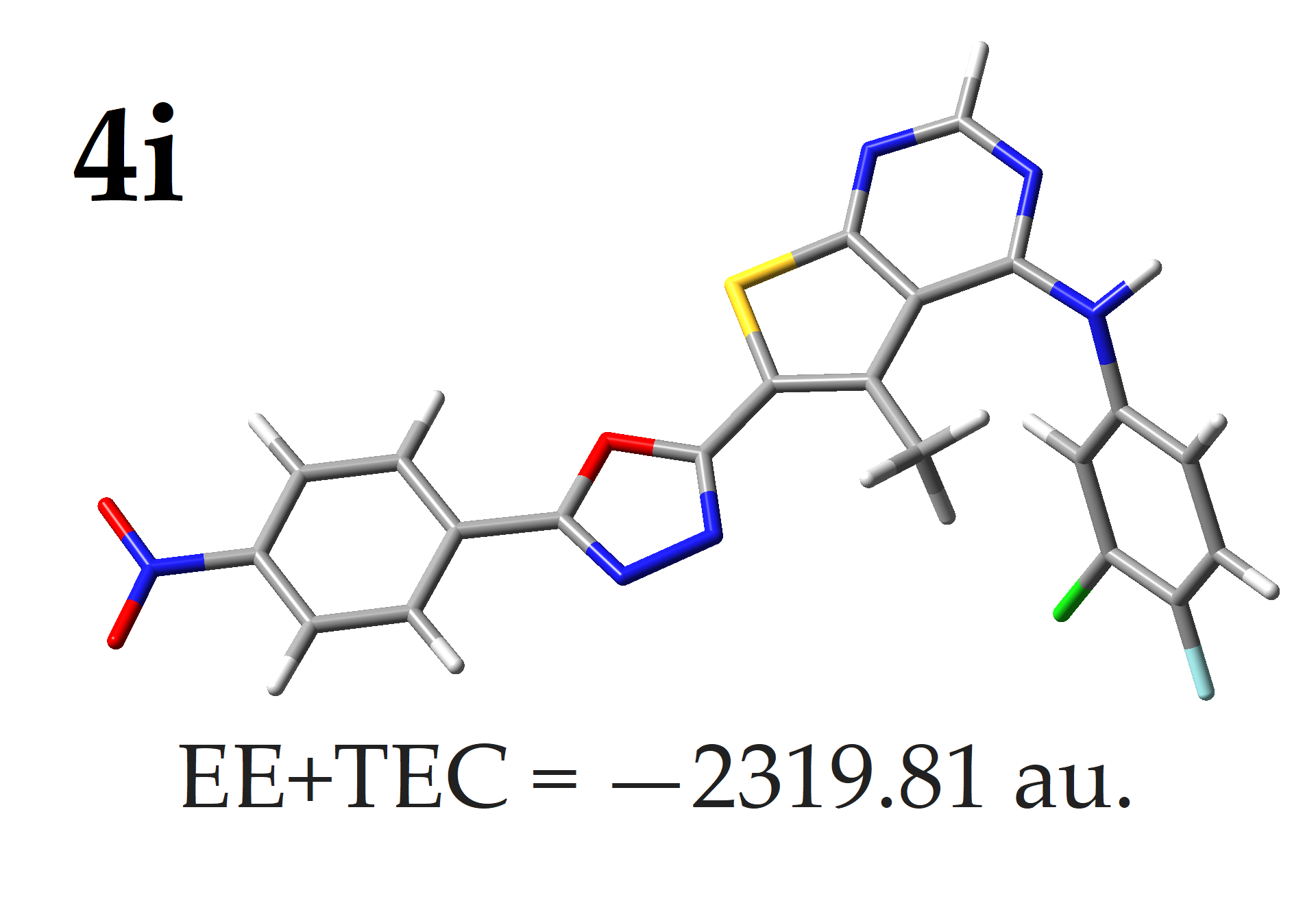 |
| 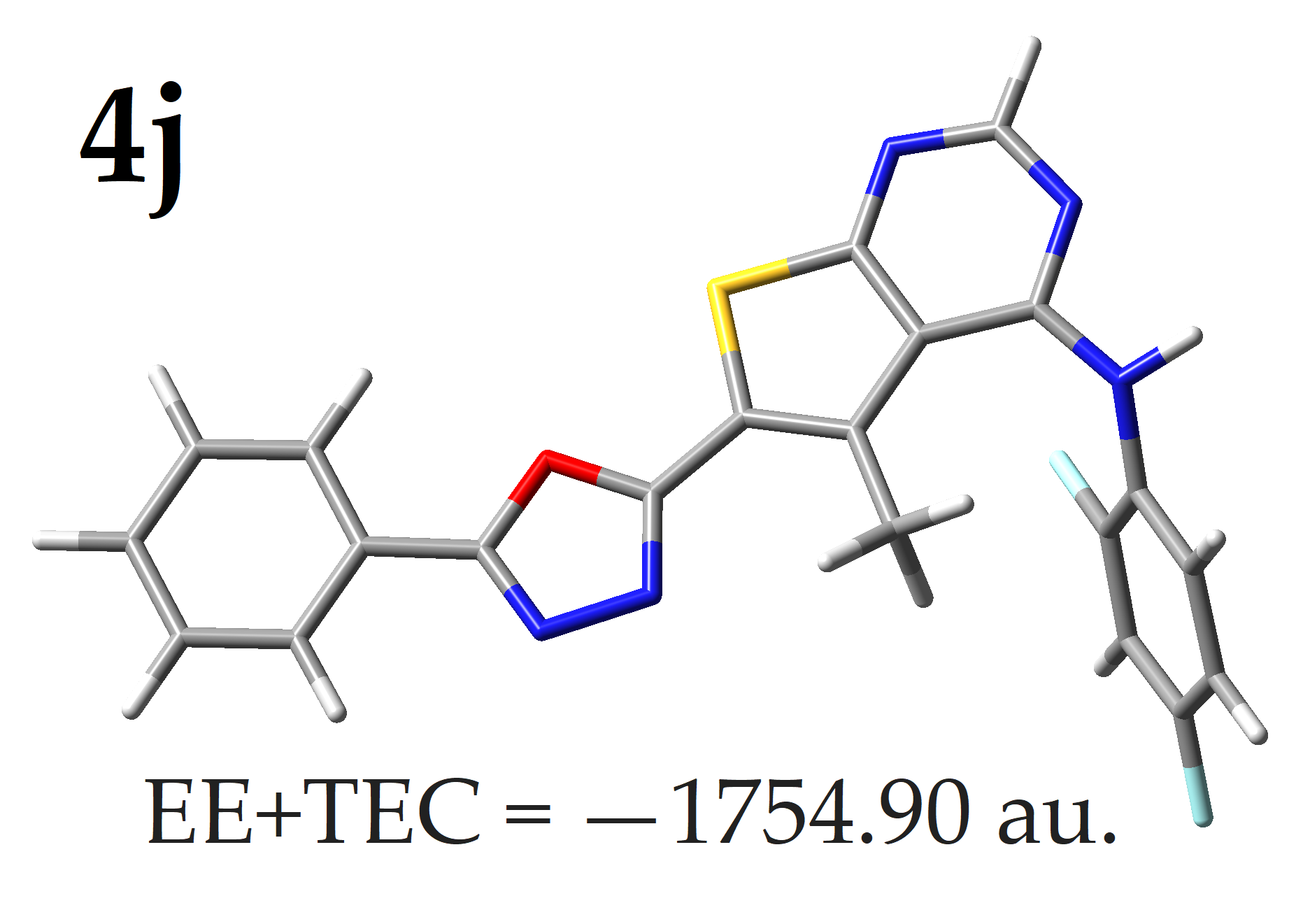 | 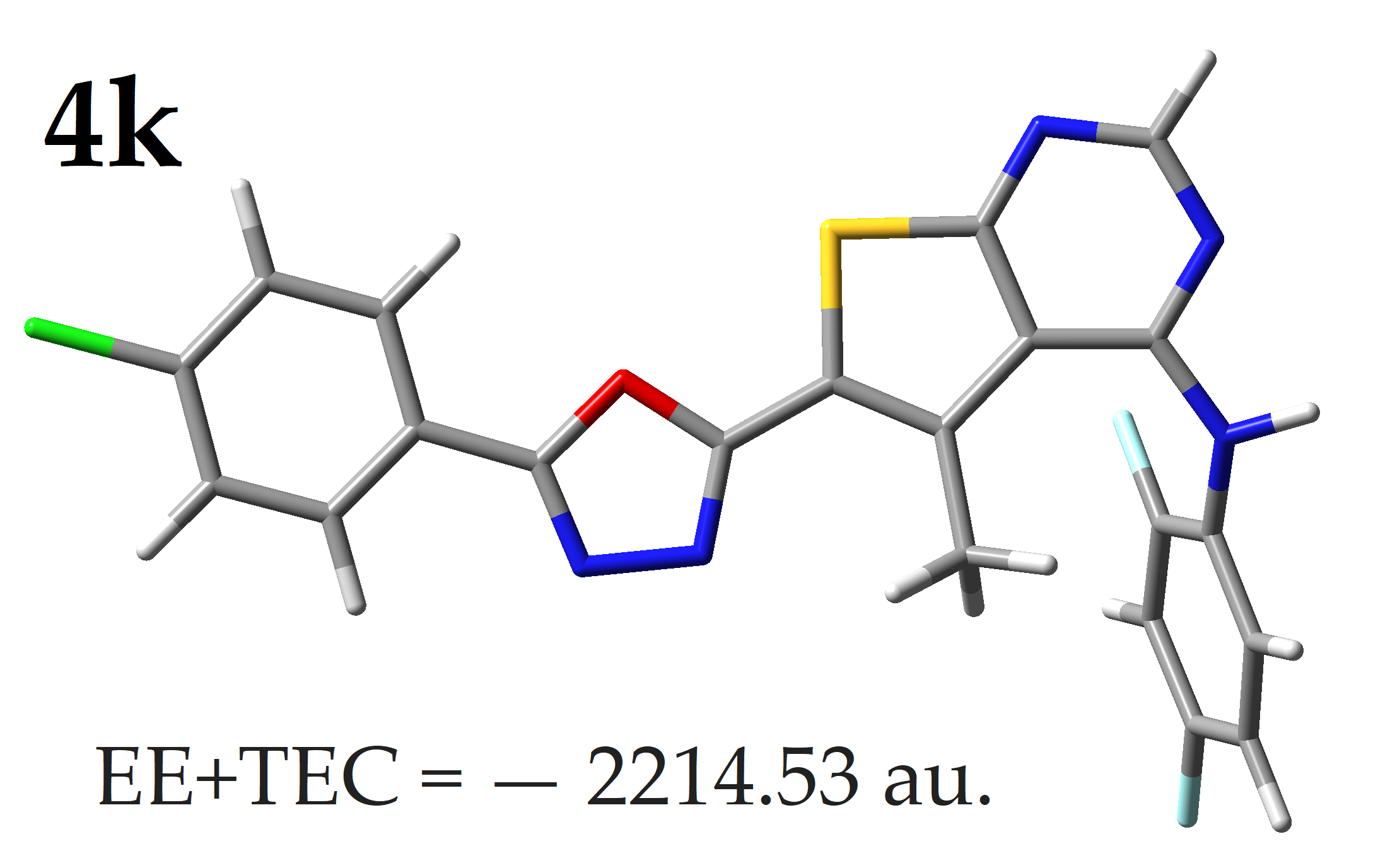 | 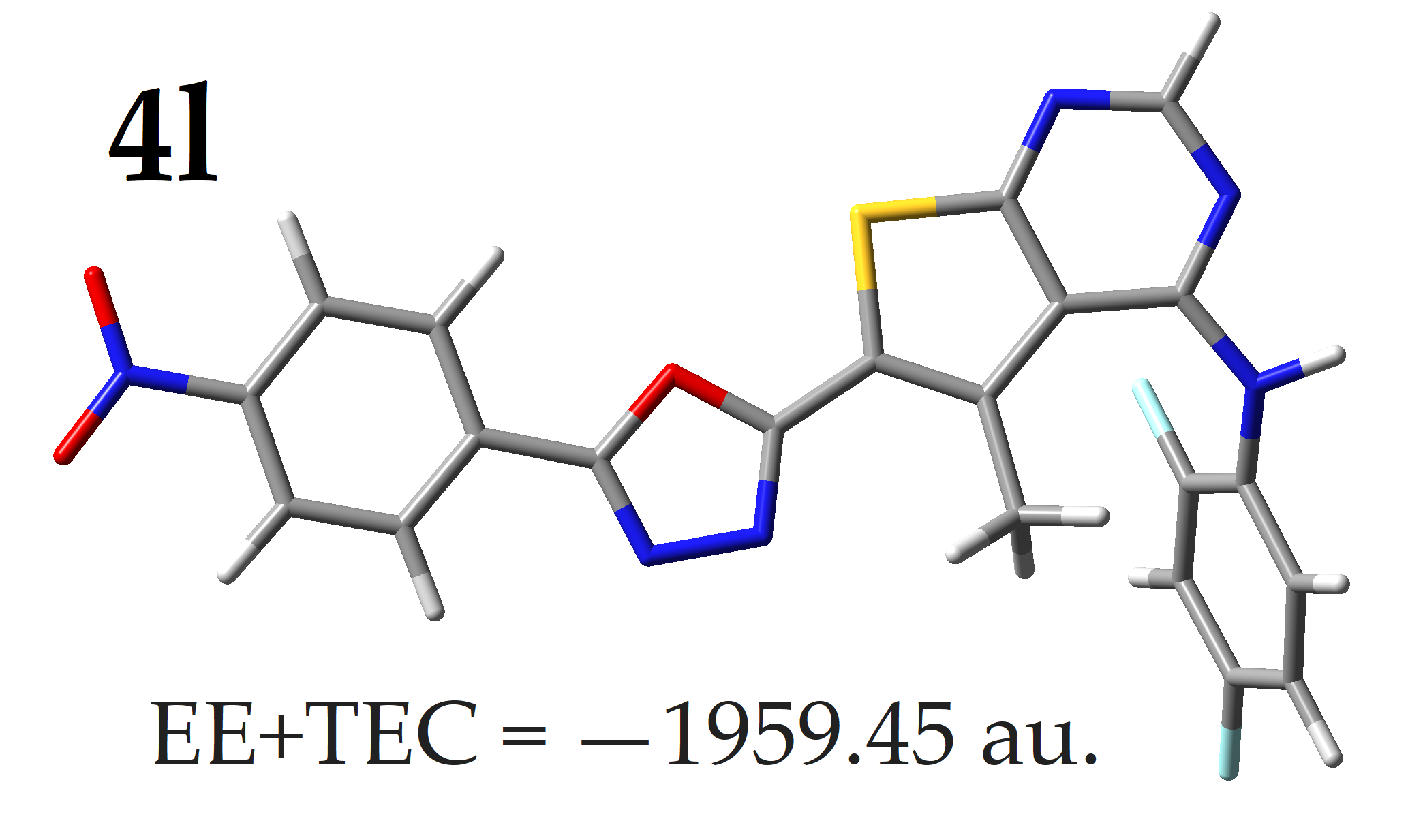 |

**Figure S1:** Optimized Geometries of the Most Stable Structures of Compounds 4a-4l. This figure shows the neutral forms of these compounds in the gas phase, optimized at the B3LYP/6-311G(d,p) level of theory. Including Electronic Energy (EE) and Thermal Free Energy Correction (TEC)

| HOMO | LUMO |
| --- | --- |
| 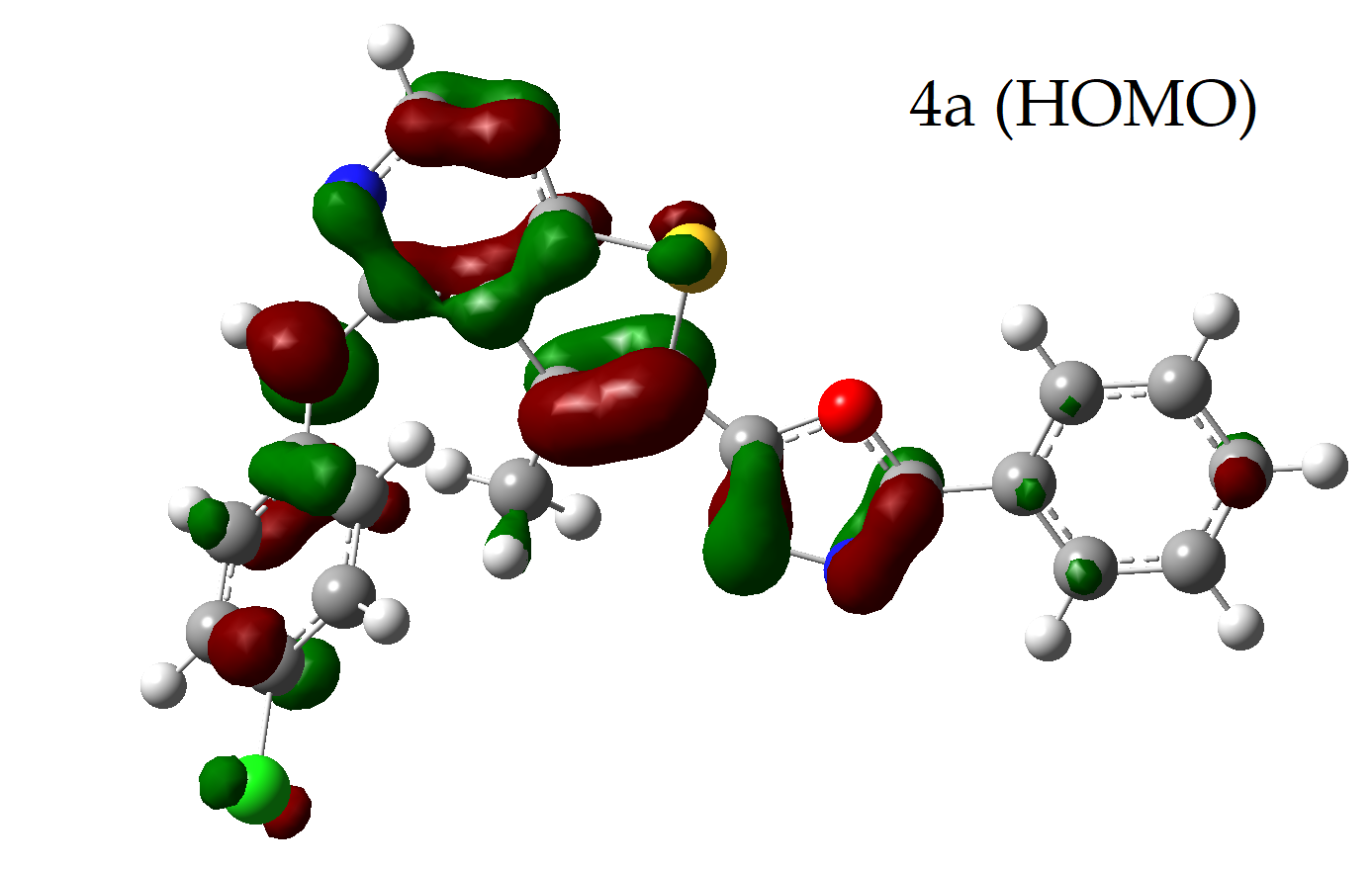 | 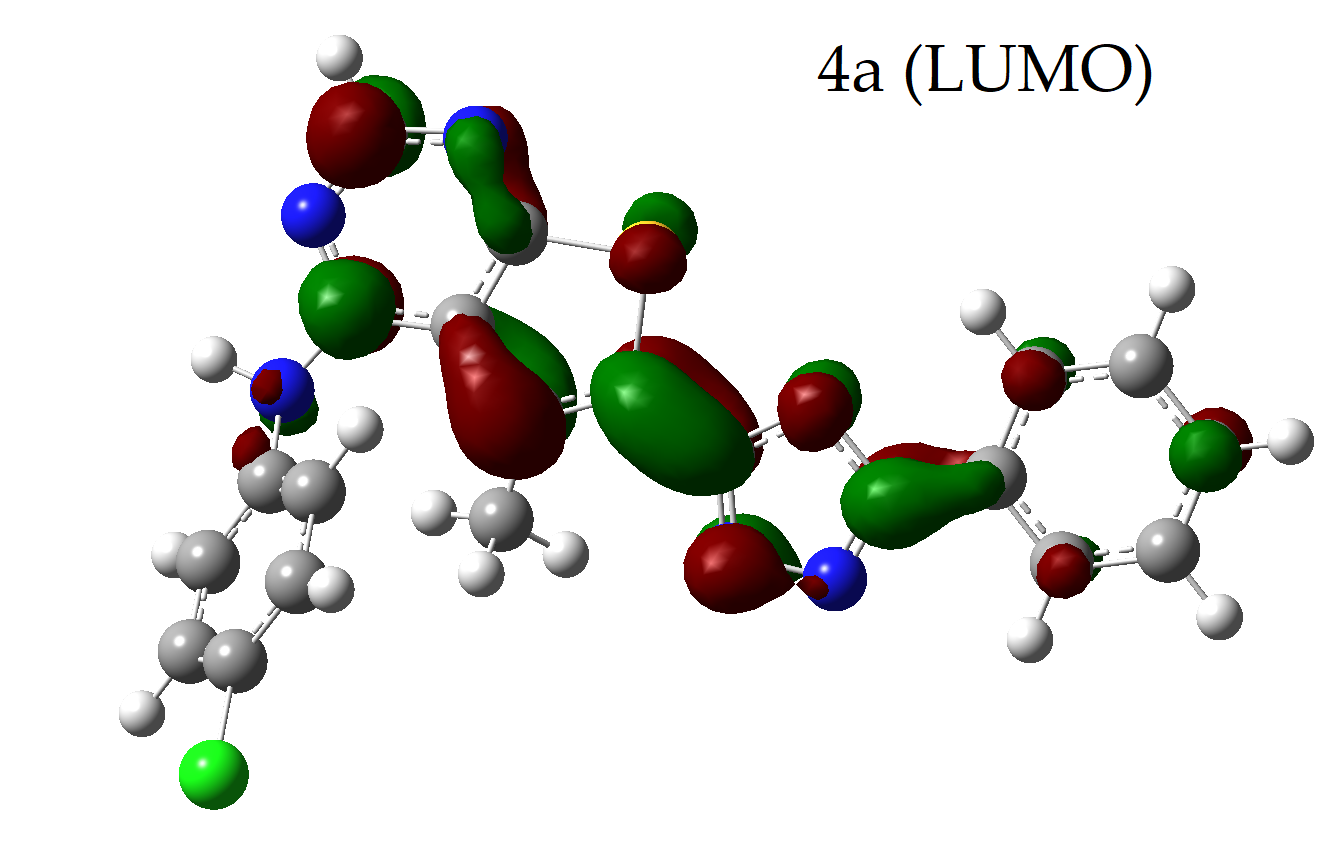 |
| 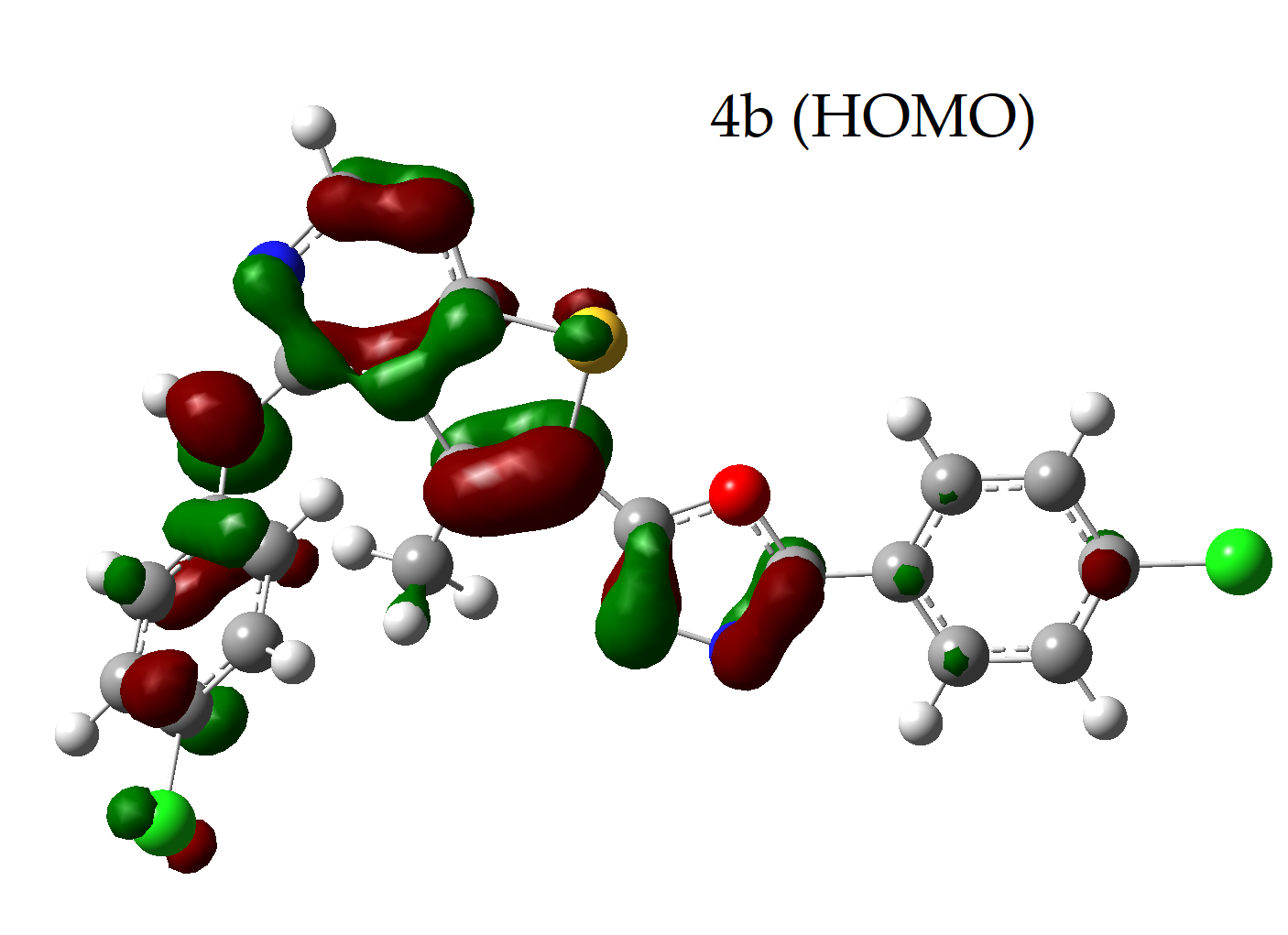 | 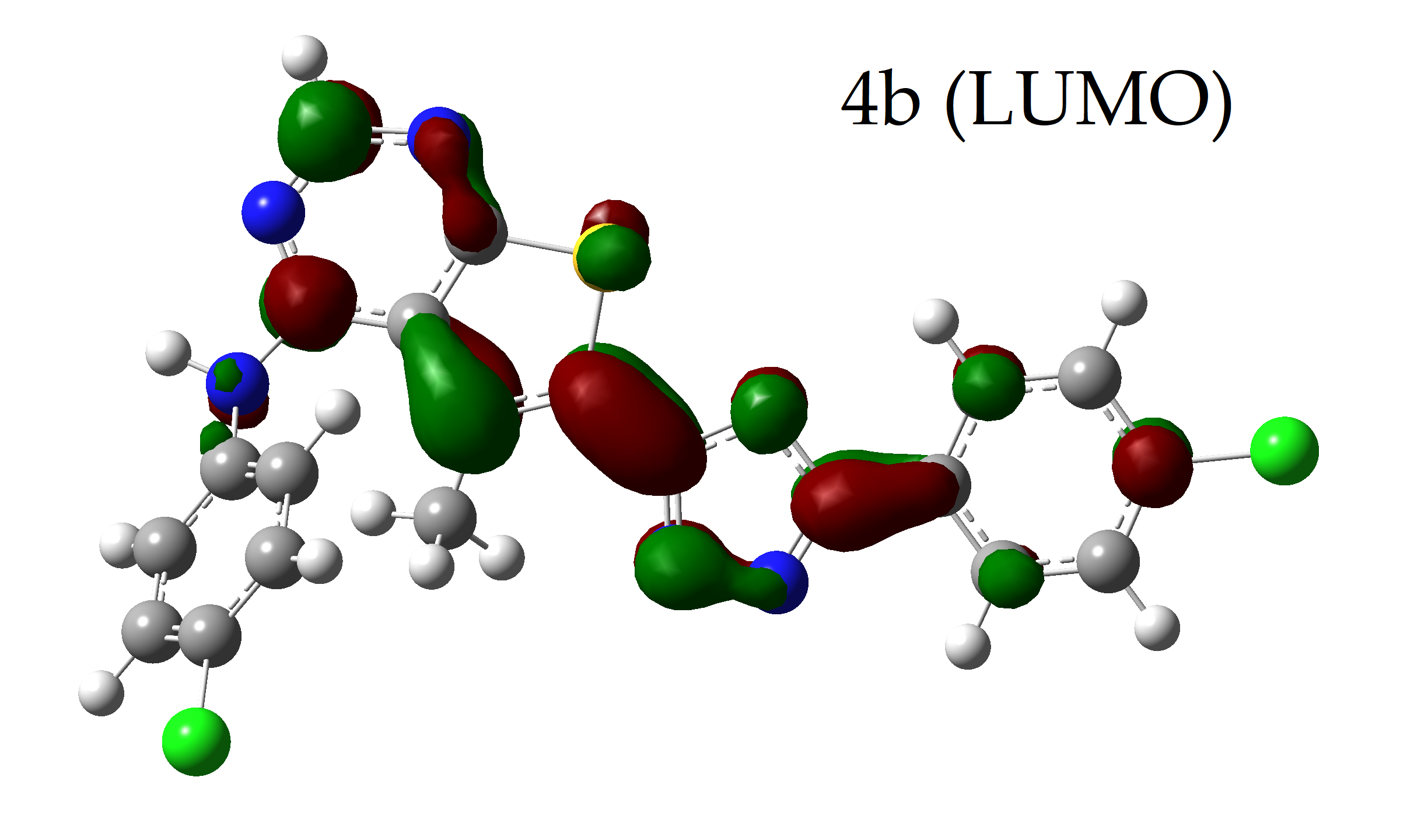 |
| 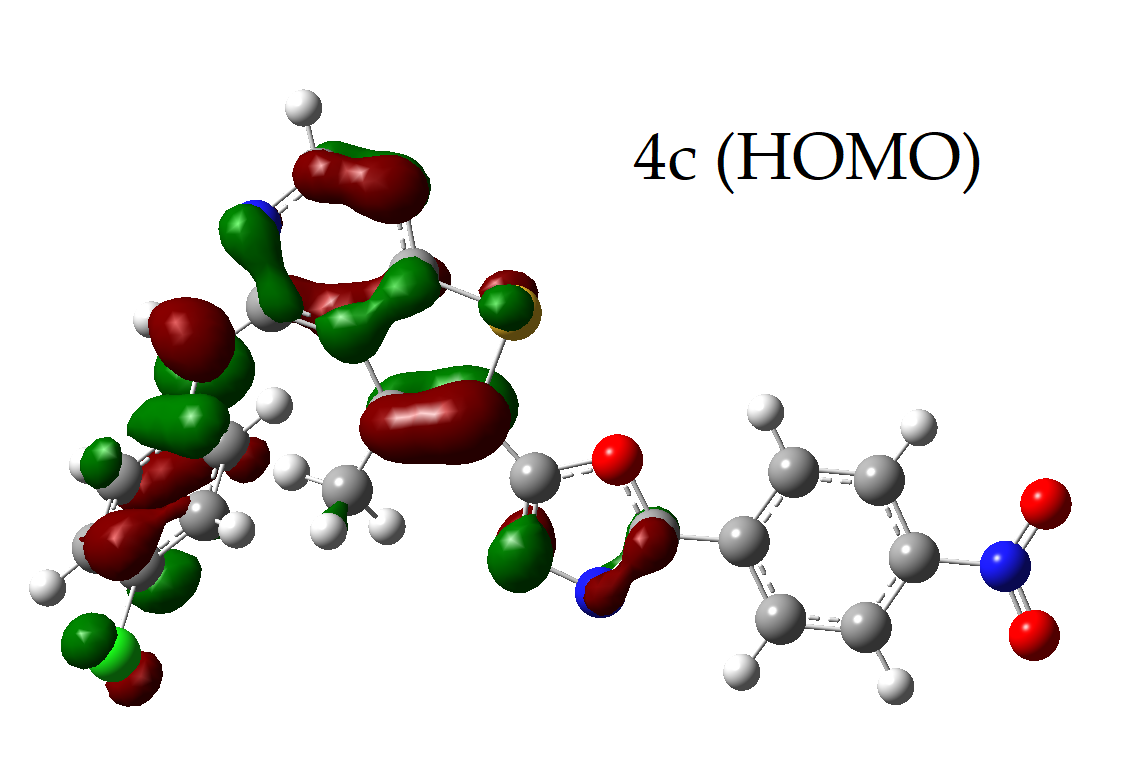 | 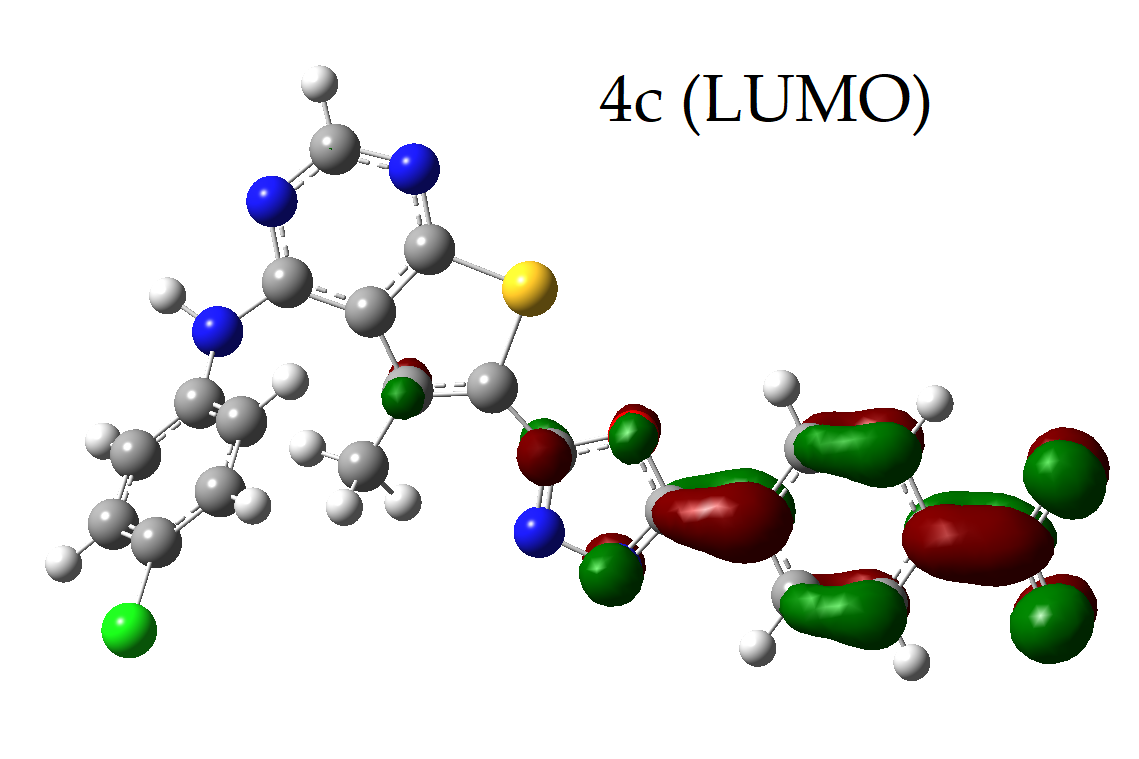 |
| 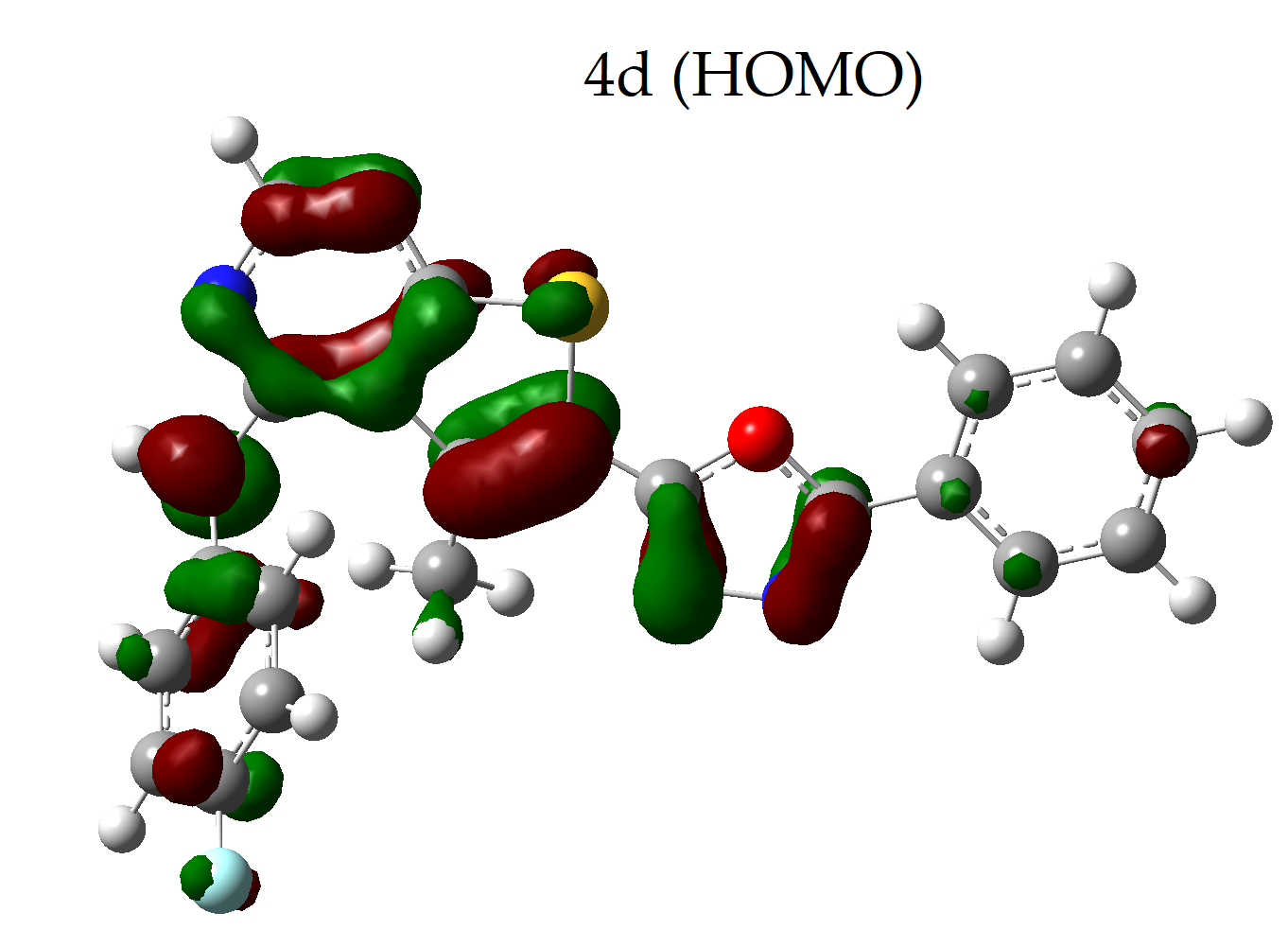 | 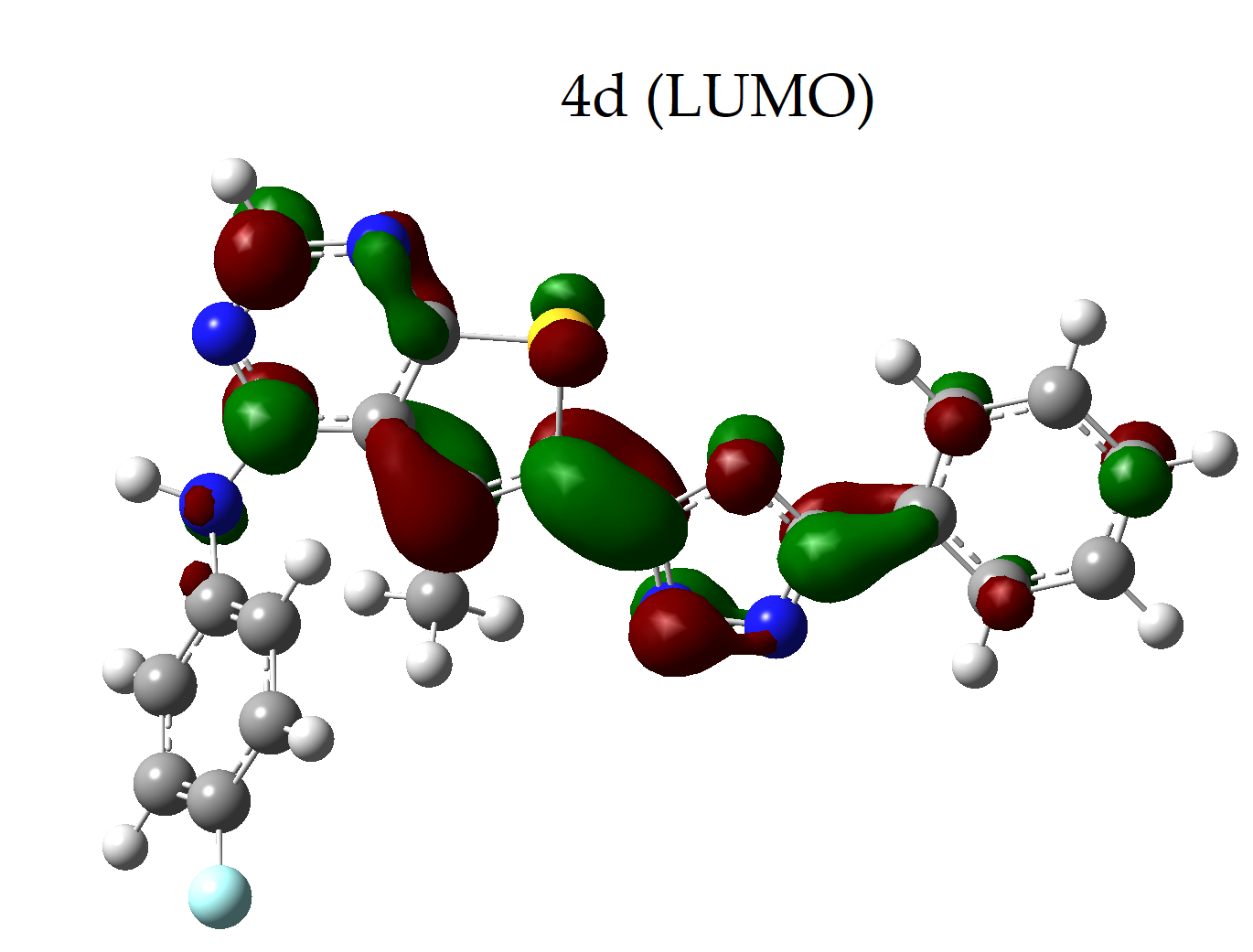 |
| 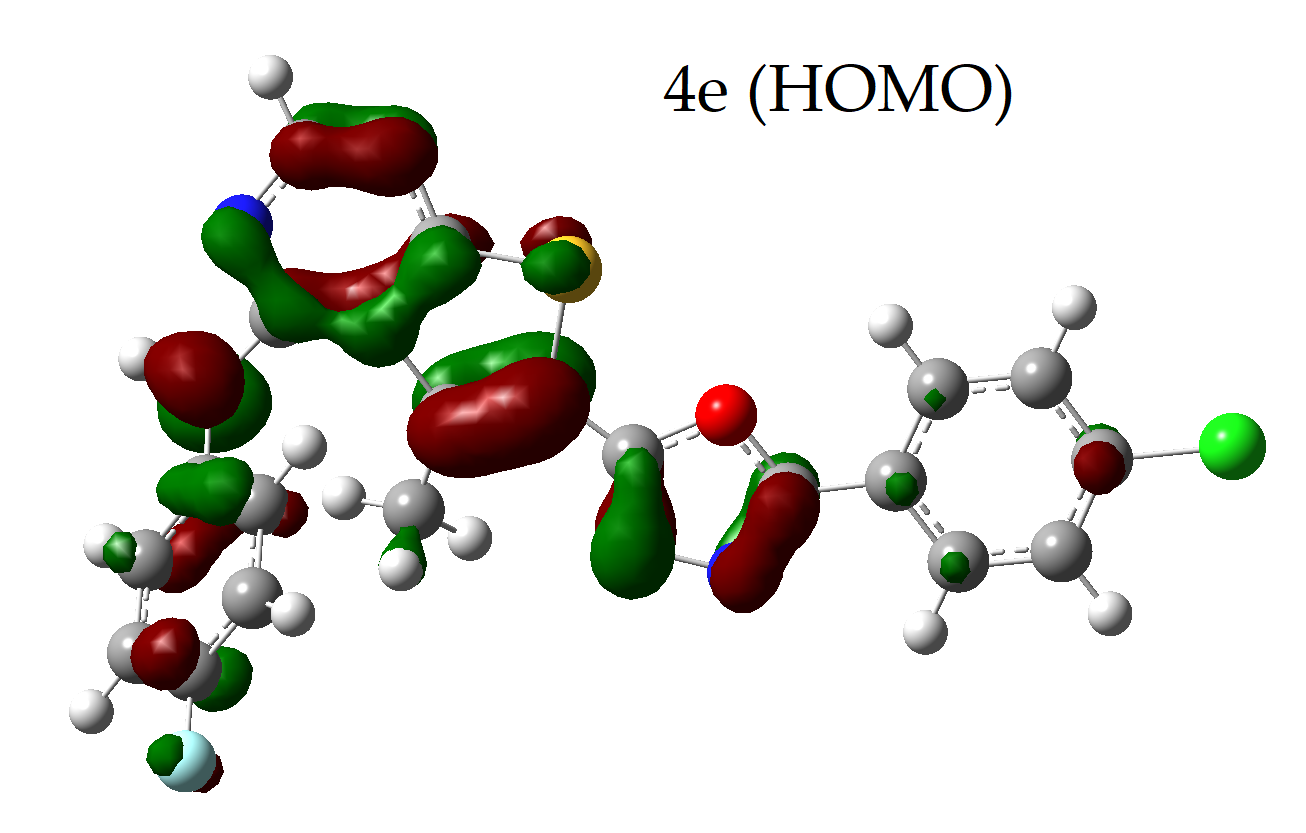 | 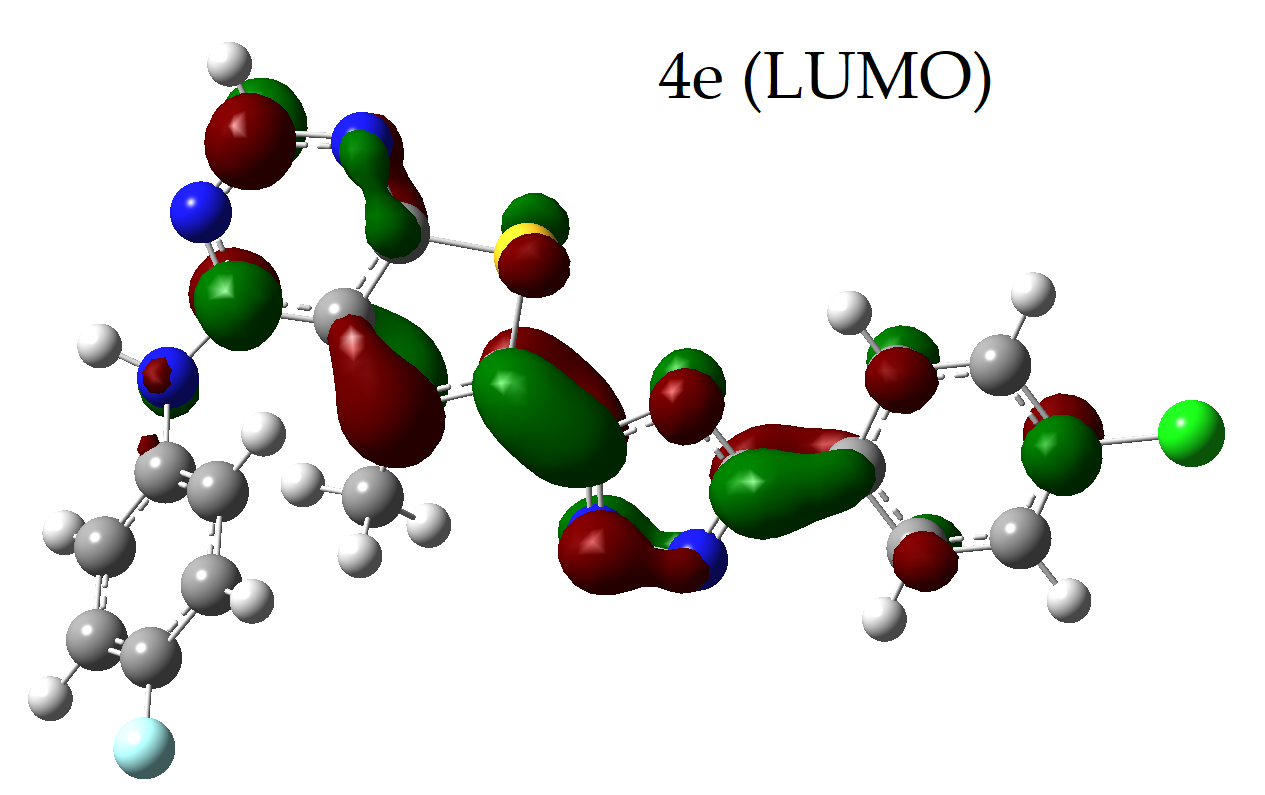 |
| 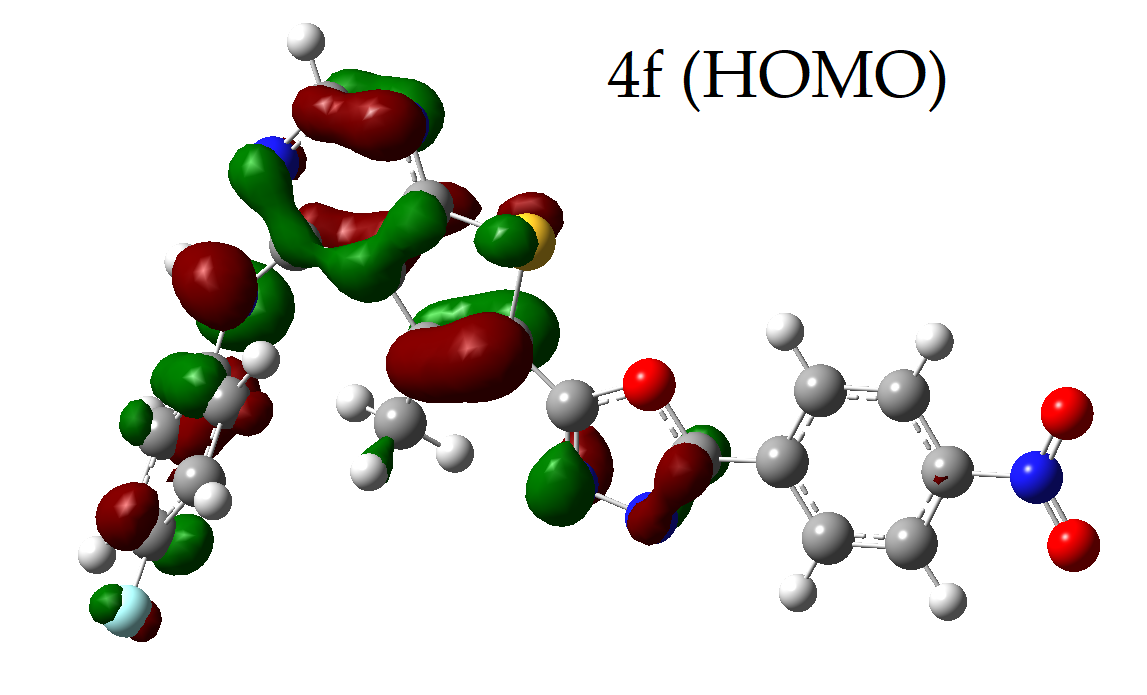 | 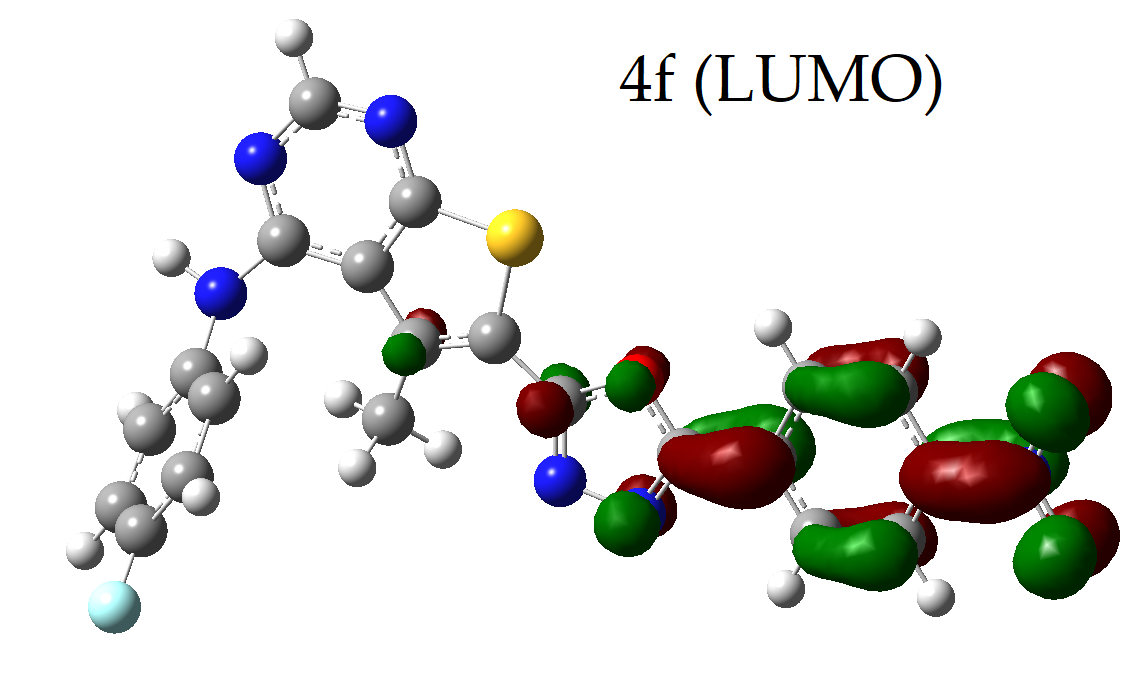 |
| 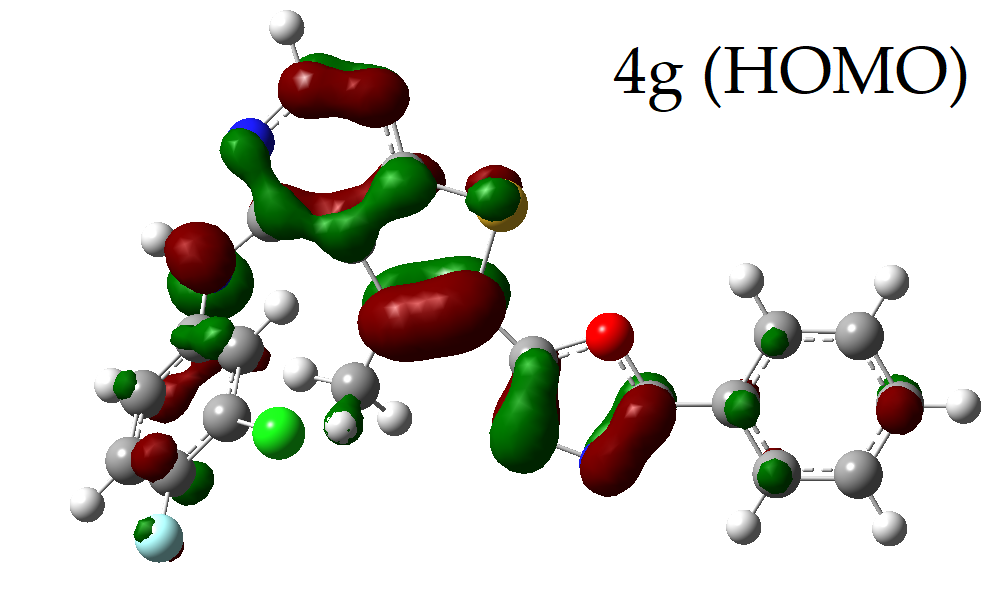 | 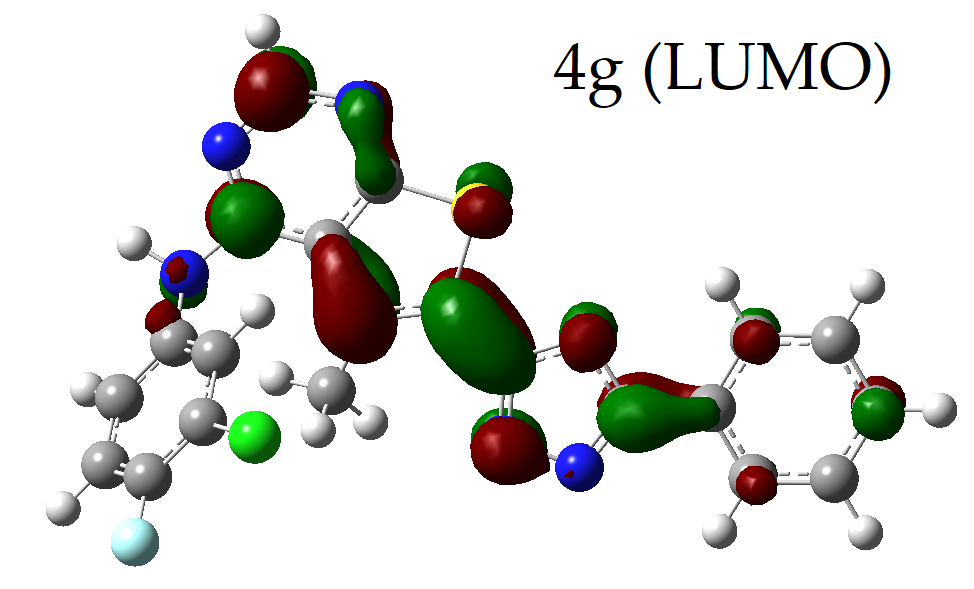 |
| 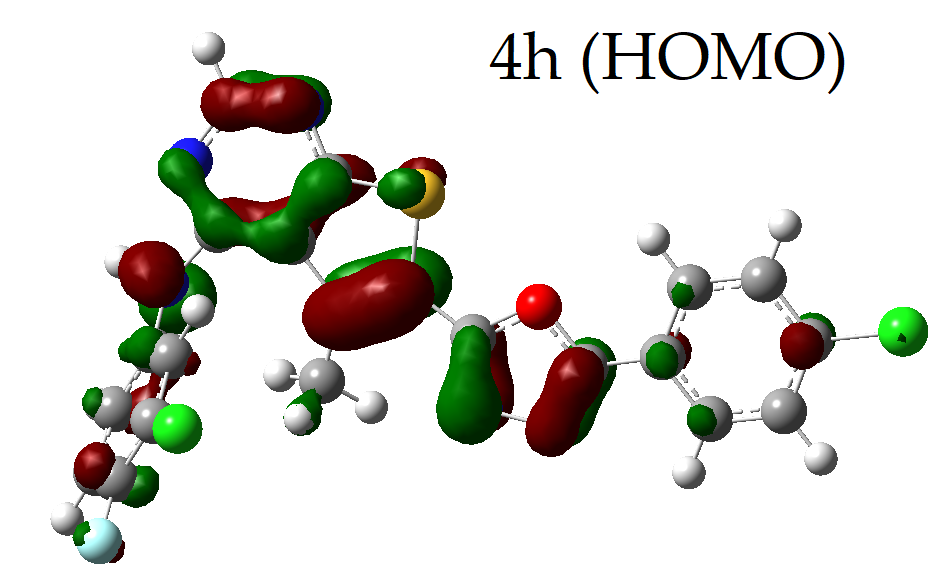 | 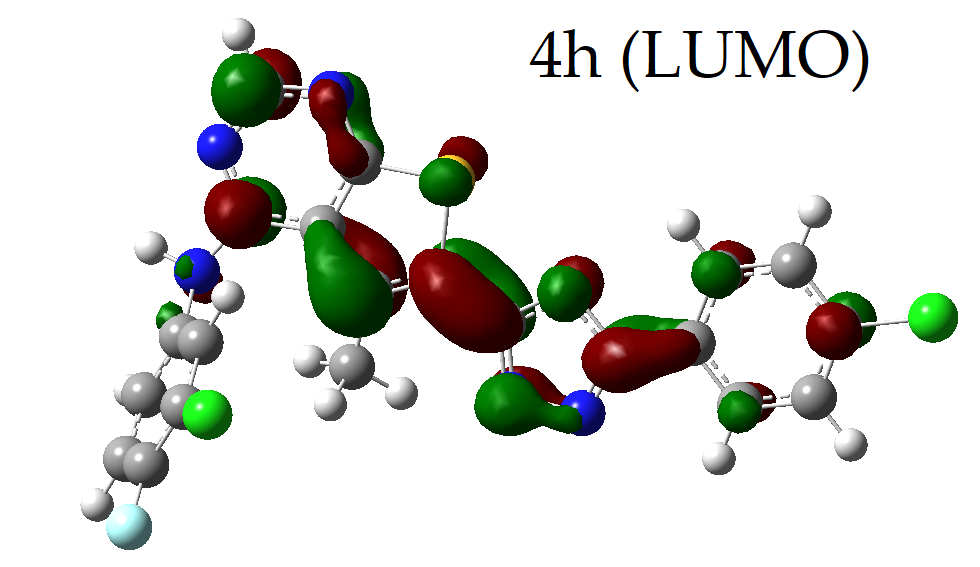 |
| 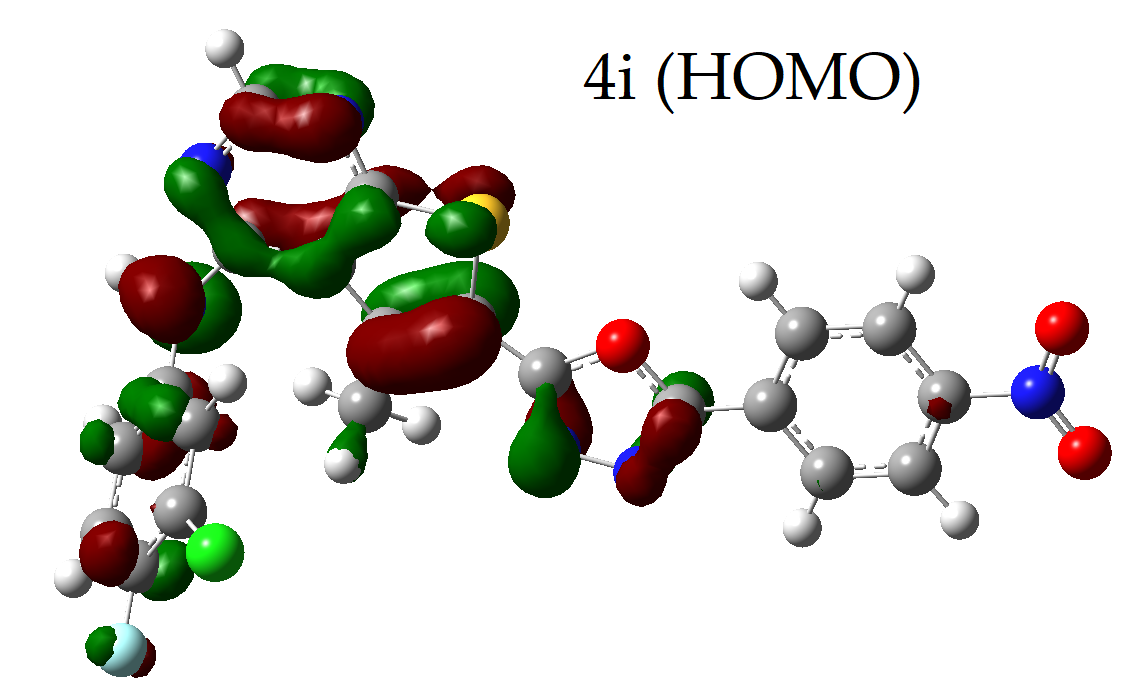 | 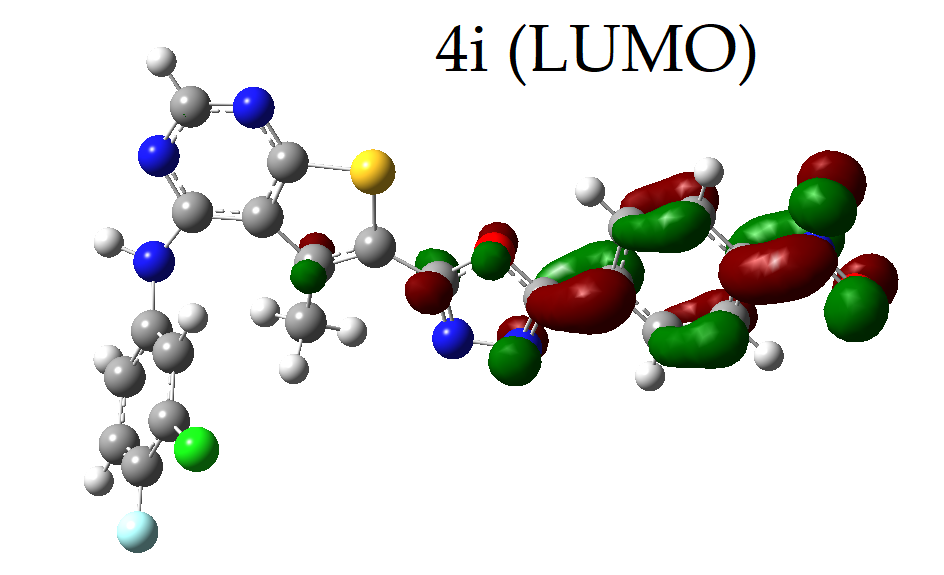 |
| 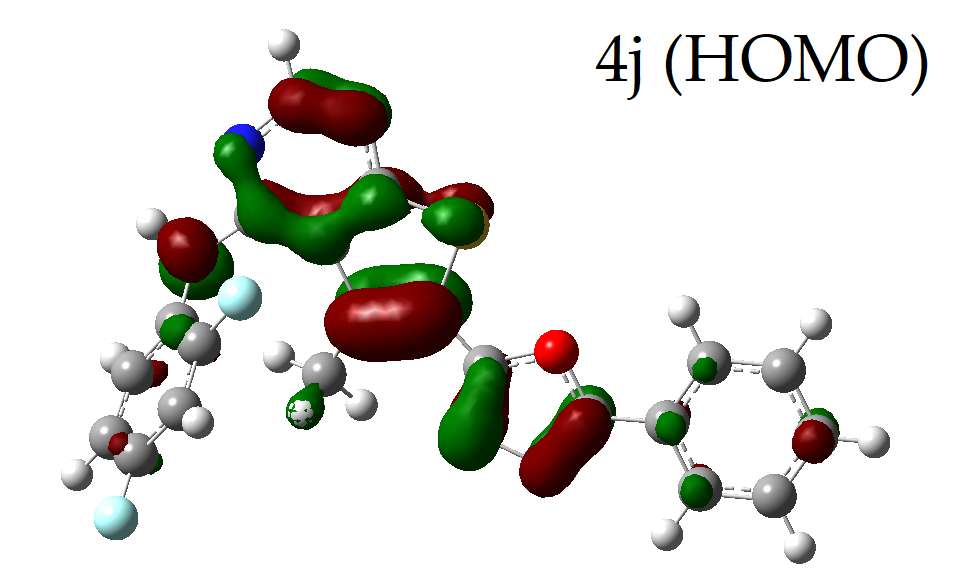 | 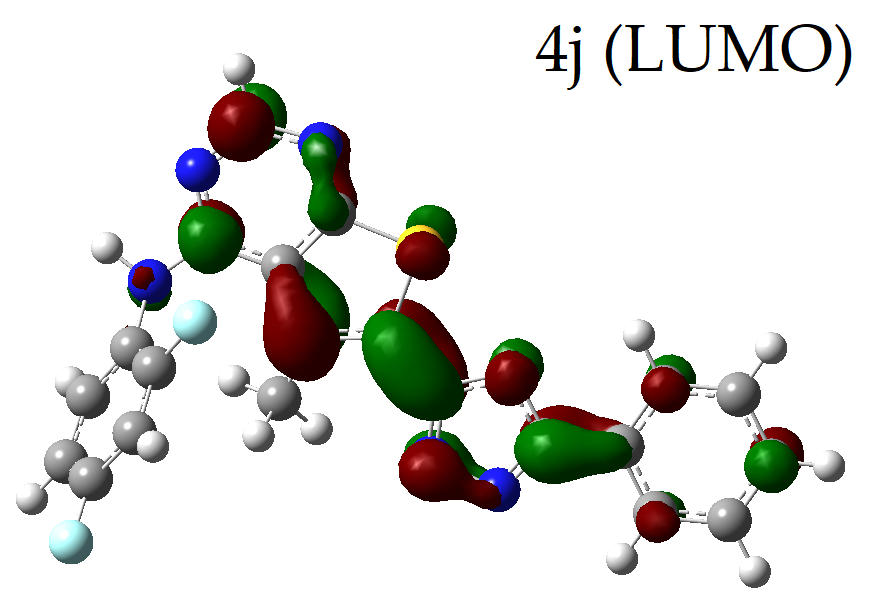 |
| 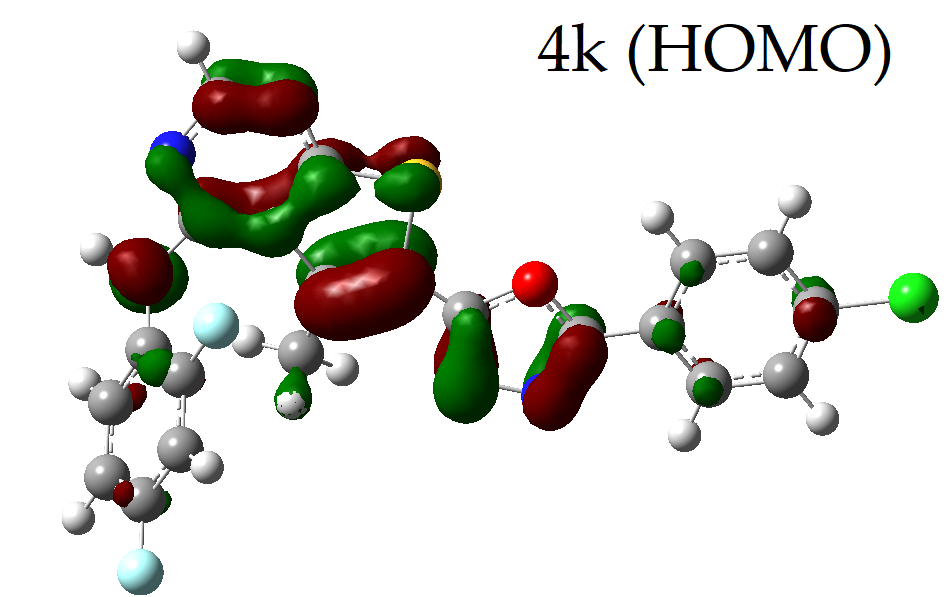 | 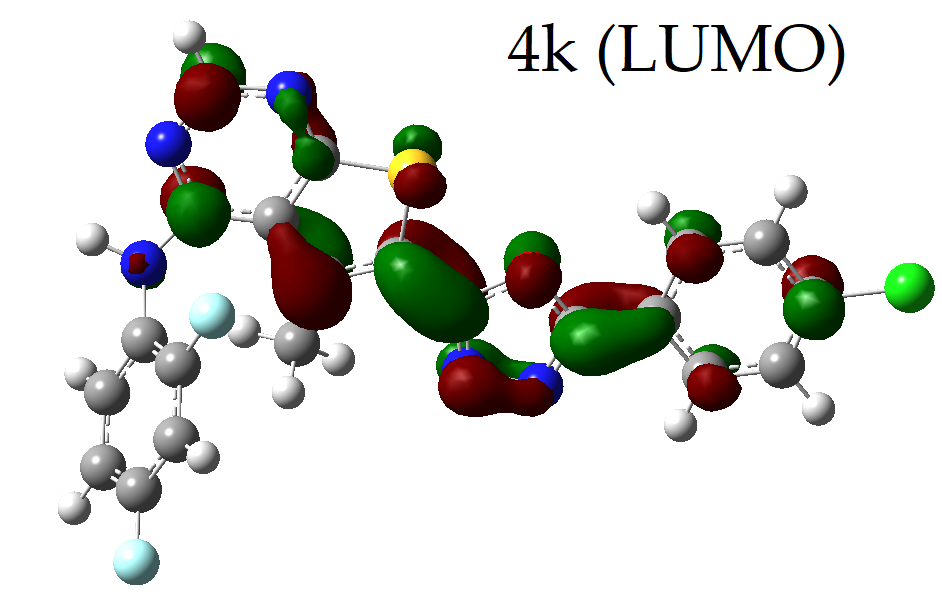 |
| 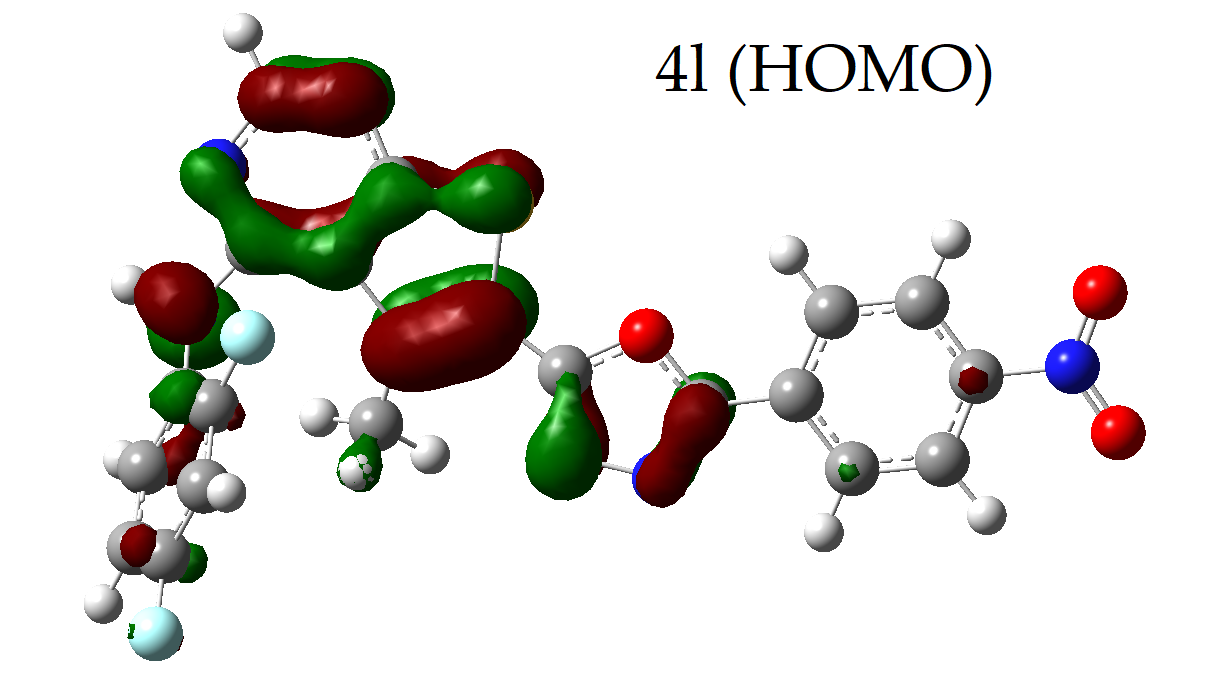 | 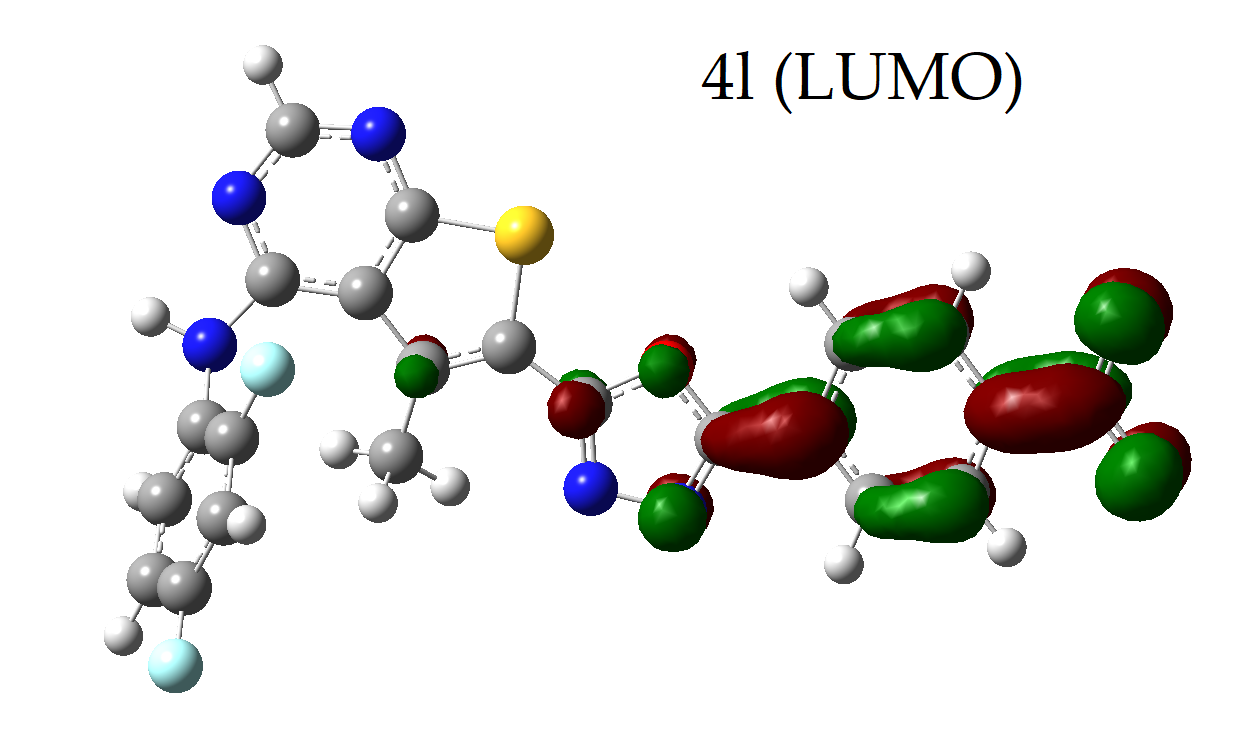 |

**Figure S2:** HOMO and LUMO orbitals of molecules 4a to 4l, are depicted in the gas phase.


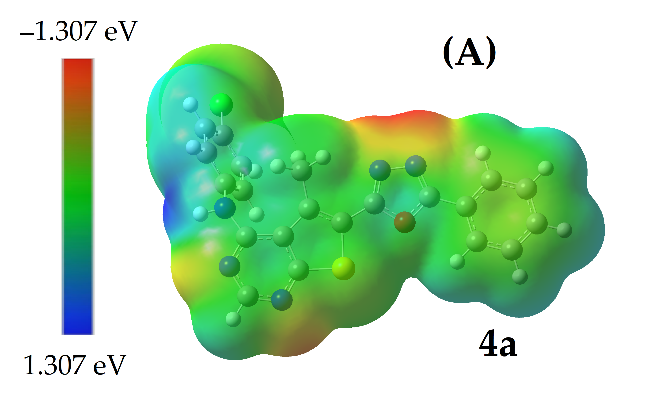

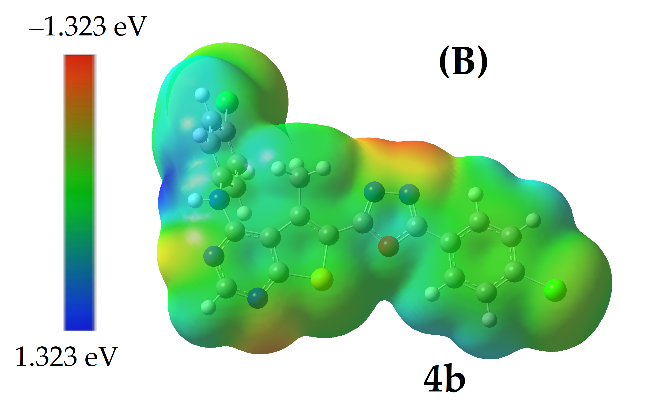

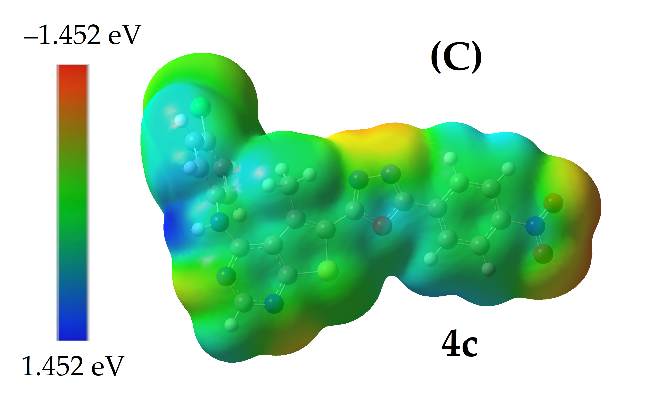

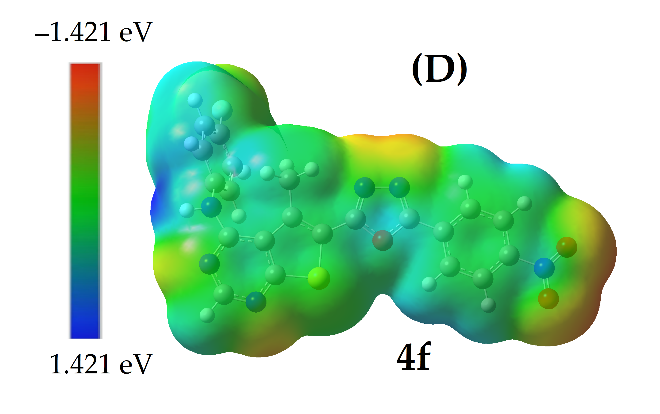

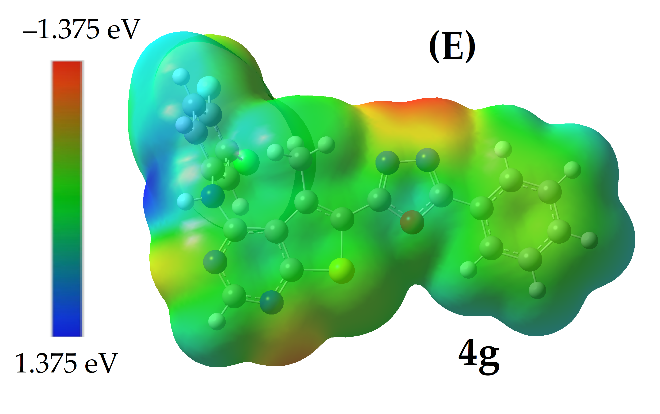

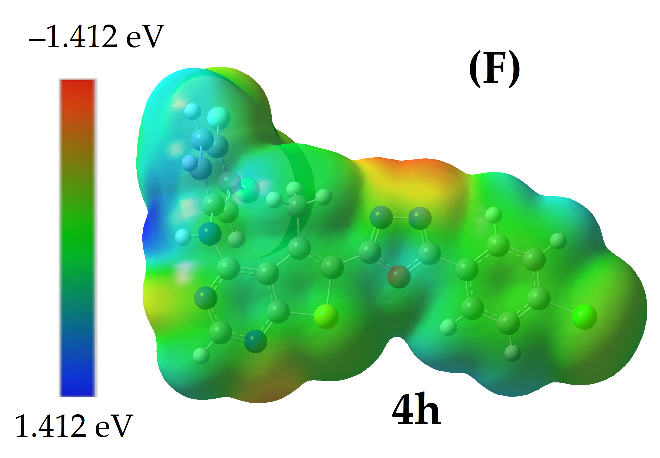

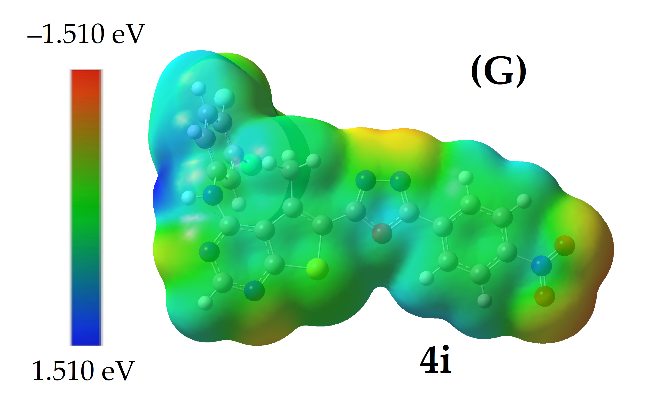

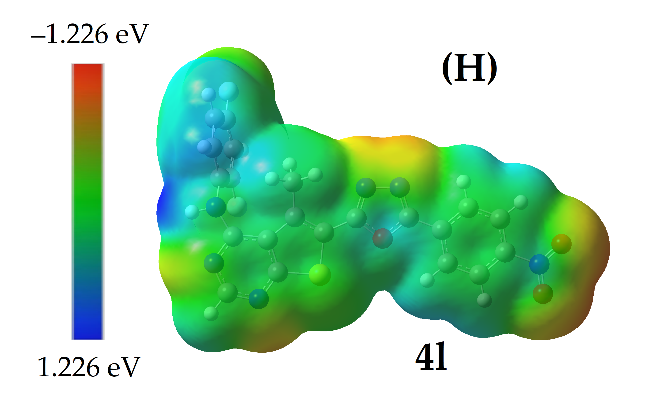


**Figure S3.** MEP isosurfaces of molecules panel (A) compound 4a, (B) compound 4b, (C) compound 4c, (D) compound 4f, (E) compound 4g, (F) compound 4h, (G) compound 4i and (H) compound 4l, that highly potent in experimental results.


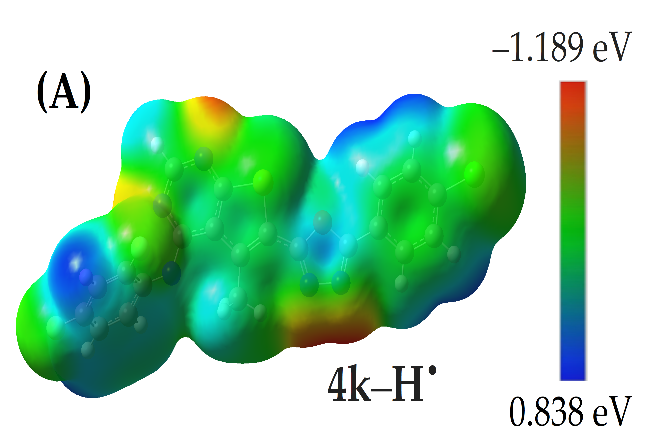

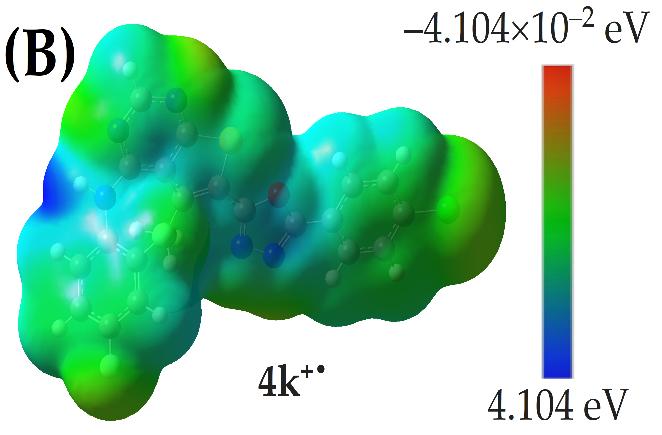


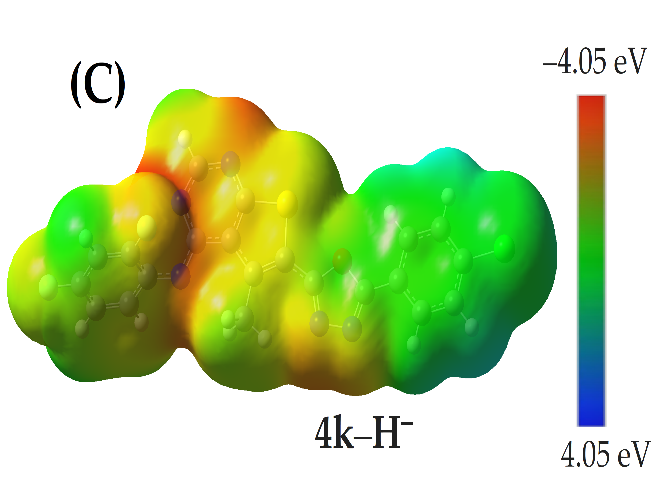

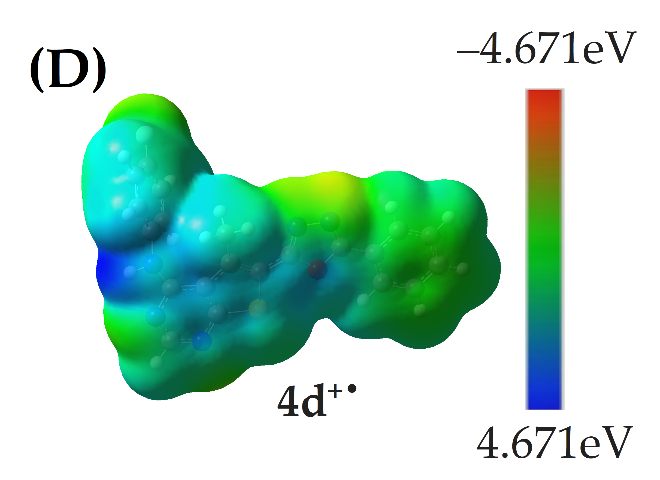

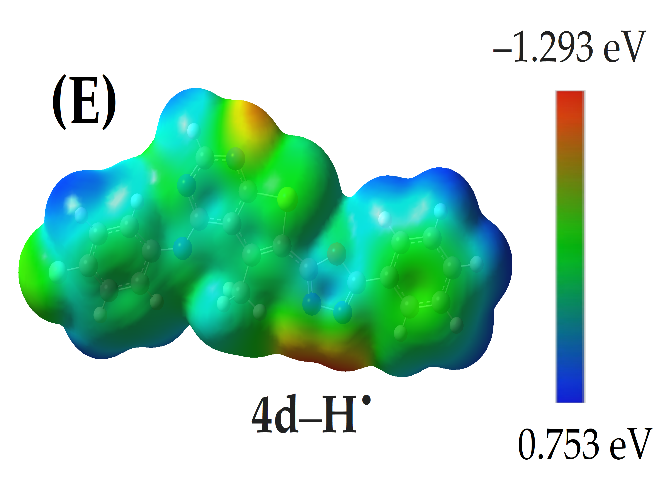

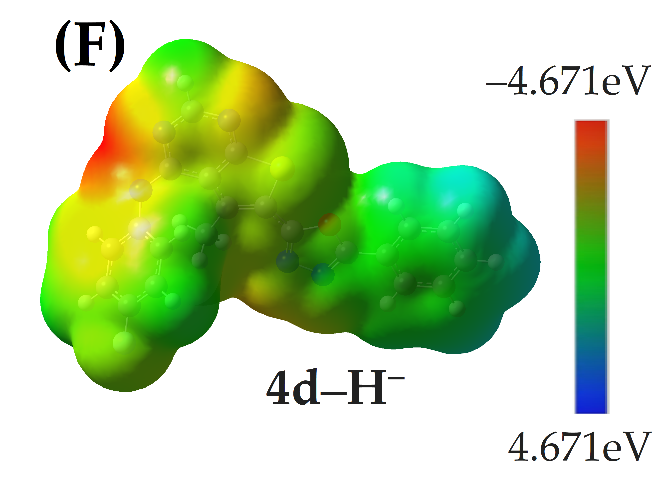


**Figure S4.** MEP isosurfaces of molecules panel (A) compound (4k–H^•^) free radical with abstracted H, (B) compound (4k^+•^) free radical, (C) compound 4k^–^ (anion), (D) compound (4d^+•^) free radical, (E) compound (4k–H^•^) free radical with abstracted H, and (F) compound 4k^–^ (anion), that highly potent in experimental results.

Table S1: The bond dissociation enthalpy (BDE) values of the N−H/C−H bond breaking of 4a to 4l compounds in the gas phase using PM6

| Compounds | Bond numbering | BDE (kcal/mol) PM6 | Molecular Structures |
| --- | --- | --- | --- |
| 4a | C18–H | 95.62455 |  |
|  | N21–H | 71.82567 |  |
|  | C23–H | 96.93558 |  |
|  | C24–H | 96.9759 |  |
|  | C27–H | 97.08678 |  |
|  | C28–H | 96.93495 |  |
|  | C9–H | 95.62644 |  |
|  | C1–H | 94.48551 |  |
|  | C11–H | 94.04703 |  |
|  | C12–H | 94.52142 |  |
|  | C13–H | 96.21108 |  |
| 4b | C19–H | 95.6844 |  |
|  | N22–H | 71.89371 |  |
|  | C9–H | 96.20478 |  |
|  | C10–H | 97.51392 |  |
|  | C13–H | 97.55487 |  |
|  | C14–H | 96.21045 |  |
|  | C24–H | 96.95826 |  |
|  | C25–H | 97.00614 |  |
|  | C28–H | 97.10253 |  |
|  | C29–H | 96.9318 |  |
| 4c | N16–H | 72.20115 |  |
|  | C25–H | 95.84064 |  |
|  | C4–H | 97.78671 |  |
|  | C5–H | 97.31988 |  |
|  | C30–H | 97.91649 |  |
|  | C31–H | 97.83837 |  |
|  | C18–H | 97.01307 |  |
|  | C19–H | 96.48261 |  |
|  | C22–H | 97.14222 |  |
|  | C23–H | 96.92991 |  |
| 4d | C18-H | 95.55966 |  |
|  | N21–H | 71.50689 |  |
|  | C9–H | 95.634 |  |
|  | C10–H | 94.4811 |  |
|  | C11–H | 94.04325 |  |
|  | C12–H | 94.51449 |  |
|  | C13–H | 96.20793 |  |
|  | C23–H | 96.75666 |  |
|  | C24–H | 98.17857 |  |
|  | C28–H | 96.768 |  |
|  | C27–H | 98.29134 |  |
| 4e | N22–H | 71.58249 |  |
|  | C19–H | 95.62077 |  |
|  | C24–H | 96.78564 |  |
|  | C25–H | 98.21574 |  |
|  | C28–H | 98.31654 |  |
|  | C29–H | 96.77052 |  |
|  | C9–H | 96.21234 |  |
|  | C10–H | 97.51077 |  |
|  | C13–H | 97.54857 |  |
|  | C14–H | 96.80202 |  |
| 4f | C19–H | 98.30394 |  |
|  | N16–H | 71.9082 |  |
|  | C4–H | 97.78293 |  |
|  | C5–H | 97.3287 |  |
|  | C30–H | 97.9146 |  |
|  | C31–H | 97.83333 |  |
|  | C18–H | 96.85179 |  |
|  | C25–H | 95.77953 |  |
| 4g | N21–H | 71.40357 |  |
|  | C18–H | 95.72283 |  |
|  | C9–H | 95.60502 |  |
|  | C24–H | 98.35056 |  |
|  | C23–H | 97.0641 |  |
|  | C29–H | 128.05128 |  |
|  | C11–H | 94.04388 |  |
|  | C10–H | 94.48551 |  |
|  | C12–H | 94.52457 |  |
|  | C13–H | 96.20541 |  |
| 4h | N21–H | 71.4924 |  |
|  | C19–H | 95.7852 |  |
|  | C24–H | 97.0893 |  |
|  | C25–H | 98.38521 |  |
|  | C30–H | 127.46412 |  |
|  | C9–H | 96.18084 |  |
|  | C10–H | 97.50825 |  |
|  | C13–H | 97.55235 |  |
|  | C14–H | 96.79761 |  |
| 4i | C19–H | 98.46522 |  |
|  | N16–H | 71.84016 |  |
|  | C4–H | 97.7886 |  |
|  | C5–H | 97.29342 |  |
|  | C32–H | 97.84404 |  |
|  | C31–H | 97.90641 |  |
|  | C18–H | 97.15041 |  |
|  | C26–H | 95.95026 |  |
|  | C24–H | 99.351 |  |
| 4j | C18–H | 95.60313 |  |
|  | N21–H | 73.90215 |  |
|  | C23–H | 97.04961 |  |
|  | C27–H | 102.32208 |  |
|  | C9–H | 95.65416 |  |
|  | C10–H | 94.48362 |  |
|  | C24–H | 99.05868 |  |
|  | C11–H | 94.03695 |  |
|  | C12–H | 94.50252 |  |
| 4k | N22–H | 74.38851 |  |
|  | C24–H | 117.01305 |  |
|  | C25–H | 127.31544 |  |
|  | C28–H | 102.31767 |  |
|  | C9–H | 96.23691 |  |
|  | C19–H | 112.5054 |  |
|  | C10–H | 97.54731 |  |
|  | C13–H | 97.57314 |  |
|  | C14–H | 96.80202 |  |
| 4l | C19–H | 95.87025 |  |
|  | N16–H | 71.31411 |  |
|  | C4–H | 94.46409 |  |
|  | C5–H | 93.99789 |  |
|  | C32–H | 94.50126 |  |
|  | C31–H | 94.55922 |  |
|  | C18–H | 95.52123 |  |
|  | C26–H | 92.14128 |  |
|  | C22–H | 99.05301 |  |
| Ascorbic acid | O1–H | 59.59107 |  |
|  | O12–H | 64.43451 |  |
